# Trends and cross-country inequalities in the global burden of thyroid cancer, 1990–2021: A population-based study

**DOI:** 10.1097/MD.0000000000050237

**Published:** 2026-08-14

**Authors:** Mingxin Shen, Yutong Liu, Liang Shao, Fan Yang, Wei Sun, Hao Zhang

**Affiliations:** a Department of Thyroid Surgery, The First Affiliated Hospital of China Medical University, Shenyang, Liaoning, China; b Department of Obstetrics and Gynecology, Shengjing Hospital of China Medical University, Shenyang, China.

**Keywords:** deaths, disability-adjusted life-years, health inequalities, incidence, prevalence, thyroid cancer, trends

## Abstract

**Background:**

To evaluate the trends in the burden of thyroid cancer (TC) over the past 30 years, assess inequalities between countries, and predict changes by 2045.

**Methods:**

TC incidence, prevalence, deaths, and disability-adjusted life years (DALYs), with 95% uncertainty intervals (UIs), were obtained from the Global Burden of Diseases, Injuries, and Risk Factors Study 2021. We analyzed global, regional, and national trends during 1990–2021. Decomposition analysis assessed population growth, aging, and epidemiological change. Health inequalities were quantified using World Health Organization-recommended methods, and burden was projected to 2045.

**Results:**

In 2021, estimates were 249,538 (95% UI: 223,290–274,638) incident cases, 1,987,148 (1,776,275–2,198,245) prevalent cases, 44,799 (39,925–48,541) deaths, and 1,246,485 (1,094,416–1,375,853) DALYs globally. East Asia had the most cases, whereas high-income North America had the highest age-standardized rate (ASR). During 1990 to 2021, TC burden increased, with incidence, prevalence, and deaths rising fastest during 2000 to 2010. Females (53.51%) and middle-Socio-demographic Index (SDI) regions (41.69%) accounted for the largest DALY proportions. DALY changes were driven mainly by population growth, followed by aging. SDI-related inequalities increased: the incidence gap between the highest- and lowest-SDI countries rose from 2.63 (95% confidence interval: 2.28–2.98) in 1990 to 4.58 (4.04–5.13) in 2021, and the prevalence gap from 20.17 (17.31–23.02) to 36.96 (32.26–41.65). During 2020–2045, incidence and prevalence numbers and ASRs are projected to increase annually. Although death ASRs are expected to decline, death counts will rise. In 2045, incident cases, prevalent cases, and deaths are projected to reach 449,910 (95% UI: 45,706–884,520), 3,295,386 (310,305–6,488,732), and 78,818 (27,472–130,171), respectively.

**Conclusions:**

Global TC burden increased during 1990–2021, mainly because of population growth and aging. High-SDI countries bear a disproportionate burden, and inequalities have widened. Rising cases and unequal distribution challenge TC control and management. These findings may inform public health policy and equitable resource allocation.

## 1. Introduction

The global incidence of thyroid cancer (TC) continues to rise, ranking ninth among all cancers worldwide as of 2020,^[1]^ making it one of the most common malignancies of the endocrine system.^[2]^ In 2019, there were a total of 233,847 new cases of TC globally, resulting in 45,576 deaths, which has led to a significant health burden and economic cost.^[3]^ With the trends of global population aging and rapid growth, the epidemiological changes of TC may also be significantly impacted, and the disease burden caused by TC urgently requires more attention. The risk of developing TC is significantly higher in females, who account for approximately 75% of all TC patients, indicating that the female population needs more focused attention and thorough screening.^[4,5]^ TC can occur at any age, with adults aged 30 to 60 years considered the high-risk age group for TC, suggesting the need for targeted screening strategies across different age groups. Reliable global predictions are essential for effectively quantifying the burden and long-term impacts of TC and are key to optimizing healthcare services, improving prevention strategies, and formulating appropriate health policies.

The Global Burden of Diseases, Injuries, and Risk Factors Study (GBD) is a large-scale observational epidemiological project that covers the globe and aims to systematically evaluate 371 disease indicators, including incidence, prevalence, deaths, and disability-adjusted life-years (DALY) rates stratified by age, sex, region, and time.^[6]^ Although previous studies utilized 2019 global biological data to analyze the burden of TC,^[7,8]^ the GBD database has recently been updated to include data from 2020 and 2021, making this data more representative and current. Here, we utilize the 2021 GBD data to provide a comprehensive assessment of the burden, trends, and regional inequalities of TC.^[9]^ Specifically, this study includes descriptive analyses of TC epidemiology at global, regional, and national levels; trend analyses from an overall, localized, and multidimensional perspective; decomposition analyses based on demographic and epidemiological factors; cross-national inequality analyses using the standard health equity analysis methods recommended by the World Health Organization; analyses of health inequalities related to TC at global and regional levels; and predictive analyses of the global TC burden up to 2045.

## 2. Methods

### 2.1. Data resource

The 2021 GBD provides a detailed assessment of the health impact of 371 diseases and injuries, as well as 88 risk factors across 204 countries and regions, based on the latest epidemiological data and improved standardization methods. Data were downloaded from the Global Health Data Exchange query tool (http://ghdx.healthdata.org/gbdresults-tool), categorized by region, sex, country, and risk. The GBD dataset serves as a vital global health resource, enabling meticulous tracking of changes in diseases, injuries, and risk factors and their status across different regions.^[6]^ We extracted data on incidence, DALY, deaths, age-standardized incidence rates, age-standardized DALY rates, age-standardized mortality rates, and percentage changes in age-standardized rates (ASRs) from the GBD database. All rates are expressed per 100,000 person-years. The Socio-demographic Index (SDI) is a composite indicator calculated based on factors such as per capita gross domestic product, education levels, and total fertility rates, which can be used to measure the socioeconomic, demographic, and development levels of different countries and regions. The SDI values typically range from 0 to 1, with higher values indicating greater socioeconomic and development levels in the region, and lower values indicating the opposite. The 204 countries and regions were categorized into 5 groups based on their SDI: high SDI, upper-middle SDI, middle SDI, lower-middle SDI, and low SDI quintiles.^[10]^ By comparing the SDI values of different countries and regions, researchers and policymakers can gain a better understanding of the overall development and health status across various areas. The current GBD estimates are based on the methodologies described in the latest GBD, and a brief overview is provided below. All statistical analyses and visualizations were performed using R software version 4.4.1.

### 2.2. Descriptive analysis

To comprehensively understand the burden of TC, descriptive analyses were conducted at global, regional, and national levels. The incidence, prevalence, deaths, and number of DALY associated with TC from 1990 to 2021 were visually represented, along with their ASR. Additionally, we compared the differences in the number of cases and ASR for incidence, prevalence, deaths, and DALY of TC between 1990 and 2021 across the globe, 5 SDI quintile regions, and 21 GBD geographical regions. Furthermore, we analyzed the incidence, prevalence, deaths, and ASR for 204 countries and regions worldwide in 2021.

### 2.3. Trend analysis

Exploring the temporal trends of diseases is one of the most important research methods in epidemiological studies. Investigating the development trends of TC allows policymakers to examine the trends from overall and localized multidimensional perspectives, facilitating the development of more precise prevention strategies and providing important theoretical support for implementation.

Firstly, we utilized the estimated annual percentage change (EAPC) to quantify the overall trends in the burden of TC.^[11]^ The trends in ASR assessed by EAPC are considered a more robust indicator for monitoring changes in disease patterns. The EAPC in ASR of TC were calculated based on sex, region, and etiology to quantify the temporal trends of various indicators related to TC. The EAPC calculation employed a linear regression of the log-transformed ASR: *y* = α+β*x*+ϵ, where *y* = ln(ASR), and *x* represents the calendar year. The EAPC calculation method is given by (exp(β) − 1)* 100%, with a linear regression model used to determine the 95% confidence interval (CI).^[12]^ If both the EAPC estimate and the lower limit of its 95% CI are >0, the ASR is considered to be exhibiting an upward trend. Conversely, if both the EAPC estimate and the upper limit of its 95% CI are <0, the ASR is regarded as exhibiting a downward trend. Otherwise, the ASR is considered stable.

Additionally, we explored the trends in the burden of TC associated with age, period, and birth cohort effects. Due to the complete linear relationship among age, period, it is statistically impossible to estimate the independent effects of age, period, and cohort.^[13]^ Therefore, an age-period-cohort model that incorporates all 3 temporal dimensions was employed to estimate the effects of age, period, and birth cohort on the risks of incidence and prevalence. In this model, the age effect reflects changes in the variable over an individual’s lifetime; the period effect represents the impact of environmental factors affecting the entire population; and the cohort effect refers to the variations in the variable for groups of individuals who experienced similar life events due to being born in the same year.^[14]^

We utilized a principal component regression analysis of the age-period-cohort model and an intrinsic estimator method based on the Poisson distribution to address the identification issues arising from the multicollinearity among age, period, and birth cohort.^[15]^ This approach allows for the interpretation of the distinct impacts of the 3 temporal trends and provides relatively effective estimates. Moreover, the data were recoded into continuous 5-year age groups (10–14 years, 15–19 years, ..., 95–99 years) and grouped into continuous 5-year periods from 1992 to 2021 (1992–1996, 1997–2001, 2002–2006, ..., 2012–2016, 2017–2021), with corresponding continuous 5-year birth cohorts generated (1897–1901, 1902–1906, ..., 2002–2006, 2007–2011) to estimate the net age, period, and cohort effects on the incidence and prevalence of TC. The age-period-cohort model and intrinsic estimator method provided estimated coefficients for the age, period, and cohort effects, which were transformed into exponentiated values to determine the relative risks (RR) of incidence and prevalence for specific ages, periods, or birth cohorts in relation to the average merged levels across all ages, periods, or cohorts.^[16]^

### 2.4. Decomposition analysis

To gain a deeper understanding of the explanatory factors driving changes in the DALY burden of TC from 1990 to 2021, we employed Das Gupta decomposition analysis method to analyze the contributing factors of TC burden based on population size, age structure, and epidemiological changes.^[17]^ This analysis aims to quantify the individual effects of population growth, aging, and epidemiological changes, necessitating the separate assessment of each factor’s contribution while keeping the other 2 factors constant.^[18]^ Additionally, to understand whether the ratios of the contributing factors to the TC burden are influenced by gender differences, we further decomposed the DALY of TC by gender (male and female) into subgroups for elucidation.

### 2.5. Cross-country inequality analysis

Cross-country inequality analysis provides a foundation for evidence-based health planning and further improves related policies, programs, and practices to reduce disparities in health distribution. In this study, we utilized the inequality slope index^[19]^ and the concentration index^[20]^ to measure the distributional inequality of the TC burden between countries. The inequality slope index is derived from regression analysis, linking the DALY of all age groups in a country to their relative position in socio-demographic development, which is determined by the midpoint of the cumulative range of populations sorted by SDI. A weighted regression model was employed to account for heteroscedasticity. The calculation of the health inequality concentration index involves fitting a Lorenz concentration curve to the observed cumulative relative distribution of the population ranked by SDI and the DALY burden of the disease, followed by numerical integration of the area under the curve.^[21]^

### 2.6. Predictive analysis

The above analyses focus on the burden of TC over the past few decades. To better inform public health policy and allocate health resources, we conducted further predictions regarding the burden of TC in the coming decades. The Markov chain in Bayesian inference is a well-known method.^[22]^ However, this approach is not very efficient. The integrated nested Laplace approximation method serves as a low-computational-density alternative to Markov chains for approximating Bayesian inference in latent Gaussian models, addressing several issues related to mixing, convergence, and inefficiency associated with Markov chain Monte Carlo sampling techniques.^[23]^ Therefore, we employed the integrated nested Laplace approximation with Bayesian age-period-cohort modeling to predict the global burden of TC up to 2045.^[23]^ This method offers improved coverage and accuracy compared to traditional annual percentage change models.

## 3. Results

### 3.1. Descriptive analysis of TC burden at global, regional, and national levels

The incidence, prevalence, deaths, and DALY case numbers and ASR of TC for the years 1990 and 2021 are presented in Tables 1 to 4. Globally, the number of cases and age-standardized rates (ASRs) of incidence and prevalence of TC showed a significant increasing trend, while the number of deaths and DALY associated with TC significantly increased from 1990 to 2021. In contrast, the mortality rate and DALY rate of TC exhibited a declining trend. Regarding gender, the case numbers and rates for these 4 indicators were higher in females compared to males (Tables 1–4).

**Table 1:** The case number and ASR of incidence of TC in 1990 and 2021 for both sexes by SDI quintiles and by GBD regions, with EAPC and AAPC from 1990 to 2021.

Location	1990		2021		EAPC	AAPC
	Number (95% UIs)	ASR (95% UIs)	Number (95% UIs)	ASR (95% UIs)		
Global	89,885 (84,681 to 96,999)	2.06 (1.95 to 2.22)	249,538 (223,290 to 274,638)	2.91 (2.61 to 3.21)	1.25 (1.14 to 1.37)	1.14 (1.04 to 1.24)
Gender						
Male	25,306 (24,146 to 27,282)	1.22 (1.17 to 1.31)	82,301 (71,575 to 91,093)	1.98 (1.72 to 2.19)	1.78 (1.64 to 1.92)	1.61 (1.43 to 1.80)
Female	64,579 (59,813 to 71,524)	2.86 (2.66 to 3.16)	167,237 (147,083 to 195,647)	3.83 (3.36 to 4.49)	1.03 (0.92 to 1.14)	0.95 (0.86 to 1.03)
SDI quintiles						
High SDI	36,533 (35,292 to 37,708)	3.55 (3.43 to 3.66)	72,996 (68,514 to 76,747)	4.49 (4.25 to 4.75)	1.01 (0.75 to 1.28)	0.82 (0.68 to 0.96)
High-middle SDI	24,411 (22,753 to 25,920)	2.35 (2.19 to 2.49)	55,158 (49,518 to 62,489)	3.07 (2.75 to 3.49)	1.05 (0.89 to 1.21)	0.87 (0.61 to 1.12)
Middle SDI	17,155 (15,283 to 19,997)	1.35 (1.21 to 1.60)	75,357 (62,756 to 84,675)	2.71 (2.26 to 3.05)	2.37 (2.28 to 2.46)	2.29 (2.2 to 2.38)
Low-middle SDI	8234 (7035 to 10,302)	1.05 (0.91 to 1.31)	33,464 (27,896 to 40,293)	1.96 (1.65 to 2.34)	2.09 (2.07 to 2.12)	2.03 (1.91 to 2.15)
Low SDI	3431 (2759 to 4296)	1.13 (0.91 to 1.42)	12,358 (9599 to 16,515)	1.69 (1.33 to 2.20)	1.23 (1.12 to 1.34)	1.3 (1.2 to 1.4)
GBD regions						
Central Asia	914 (841 to 996)	1.73 (1.60 to 1.89)	1631 (1432 to 1845)	1.75 (1.54 to 1.97)	0.01 (−0.73 to 0.75)	−0.12 (−1.56 to 1.34)
Central Europe	4655 (4429 to 4884)	3.20 (3.04 to 3.36)	4876 (4407 to 5323)	2.71 (2.44 to 2.95)	−0.65 (−0.86 to −0.45)	−0.61 (−1.24 to 0.02)
Eastern Europe	6468 (6164 to 6831)	2.43 (2.31 to 2.56)	9617 (8698 to 10,650)	3.20 (2.90 to 3.55)	1.27 (0.8 to 1.75)	0.66 (−0.15 to 1.48)
Australasia	586 (522 to 660)	2.61 (2.32 to 2.94)	1949 (1570 to 2343)	4.57 (3.70 to 5.54)	2.61 (2.06 to 3.16)	1.86 (0.97 to 2.75)
High-income Asia Pacific	6950 (6496 to 7654)	3.42 (3.19 to 3.77)	14,278 (12,630 to 16,477)	4.40 (3.94 to 5.15)	1.15 (0.68 to 1.61)	0.82 (0.34 to 1.31)
High-income North America	12,130 (11,695 to 12,450)	3.81 (3.68 to 3.91)	28,289 (26,783 to 29,536)	5.30 (5.07 to 5.53)	1.15 (0.97 to 1.33)	1.13 (0.34 to 1.93)
Southern Latin America	1000 (896 to 1119)	2.13 (1.91 to 2.38)	2047 (1787 to 2334)	2.54 (2.22 to 2.90)	0.7 (0.48 to 0.92)	0.58 (−0.18 to 1.34)
Western Europe	19,206 (18,292 to 20,159)	3.88 (3.69 to 4.08)	26,005 (23,788 to 28,201)	3.84 (3.51 to 4.18)	0.28 (−0.1 to 0.67)	−0.05 (−0.48 to 0.37)
Andean Latin America	422 (354 to 495)	1.76 (1.47 to 2.05)	2424 (1907 to 3044)	3.87 (3.05 to 4.85)	2.6 (2.44 to 2.77)	2.82 (2.58 to 3.06)
Caribbean	443 (411 to 479)	1.56 (1.45 to 1.69)	1245 (1086 to 1427)	2.36 (2.06 to 2.70)	1.5 (1.32 to 1.69)	1.46 (1.21 to 1.71)
Central Latin America	1712 (1651 to 1775)	1.71 (1.65 to 1.78)	7753 (6907 to 8701)	2.96 (2.64 to 3.32)	1.66 (1.52 to 1.8)	1.75 (1.42 to 2.08)
Tropical Latin America	1372 (1299 to 1443)	1.28 (1.22 to 1.35)	4491 (4198 to 4758)	1.73 (1.61 to 1.83)	0.76 (0.6 to 0.91)	0.93 (0.79 to 1.07)
North Africa and Middle East	3792 (3151 to 5143)	1.69 (1.41 to 2 to 30)	21,222 (17,602 to 24,975)	3.65 (3.05 to 4.27)	2.89 (2.72 to 3.06)	2.52 (2.38 to 2.67)
South Asia	7853 (6476 to 10,285)	1.01 (0.84 to 1.32)	37,336 (30,263 to 44,931)	2.14 (1.74 to 2.56)	2.54 (2.46 to 2.62)	2.44 (2.35 to 2.54)
East Asia	13,203 (10,809 to 15,461)	1.30 (1.07 to 1.52)	50,885 (41,562 to 63,162)	2.53 (2.07 to 3.14)	2.43 (2.26 to 2.6)	2.19 (2.07 to 2.31)
Oceania	45 (30 to 60)	1.19 (0.83 to 1.59)	131 (80 to 185)	1.36 (0.85 to 1.87)	0.33 (0.24 to 0.42)	0.42 (0.23 to 0.61)
Southeast Asia	6441 (5206 to 7277)	2.02 (1.68 to 2.32)	26,559 (20,899 to 31,184)	3.61 (2.86 to 4.24)	1.82 (1.74 to 1.9)	1.9 (1.78 to 2.03)
Central sub-Saharan Africa	154 (112 to 226)	0.57 (0.41 to 0.82)	496 (319 to 771)	0.69 (0.45 to 1.09)	0.63 (0.42 to 0.83)	0.63 (0.56 to 0.7)
Eastern sub-Saharan Africa	1908 (1517 to 2388)	1.81 (1.46 to 2.24)	6384 (4630 to 9479)	2.41 (1.77 to 3.45)	0.76 (0.6 to 0.92)	0.94 (0.88 to 0.99)
Southern sub-Saharan Africa	377 (318 to 448)	1.09 (0.91 to 1.30)	1117 (935 to 1316)	1.61 (1.34 to 1.88)	1.54 (1.3 to 1.79)	1.32 (0.82 to 1.82)
Western sub-Saharan Africa	254 (186 to 312)	0.22 (0.16 to 0.26)	804 (598 to 1069)	0.26 (0.20 to 0.34)	0.52 (0.45 to 0.58)	0.58 (0.44 to 0.72)

AAPC = average annual percentage change, ASR = age-standardized rate, EAPC = estimated annual percentage change, GBD = Global Burden of Diseases, Injuries, and Risk Factors Study, SDI = Socio-demographic Index, TC = thyroid cancer, UIs = uncertainty intervals.

**Table 2:** The case number and ASR of prevalence of TC in 1990 and 2021 for both sexes by SDI quintiles and by GBD regions, with EAPC and AAPC from 1990 to 2021.

Location	1990		2021		EAPC	AAPC
	Number(95% UIs)	ASR(95% UIs)	Number(95% UIs)	ASR(95% UIs)		
Global	676,649 (636,789 to 727,723)	14.93 (14.12 to 16.03)	1,987,148 (1,776,275 to 2,198,245)	23.14 (20.66 to 25.65)	1.58 (1.44 to 1.73)	1.42 (1.31 to 1.52)
Gender						
Male	180,150 (172,642 to 191,834)	8.08 (7.75 to 8.58)	627,658 (549,745 to 698,427)	14.79 (12.96 to 16.44)	2.23 (2.05 to 2.41)	2.00 (1.70 to 2.30)
Female	496,499 (459,972 to 547,527)	21.62 (20.07 to 23.73)	1,359,490 (1,199,808 to 1,597,307)	31.33 (27.56 to 36.85)	1.32 (1.19 to 1.45)	1.20 (1.09 to 1.31)
SDI						
High SDI	288,686 (280,129 to 297,109)	28.45 (27.60 to 29.28)	598,370 (566,882 to 629,719)	38.16 (36.32 to 40.37)	1.23 (0.93 to 1.53)	1.01 (0.87 to 1.15)
High-middle SDI	186,568 (174,098 to 198,523)	17.55 (16.39 to 18.65)	448,570 (401,405 to 510,752)	25.22 (22.58 to 28.81)	1.4 (1.22 to 1.58)	1.18 (0.92 to 1.45)
Middle SDI	122,505 (108,258 to 142,673)	8.65 (7.67 to 10.10)	594,136 (493,697 to 673,492)	21.02 (17.46 to 23.84)	3.03 (2.95 to 3.12)	2.92 (2.81 to 3.04)
Low-middle SDI	56,029 (47,678 to 71,121)	6.26 (5.35 to 7.89)	251,683 (206,843 to 308,118)	13.95 (11.51 to 16.94)	2.67 (2.64 to 2.7)	2.59 (2.4 to 2.78)
Low SDI	21,972 (17,309 to 27,987)	6.15 (4.87 to 7.89)	92,787 (71,790 to 126,590)	11.14 (8.66 to 14.93)	1.87 (1.76 to 1.99)	1.92 (1.84 to 1.99)
Region						
Central Asia	6946 (6348 to 7593)	12.71 (11.60 to 13.93)	13,021 (11,400 to 14,782)	13.38 (11.74 to 15.15)	0.16 (−0.61 to 0.93)	0.02 (−1.47 to 1.54)
Central Europe	34,174 (32,484 to 36,025)	23.56 (22.39 to 24.81)	37,892 (34,074 to 41,468)	21.93 (19.71 to 23.98)	−0.33 (−0.52 to −0.14)	−0.33 (−1.02 to 0.37)
Eastern Europe	50,163 (47,848 to 52,998)	18.91 (18.06 to 20.00)	75,906 (68,505 to 84,344)	25.90 (23.34 to 28.76)	1.47 (0.97 to 1.97)	0.74 (−0.17 to 1.66)
Australasia	4600 (4077 to 5211)	20.63 (18.28 to 23.34)	16,211 (13,091 to 19,621)	38.93 (31.44 to 47.57)	2.87 (2.29 to 3.47)	2.1 (1.2 to 3.02)
High-income Asia Pacific	55,345 (51,671 to 60,633)	26.92 (25.14 to 29.57)	110,959 (98,932 to 129,335)	37.09 (33.17 to 43.84)	1.42 (0.91 to 1.93)	1.05 (0.57 to 1.53)
High-income North America	100,673 (97,446 to 103,313)	32.05 (31.06 to 32.89)	237,732 (226,609 to 247,666)	45.47 (43.57 to 47.33)	1.23 (1.05 to 1.42)	1.21 (0.36 to 2.06)
Southern Latin America	6690 (5949 to 7524)	14.15 (12.58 to 15.88)	15,500 (13,535 to 17,755)	19.68 (17.14 to 22.58)	1.21 (1 to 1.42)	1.08 (0.31 to 1.86)
Western Europe	148,253 (140,945 to 155,877)	31.07 (29.49 to 32.71)	211,124 (193,366 to 229,245)	32.69 (29.89 to 35.55)	0.51 (0.09 to 0.94)	0.14 (−0.29 to 0.57)
Andean Latin America	2640 (2211 to 3139)	9.77 (8.12 to 11.50)	18,047 (13,837 to 22,793)	28.15 (21.63 to 35.64)	3.55 (3.34 to 3.77)	3.65 (2.75 to 4.55)
Caribbean	3118 (2874 to 3379)	10.63 (9.84 to 11.53)	9357 (8102 to 10,834)	17.80 (15.42 to 20.63)	1.86 (1.69 to 2.04)	1.84 (1.57 to 2.1)
Central Latin America	11,472 (11,050 to 11,916)	10.20 (9.83 to 10.60)	58,071 (51,803 to 65,648)	21.78 (19.43 to 24.62)	2.39 (2.28 to 2.5)	2.42 (2.21 to 2.63)
Tropical Latin America	9237 (8742 to 9744)	7.79 (7.38 to 8.20)	33,083 (31,066 to 34,984)	12.59 (11.82 to 13.32)	1.32 (1.13 to 1.51)	1.53 (1.36 to 1.71)
North Africa and Middle East	31,166 (25,920 to 41,990)	13.03 (10.80 to 17.38)	183,491 (151,632 to 216,130)	30.67 (25.45 to 36.00)	3.16 (2.99 to 3.33)	2.81 (2.65 to 2.98)
South Asia	53,580 (43,819 to 70,783)	5.95 (4.89 to 7.85)	282,509 (227,053 to 343,508)	15.33 (12.34 to 18.51)	3.24 (3.16 to 3.33)	3.07 (2.86 to 3.28)
East Asia	95,529 (76,899 to 113,230)	8.55 (6.88 to 10.10)	411,402 (334,530 to 513,557)	20.51 (16.77 to 25.61)	3.16 (3 to 3.33)	2.88 (2.75 to 3.02)
Oceania	308 (199 to 419)	6.85 (4.52 to 9.29)	960 (576 to 1382)	8.64 (5.21 to 12.33)	0.59 (0.48 to 0.7)	0.78 (0.54 to 1.02)
Southeast Asia	45,506 (36,198 to 51,840)	12.72 (10.28 to 14.50)	206,165 (161,850 to 244,211)	26.88 (21.13 to 31.79)	2.33 (2.23 to 2.43)	2.46 (2.33 to 2.6)
Central sub-Saharan Africa	870 (619 to 1302)	2.60 (1.83 to 3.89)	3432 (2195 to 5421)	3.95 (2.50 to 6.29)	1.42 (1.14 to 1.7)	1.35 (1.29 to 1.41)
Eastern sub-Saharan Africa	12,047 (9301 to 15,329)	9.39 (7.33 to 12.03)	48,137 (34,450 to 73,227)	15.43 (11.20 to 23.06)	1.47 (1.29 to 1.66)	1.62 (1.54 to 1.7)
Southern sub-Saharan Africa	2655 (2244 to 3153)	6.88 (5.76 to 8.23)	8001 (6623 to 9542)	10.67 (8.91 to 12.65)	1.8 (1.54 to 2.05)	1.5 (0.91 to 2.09)
Western sub-Saharan Africa	1674 (1213 to 2106)	1.23 (0.89 to 1.54)	6146 (4497 to 8323)	1.75 (1.30 to 2.34)	1.09 (1 to 1.18)	1.11 (1.01 to 1.22)

AAPC = average annual percentage change, ASR = age-standardized rate, EAPC = estimated annual percentage change, GBD = Global Burden of Diseases, Injuries, and Risk Factors Study, SDI = Socio-demographic Index, TC = thyroid cancer, UIs = uncertainty intervals.

**Table 3:** The case number and ASR of deaths of TC in 1990 and 2021 for both sexes by SDI quintiles and by GBD regions, with EAPC and AAPC from 1990 to 2021.

Location	1990		2021		EAPC	AAPC
	Number(95% UIs)	ASR(95% UIs)	Number(95% UIs)	ASR(95% UIs)		
Global	21,893 (20,437 to 24,108)	0.57 (0 to 53 to 0.63)	44,799 (39,925 to 48,541)	0.53 (0 to 47 to 0.57)	−0.24 (−0.27 to −0.21)	−0.23 (−0.29 to −0.17)
Gender						
Male	7362 (6917 to 8197)	0.42 (0.40 to 0.47)	18,031 (15,238 to 19,895)	0.47 (0.40 to 0.52)	0.41 (0.35 to 0.46)	0.33 (0.24 to 0.43)
Female	14,531 (13,201 to 16,693)	0.69 (0.62 to 0.78)	26,768 (23,015 to 30,905)	0.58 (0.50 to 0.68)	−0.58 (−0.61 to −0.55)	−0.51 (−0.55 to −0.46)
SDI						
High SDI	6505 (6098 to 6798)	0.59 (0.55 to 0.61)	9730 (8466 to 10,437)	0.44 (0.39 to 0.47)	−0.91 (−0.96 to −0.86)	−0.94 (−1.06 to −0.82)
High-middle SDI	5609 (5201 to 5935)	0.59 (0.54 to 0.62)	8437 (7553 to 9304)	0.43 (0.39 to 0.48)	−1.03 (−1.09 to −0.97)	−0.98 (−1.17 to −0.79)
Middle SDI	5276 (4815 to 6145)	0.53 (0.49 to 0.63)	14,667 (12,467 to 16,171)	0.57 (0.48 to 0.63)	0.21 (0.16 to 0.26)	0.2 (0.12 to 0.28)
Low-middle SDI	3015 (2624 to 3757)	0.48 (0.42 to 0.60)	8531 (7411 to 9772)	0.60 (0.52 to 0.68)	0.74 (0.71 to 0.78)	0.71 (0.57 to 0.85)
Low SDI	1455 (1199 to 1778)	0.60 (0.49 to 0.74)	3392 (2688 to 4329)	0.64 (0.52 to 0.80)	0.19 (0.11 to 0.28)	0.24 (0.17 to 0.31)
Region						
Central Asia	253 (236 to 274)	0.53 (0.50 to 0.58)	339 (302 to 379)	0.43 (0.38 to 0.48)	−0.81 (−1.43 to −0.18)	−0.83 (−2.09 to 0.43)
Central Europe	1266 (1213 to 1317)	0.86 (0.83 to 0.90)	986 (898 to 1065)	0.44 (0.40 to 0.48)	−2.37 (−2.65 to −2.1)	−2.2 (−2.6 to −1.81)
Eastern Europe	1349 (1274 to 1423)	0.50 (0.47 to 0.52)	1651 (1508 to 1807)	0.47 (0.43 to 0.52)	−0.25 (−0.6 to 0.09)	−0.26 (−0.94 to 0.42)
Australasia	98 (87 to 109)	0.42 (0.37 to 0.47)	199 (161 to 237)	0.36 (0.30 to 0.43)	0.14 (−0.13 to 0.41)	−0.54 (−0.85 to −0.23)
High-income Asia Pacific	1236 (1126 to 1404)	0.64 (0 to 58 to 0.73)	2844 (2309 to 3186)	0.50 (0.43 to 0.56)	−0.82 (−0.98 to −0.65)	−0.72 (−1.02 to −0.42)
High-income North America	1392 (1285 to 1450)	0.39 (0.36 to 0.41)	2766 (2473 to 2949)	0.41 (0.37 to 0.44)	0.15 (0.06 to 0.23)	0.18 (−0.33 to 0.7)
Southern Latin America	359 (325 to 395)	0.79 (0.71 to 0.86)	485 (426 to 554)	0.55 (0.48 to 0.63)	−1.01 (−1.26 to −0.77)	−1.16 (−1.91 to −0.41)
Western Europe	3951 (3667 to 4174)	0.68 (0.63 to 0.71)	3829 (3319 to 4191)	0.39 (0.34 to 0.42)	−1.7 (−1.77 to −1.64)	−1.8 (−1.93 to −1.68)
Andean Latin America	182 (153 to 211)	0.90 (0.76 to 1.04)	641 (508 to 794)	1.10 (0.87 to 1.36)	0.66 (0.54 to 0.77)	0.83 (0.63 to 1.03)
Caribbean	143 (132 to 156)	0.55 (0.51 to 0.60)	321 (281 to 368)	0.60 (0.52 to 0.68)	0.42 (0.2 to 0.65)	0.33 (0.1 to 0.56)
Central Latin America	636 (614 to 658)	0.79 (0.75 to 0.81)	1965 (1749 to 2165)	0.80 (0.71 to 0.88)	−0.07 (−0.26 to 0.11)	0.02 (−0.38 to 0.42)
Tropical Latin America	509 (480 to 537)	0.58 (0.54 to 0.61)	1253 (1142 to 1332)	0.50 (0.45 to 0.53)	−0.6 (−0.69 to −0.5)	−0.59 (−0.72 to −0.45)
North Africa and Middle East	658 (546 to 937)	0.40 (0.33 to 0.58)	1935 (1676 to 2236)	0.45 (0.39 to 0.52)	0.64 (0.49 to 0.79)	0.32 (0.18 to 0.47)
South Asia	2915 (2454 to 3700)	0.48 (0.41 to 0.61)	9324 (7794 to 10,741)	0.63 (0.53 to 0.73)	0.92 (0.88 to 0.97)	0.91 (0.64 to 1.17)
East Asia	3781 (3212 to 4380)	0.48 (0.41 to 0.55)	8064 (6456 to 9800)	0.39 (0.31 to 0.47)	−0.65 (−0.74 to −0.55)	−0.64 (−0.8 to −0.48)
Oceania	15 (10 to 19)	0.56 (0.41 to 0.73)	37 (23 to 50)	0.54 (0.35 to 0.73)	−0.13 (−0.16 to −0.1)	−0.12 (−0.22 to −0.02)
Southeast Asia	2008 (1698 to 2303)	0.81 (0.69 to 0.96)	5643 (4565 to 6451)	0.90 (0.74 to 1.02)	0.32 (0.24 to 0.4)	0.34 (0.27 to 0.41)
Central sub-Saharan Africa	78 (58 to 113)	0.36 (0.27 to 0.52)	183 (118 to 284)	0.35 (0.23 to 0.56)	−0.11 (−0.23 to 0.01)	−0.1 (−0.18 to −0.02)
Eastern sub-Saharan Africa	839 (686 to 1015)	1.01 (0.82 to 1.21)	1801 (1338 to 2486)	0.99 (0.74 to 1.32)	−0.18 (−0.29 to −0.07)	−0.05 (−0.1 to 0)
Southern sub-Saharan Africa	124 (102 to 148)	0.45 (0.37 to 0.55)	324 (264 to 372)	0.57 (0.46 to 0.65)	0.92 (0.65 to 1.18)	0.82 (0.61 to 1.02)
Western sub-Saharan Africa	103 (78 to 123)	0.11 (0.08 to 0.13)	210 (165 to 264)	0.09 (0.08 to 0.12)	−0.51 (−0.59 to −0.43)	−0.4 (−0.49 to −0.31)

AAPC = average annual percentage change, ASR = age-standardized rate, EAPC = estimated annual percentage change, GBD = Global Burden of Diseases, Injuries, and Risk Factors Study, SDI = Socio-demographic Index, TC = thyroid cancer, UIs = uncertainty intervals.

**Table 4:** The case number and ASR of DALYs of TC in 1990 and 2021 for both sexes by SDI quintiles and by GBD regions, with EAPC and AAPC from 1990 to 2021.

Location	1990		2021		EAPC	AAPC
	Number(95% UIs)	ASR(95% UIs)	Number(95% UIs)	ASR(95% UIs)		
Global	646,741 (599,119 to 717,357)	15.21 (14.18 to 16.83)	1,246,485 (1,094,416 to 1,375,853)	14.57 (12.78 to 16.11)	−0.14 (−0.17 to −0.11)	−0.13 (−0.24 to −0.02)
Gender						
Male	225,602 (210,319 to 250,806)	11.19 (10.45 to 12.46)	504,440 (421,688 to 563,175)	11.19 (10.45 to 12.46)	0.41 (0.35 to 0.46)	0.31 (0.2 to 0.42)
Female	421,139 (375,418 to 489,860)	18.82 (16.87 to 21.84)	742,044 (627,065 to 865,021)	18.82 (16.87 to 21.84)	−0.45 (−0.47 to −0.42)	−0.39 (−0.43 to −0.35)
SDI						
High SDI	164,382 (155,871 to 173,718)	15.38 (14.59 to 16.28)	220,136 (201,450 to 237,835)	11.76 (10.79 to 12.77)	−0.77 (−0.86 to −0.68)	−0.88 (−1.04 to −0.72)
High-middle SDI	158,942 (146,717 to 170,153)	15.57 (14.40 to 16.45)	218,961 (196,635 to 244,418)	11.63 (10.44 to 13.02)	−0.99 (−1.05 to −0.92)	−0.96 (−1.18 to −0.74)
Middle SDI	166,177 (149,966 to 190,691)	14.02 (12.76 to 16.18)	416,218 (350,751 to 460,889)	15.22 (12.81 to 16.81)	0.28 (0.22 to 0.34)	0.27 (0.2 to 0.34)
Low-middle SDI	103,648 (90,343 to 129,887)	13.75 (11.99 to 17.15)	269,914 (229,089 to 316,826)	16.75 (14.35 to 19.42)	0.67 (0.63 to 0.7)	0.65 (0.55 to 0.75)
Low SDI	52,662 (43,472 to 63,948)	17.75 (14.57 to 21.62)	120,143 (93,673 to 157,131)	17.98 (14.18 to 23.06)	−0.05 (−0.12 to 0.02)	0.04 (−0.03 to 0.12)
Region						
Central Asia	8145 (7568 to 8817)	15.87 (14.74 to 17.22)	10,273 (9056 to 11,543)	11.66 (10.33 to 13.03)	−1.19 (−1.81 to −0.57)	−1.12 (−2.44 to 0.23)
Central Europe	34,786 (33,224 to 36,474)	23.49 (22.42 to 24.62)	23,434 (21,250 to 25,475)	11.60 (10.50 to 12.65)	−2.47 (−2.76 to −2.17)	−2.35 (−2.66 to −2.03)
Eastern Europe	37,930 (35,923 to 40,329)	13.86 (13.11 to 14.73)	42,086 (38,218 to 46,388)	12.87 (11.66 to 14.15)	−0.29 (−0.68 to 0.11)	−0.37 (−1.2 to 0.46)
Australasia	2544 (2273 to 2868)	11.05 (9.87 to 12.46)	5041 (4104 to 6033)	10.61 (8.67 to 12.78)	0.51 (0.21 to 0.82)	−0.23 (−0.56 to 0.11)
High-income Asia Pacific	30,856 (28,505 to 35,131)	15.36 (14.15 to 17.48)	50,783 (43,704 to 57,571)	11.81 (10.45 to 13.68)	−0.75 (−0.99 to −0.5)	−0.83 (−1.18 to −0.49)
High-income North America	37,316 (34,968 to 39,721)	11.23 (10.51 to 11.97)	70,642 (65,004 to 76,457)	11.96 (11.04 to 13.03)	0.21 (0.11 to 0.31)	0.21 (−0.43 to 0.85)
Southern Latin America	9492 (8618 to 10,470)	20.25 (18.38 to 22.34)	11,960 (10,465 to 13,594)	14.26 (12.48 to 16.21)	−1.02 (−1.28 to −0.75)	−0.96 (−1.62 to −0.3)
Western Europe	94,707 (89,027 to 100,543)	17.60 (16.60 to 18.76)	84,465 (75,823 to 92,550)	10.46 (9.44 to 11.54)	−1.5 (−1.64 to −1.36)	−1.69 (−1.81 to −1.56)
Andean Latin America	5274 (4419 to 6186)	23.02 (19.33 to 26.97)	16,690 (13,208 to 20,767)	27.53 (21.72 to 34.25)	0.54 (0.43 to 0.65)	0.69 (0.47 to 0.91)
Caribbean	4117 (3769 to 4556)	14.92 (13.67 to 16.46)	8690 (7533 to 10,099)	16.34 (14.15 to 18.98)	0.46 (0.25 to 0.68)	0.4 (0.19 to 0.62)
Central Latin America	18,293 (17,666 to 18,921)	19.54 (18.87 to 20.23)	52,187 (47,013 to 58,083)	20.39 (18.36 to 22.67)	0 (−0.2 to 0.2)	0.11 (−0.23 to 0.46)
Tropical Latin America	15,021 (14,259 to 15,823)	14.78 (14.02 to 15.58)	32,680 (30,547 to 34,741)	12.68 (11.83 to 13.49)	−0.65 (−0.75 to −0.55)	−0.59 (−0.72 to −0.45)
North Africa and Middle East	21,944 (18,189 to 30,746)	10.99 (9.07 to 15.49)	65,316 (55,454 to 76,496)	12.68 (10.87 to 14.80)	0.76 (0.62 to 0.9)	0.47 (0.37 to 0.56)
South Asia	105,303 (88,400 to 135,793)	14.21 (11.98 to 18.18)	302,257 (249,828 to 356,789)	18.22 (15.09 to 21.35)	0.85 (0.81 to 0.88)	0.83 (0.67 to 0.99)
East Asia	116,582 (98,062 to 136,721)	12.25 (10.38 to 14.20)	213,609 (173,066 to 262,362)	10.25 (8.34 to 12.52)	−0.56 (−0.67 to −0.46)	−0.58 (−0.75 to −0.41)
Oceania	480 (325 to 641)	13.90 (9.85 to 18.46)	1174 (725 to 1642)	13.55 (8.61 to 18.63)	−0.1 (−0.14 to −0.06)	−0.09 (−0.25 to 0.06)
Southeast Asia	62,695 (51,465 to 70,375)	21.41 (17.91 to 24.29)	164,547 (130,333 to 189,174)	23.60 (18.80 to 27.01)	0.27 (0.21 to 0.34)	0.32 (0.24 to 0.39)
Central sub-Saharan Africa	2545 (1889 to 3610)	9.44 (6.94 to 13.66)	5956 (3863 to 9188)	8.92 (5.75 to 13.93)	−0.19 (−0.31 to −0.06)	−0.18 (−0.27 to −0.09)
Eastern sub-Saharan Africa	31,046 (25,044 to 38,047)	30.05 (24.54 to 36.69)	66,491 (48,520 to 94,804)	27.76 (20.55 to 38.36)	−0.43 (−0.55 to −0.32)	−0.26 (−0.3 to −0.21)
Southern sub-Saharan Africa	4035 (3399 to 4776)	12.40 (10.31 to 14.79)	10,253 (8481 to 11,926)	15.67 (12.84 to 18.14)	1 (0.72 to 1.27)	0.75 (0.55 to 0.94)
Western sub-Saharan Africa	3629 (2762 to 4379)	3.18 (2.41 to 3.82)	7951 (6109 to 10,289)	2.82 (2.21 to 3.55)	−0.49 (−0.56 to −0.42)	−0.39 (−0.51 to −0.27)

AAPC = average annual percentage change, ASR = age-standardized rate, DALYs = disability-adjusted life-years, EAPC = estimated annual percentage change, GBD = Global Burden of Diseases, Injuries, and Risk Factors Study, SDI = Socio-demographic Index, TC = thyroid cancer, UIs = uncertainty intervals.

Regionally, in 2021, East Asia surpassed Western Europe, which held the highest number of TC cases in 1990, becoming the region with the highest incidence and prevalence. South Asia replaced Western Europe as the region with the highest number of deaths due to TC, while South Asia also surpassed East Asia in terms of the highest number of DALY. In 2021, high-income North America exhibited the highest ASR for incidence and prevalence, while the Andean Latin America region had the highest ASR of deaths, and Eastern Sub-Saharan Africa showed the highest DALY ASR (Tables 1–4).

**Figure 1.**
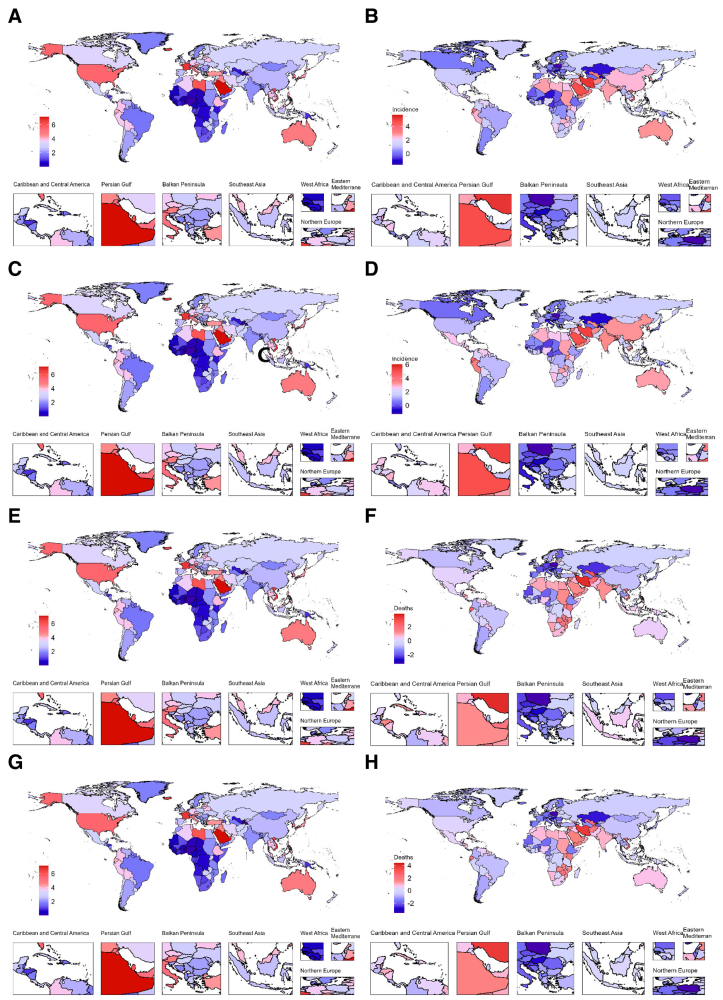
(A) The ASR of incidence in 2019; (B) the trend in ASR of incidence (EAPC) from 1990 to 2019; (C) the ASR of prevalence in 2019; (D) the trend in ASR of prevalence (EAPC) from 1990 to 2019; (E) the ASR of death in 2019; (F) the trend in ASR of deaths (EAPC) from 1990 to 2019; (G) the ASR of DALYs in 2019; and (H) the trend in ASR of DALYs (EAPC) from 1990 to 2019; of TC worldwide. ASR = age-standardized rate, EAPC = estimated annual percentage change, DALYs = disability-adjusted life-years, TC = thyroid cancer.

From the perspective of SDI quintiles, overall, higher SDI regions exhibited higher ASR for incidence and prevalence of TC, while lower SDI regions had higher deaths and DALY ASR. High SDI regions showed the highest incidence and prevalence rates, whereas low SDI regions demonstrated the highest age-standardized DALY rates (Tables 1–3). Additionally, at the national level, there were significant differences in the global incidence, prevalence, deaths, and DALY of TC. China had the highest number of cases for incidence, prevalence, and deaths, while India recorded the highest DALY value. France had the highest ASR for incidence and prevalence, Japan had the highest ASR of deaths, and Georgia had the highest DALY ASR (Fig. 1; Tables S1–S4, Supplemental Digital Content 1).

### 3.2. Overall trends of TC burden using joint point regression analysis

**Figure 2.**
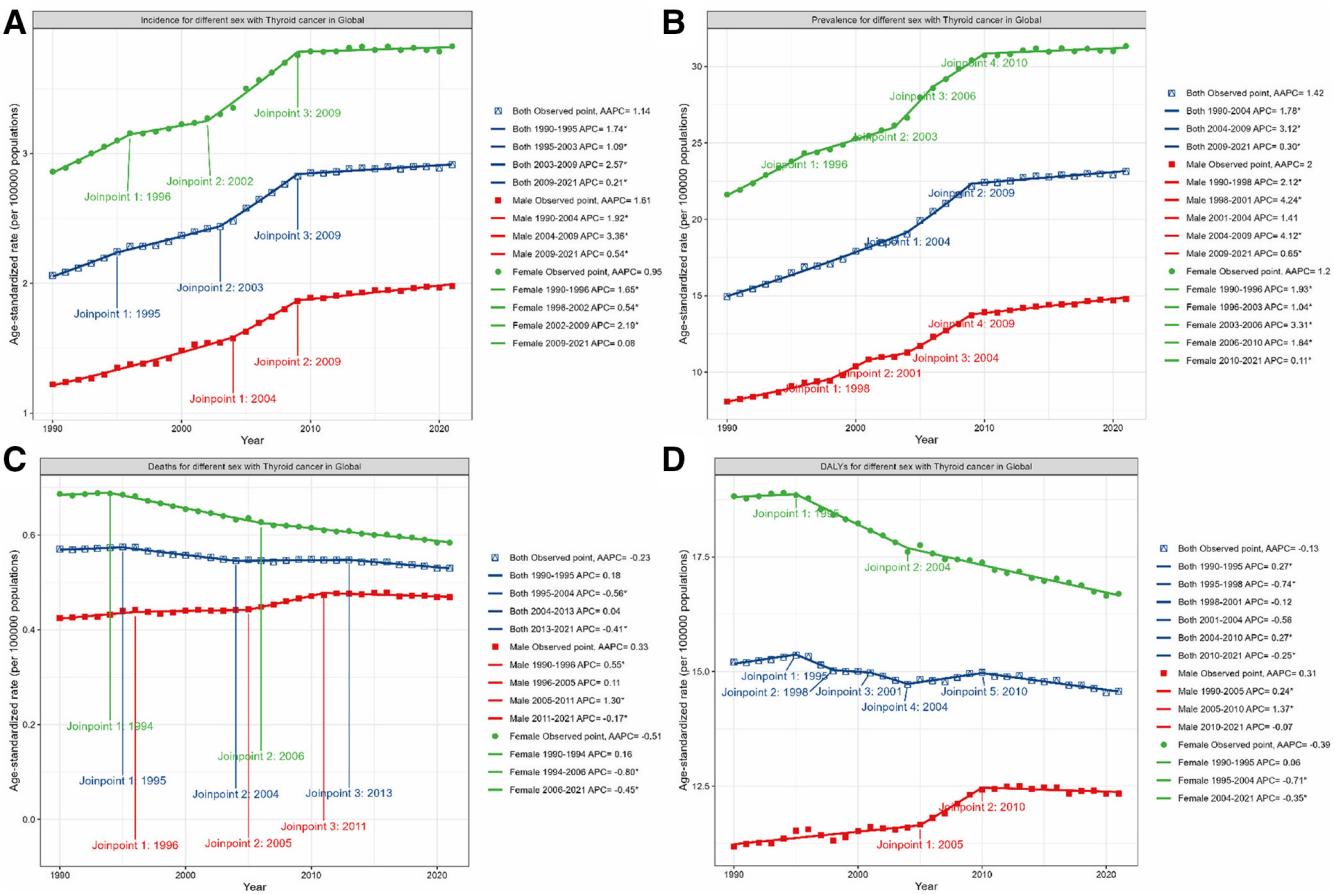
(A) The joinpoint regression analysis on the ASR of incidence between different genders; (B) the joinpoint regression analysis on the ASR of prevalence between different genders; (C) the joinpoint regression analysis on the ASR of deaths between different genders; and (D) the joinpoint regression analysis on the ASR of DALYs between different genders; of TC globally. ASR = age-standardized rate, DALYs = disability-adjusted life-years, TC = thyroid cancer.

The results of the joint point regression analysis for the burden of TC are illustrated in Figure 2. From 1990 to 2021, the ASR of TC incidence showed an increasing trend at various connection points, although the rate of increase varied between these points. Notably, the growth rate was fastest during the period from 2003 to 2009, while the slowest growth occurred from 1995 to 2003. Similarly, the trend of the ASR for prevalence exhibited an overall upward trajectory, experiencing 2 turning points in 2004 and 2009, with the fastest growth occurring from 2004 to 2009.

In contrast, the ASR for TC mortality exhibited relatively stable rates overall, consistently maintaining between 0.5 and 0.6 per 100,000 population. The trend of the ASR for DALY experienced 5 turning points, showing slight increases during the periods of 1990 to 1995 and 2004 to 2010, and slight decreases during the periods of 1995 to 2004 and 2010 to 2021. From 1995 to 2004, there was a decline at varying rates, ultimately demonstrating a decreasing trend from 2010 to 2021.

### 3.3. Age-period-cohort analysis of TC incidence, prevalence, and deaths

**Figure 3.**
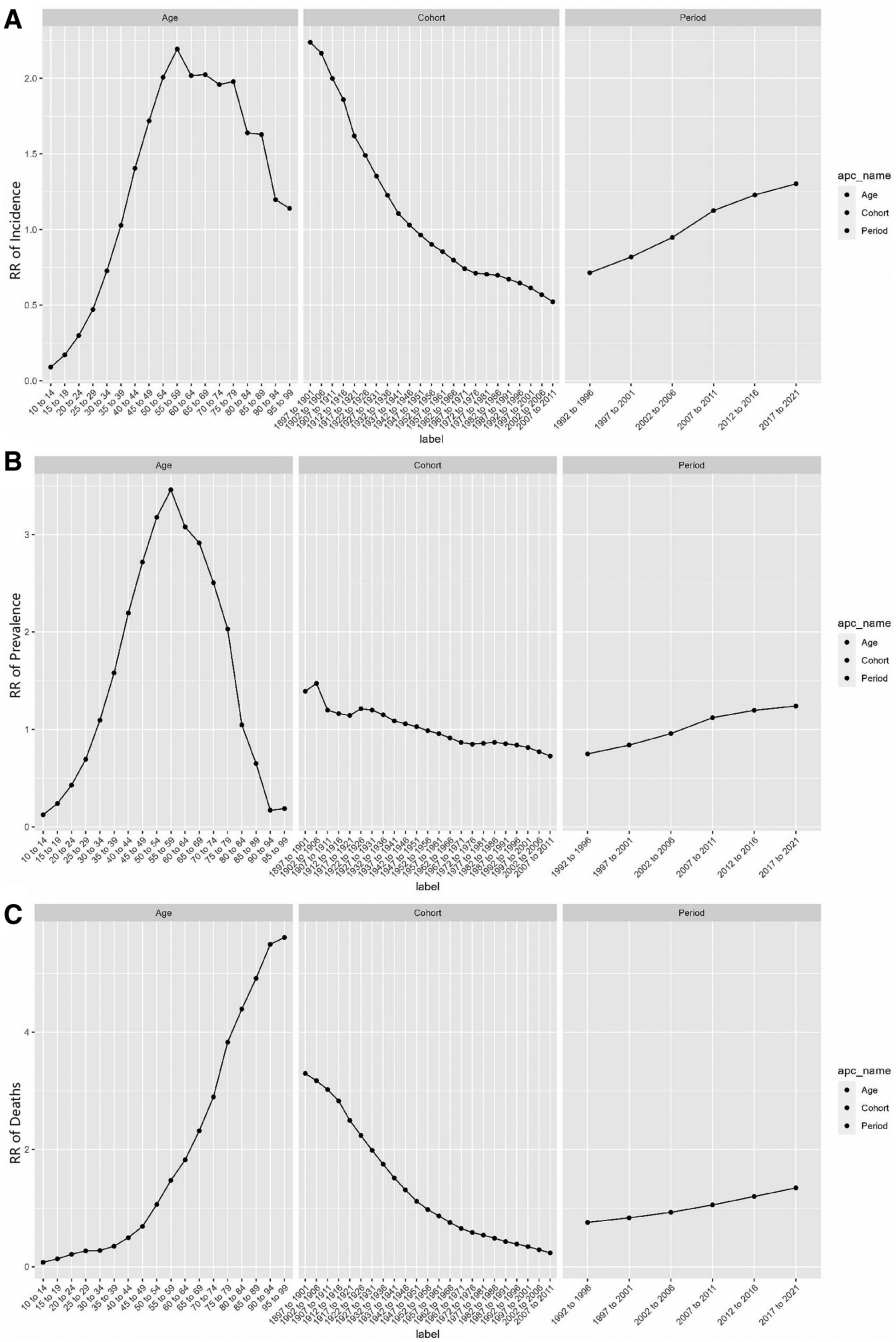
The effects of age, period, and birth cohort on the relative risk of TC incidence (A), prevalence (B), and deaths (C). TC = thyroid cancer.

The results of the age-period-cohort analysis for TC incidence and prevalence and deaths are presented in Figure 3. After controlling for period and birth cohort effects, the age effect significantly impacted the risk of TC incidence, prevalence, and deaths. Both incidence and prevalence exhibited a trend of increasing risk followed by a decrease, with the highest risk observed in the age group of 55 to 59 years (Table 5). Conversely, the age effect on the risk of TC mortality showed an increasing trend, with the highest mortality rate observed in the age group of 95 to 99 years.

**Table 5:** RRs of TC incidence, prevalence and deaths for both sexes due to age, period, and birth cohort effects.

Factor	Incidence		Prevalence		Deaths	
	RR (95% CI)	*P*	RR (95% CI)	*P*	RR (95% CI)	*P*
Ages						
5 to 9	0.065 (0.063–0.067)	<.001	0.087 (0.085–0.089)	<.001	0.057 (0.051–0.064)	<.001
10 to 14	0.117 (0.114–0.120)	<.001	0.160 (0.157–0.163)	<.001	0.095 (0.088–0.102)	<.001
15 to 19	0.218 (0.214–0.223)	<.001	0.304 (0.299–0.310)	<.001	0.166 (0.157–0.176)	<.001
20 to 24	0.376 (0.369–0.382)	<.001	0.534 (0.526–0.542)	<.001	0.261 (0.249–0.273)	<.001
25 to 29	0.584 (0.576–0.593)	<.001	0.853 (0.842–0.864)	<.001	0.328 (0.315–0.342)	<.001
30 to 34	0.889 (0.878–0.901)	<.001	1.326 (1.313–1.340)	<.001	0.334 (0.321–0.348)	<.001
35 to 39	1.245 (1.232–1.258)	<.001	1.892 (1.877–1.907)	<.001	0.422 (0.407–0.437)	<.001
40 to 44	1.681 (1.665–1.698)	<.001	2.593 (2.576–2.609)	<.001	0.592 (0.574–0.611)	<.001
45 to 49	2.033 (2.015–2.051)	<.001	3.169 (3.152–3.186)	<.001	0.813 (0.791–0.837)	<.001
50 to 54	2.347 (2.327–2.368)	<.001	3.659 (3.638–3.679)	<.001	1.248 (1.218–1.279)	<.001
55 to 59	2.536 (2.513–2.559)	<.001	3.932 (3.905–3.959)	<.001	1.721 (1.685–1.758)	<.001
60 to 64	2.306 (2.283–2.329)	<.001	3.455 (3.425–3.485)	<.001	2.117 (2.077–2.159)	<.001
65 to 69	2.288 (2.262–2.314)	<.001	3.227 (3.192–3.262)	<.001	2.678 (2.631–2.726)	<.001
70 to 74	2.188 (2.161–2.216)	<.001	2.740 (2.704–2.777)	<.001	3.326 (3.269–3.384)	<.001
75 to 79	2.185 (2.154–2.217)	<.001	2.193 (2.159–2.228)	<.001	4.374 (4.297–4.452)	<.001
80 to 84	1.790 (1.759–1.822)	<.001	1.115 (1.094–1.136)	<.001	4.990 (4.892–5.090)	<.001
85 to 89	1.759 (1.722–1.796)	<.001	0.685 (0.669–0.701)	<.001	5.553 (5.423–5.686)	<.001
90 to 94	1.279 (1.240–1.319)	<.001	0.178 (0.170–0.185)	<.001	6.173 (5.986–6.366)	<.001
95 to 99	1.203 (1.142–1.268)	<.001	0.192 (0.179–0.206)	<.001	6.271 (5.977–6.579)	<.001
Period						
1992 to 1996	0.695 (0.690–0.700)	<.001	0.726 (0.721–0.731)	<.001	0.747 (0.737–0.758)	<.001
1997 to 2001	0.805 (0.800–0.810)	<.001	0.823 (0.820–0.827)	<.001	0.829 (0.819–0.839)	<.001
2002 to 2006	0.942 (0.938–0.947)	<.001	0.950 (0.948–0.952)	<.001	0.926 (0.916–0.936)	<.001
2007 to 2011	1.132 (1.127–1.137)	<.001	1.127 (1.125–1.129)	<.001	1.058 (1.047–1.069)	<.001
2012 to 2016	1.250 (1.244–1.256)	<.001	1.220 (1.215–1.225)	<.001	1.209 (1.197–1.222)	<.001
2017 to 2021	1.341 (1.333–1.349)	<.001	1.281 (1.273–1.289)	<.001	1.364 (1.348–1.380)	<.001
Birth cohort						
1897 to 1901	2.642 (2.159–3.232)	<.001	1.646 (1.249–2.169)	<.001	3.769 (3.184–4.462)	<.001
1902 to 1906	2.528 (2.317–2.759)	<.001	1.720 (1.525–1.940)	<.001	3.608 (3.339–3.898)	<.001
1907 to 1911	2.308 (2.199–2.422)	<.001	1.381 (1.307–1.459)	<.001	3.419 (3.258–3.589)	<.001
1912 to 1916	2.122 (2.047–2.200)	<.001	1.323 (1.270–1.378)	<.001	3.180 (3.065–3.300)	<.001
1917 to 1921	1.827 (1.772–1.883)	<.001	1.286 (1.241–1.332)	<.001	2.792 (2.706–2.881)	<.001
1922 to 1926	1.663 (1.620–1.708)	<.001	1.346 (1.304–1.390)	<.001	2.490 (2.422–2.559)	<.001
1927 to 1931	1.493 (1.458–1.529)	<.001	1.313 (1.276–1.352)	<.001	2.197 (2.142–2.254)	<.001
1932 to 1936	1.338 (1.310–1.368)	<.001	1.245 (1.212–1.278)	<.001	1.924 (1.877–1.972)	<.001
1937 to 1941	1.193 (1.170–1.217)	<.001	1.162 (1.134–1.190)	<.001	1.657 (1.617–1.699)	<.001
1942 to 1946	1.098 (1.079–1.119)	<.001	1.117 (1.093–1.141)	<.001	1.428 (1.392–1.465)	<.001
1947 to 1951	1.016 (1.000–1.033)	.056	1.071 (1.051–1.092)	<.001	1.209 (1.177–1.243)	<.001
1952 to 1956	0.940 (0.926–0.954)	<.001	1.015 (0.999–1.032)	.075	1.052 (1.022–1.083)	.001
1957 to 1961	0.880 (0.868–0.892)	<.001	0.971 (0.957–0.984)	<.001	0.928 (0.899–0.957)	<.001
1962 to 1966	0.812 (0.802–0.823)	<.001	0.914 (0.904–0.925)	<.001	0.806 (0.779–0.834)	<.001
1967 to 1971	0.746 (0.737–0.756)	<.001	0.857 (0.849–0.865)	<.001	0.693 (0.667–0.719)	<.001
1972 to 1976	0.708 (0.700–0.717)	<.001	0.829 (0.823–0.835)	<.001	0.616 (0.591–0.641)	<.001
1977 to 1981	0.693 (0.685–0.702)	<.001	0.827 (0.823–0.831)	<.001	0.564 (0.540–0.590)	<.001
1982 to 1986	0.678 (0.669–0.687)	<.001	0.825 (0.821–0.829)	<.001	0.508 (0.485–0.533)	<.001
1987 to 1991	0.648 (0.639–0.657)	<.001	0.805 (0.800–0.809)	<.001	0.448 (0.425–0.471)	<.001
1992 to 1996	0.613 (0.603–0.624)	<.001	0.777 (0.771–0.783)	<.001	0.399 (0.377–0.423)	<.001
1997 to 2001	0.571 (0.559–0.583)	<.001	0.738 (0.731–0.745)	<.001	0.350 (0.326–0.376)	<.001
2002 to 2006	0.518 (0.503–0.533)	<.001	0.683 (0.674–0.693)	<.001	0.294 (0.267–0.325)	<.001
2007 to 2011	0.471 (0.452–0.492)	<.001	0.635 (0.624–0.647)	<.001	0.240 (0.208–0.278)	<.001
2012 to 2016	0.409 (0.377–0.442)	<.001	0.562 (0.545–0.579)	<.001	0.189 (0.143–0.249)	<.001

CI = confidence interval, RRs = relative risk, TC = thyroid cancer.

After controlling for age and birth cohort effects, significant period effects were observed on the risk of TC incidence, prevalence, and deaths. The period effects for incidence, prevalence, and mortality risks demonstrated an increasing trend, with RRs increasing by 1.93, 1.76, and 1.83 times, respectively, from the age period of 1992 to 1996 to that of 2017 to 2021. The highest risks for incidence, prevalence, and deaths were observed during the age period of 2017 to 2021 (Table 5).

After controlling for age and period effects, significant birth cohort effects were noted on the incidence, prevalence, and deaths rates of TC. The birth cohort effect indicated that earlier birth cohorts had higher risks of incidence, prevalence, and deaths compared to later birth cohorts. Specifically, the RRs for incidence and deaths continuously declined from the cohort born in 1897 to 1901 to the cohort born in 2012 to 2016 (Table 5), while the highest prevalence RR was observed among individuals born in the cohort of 1902 to 1906.

### 3.4. Decomposition analysis of TC DALY

**Figure 4.**
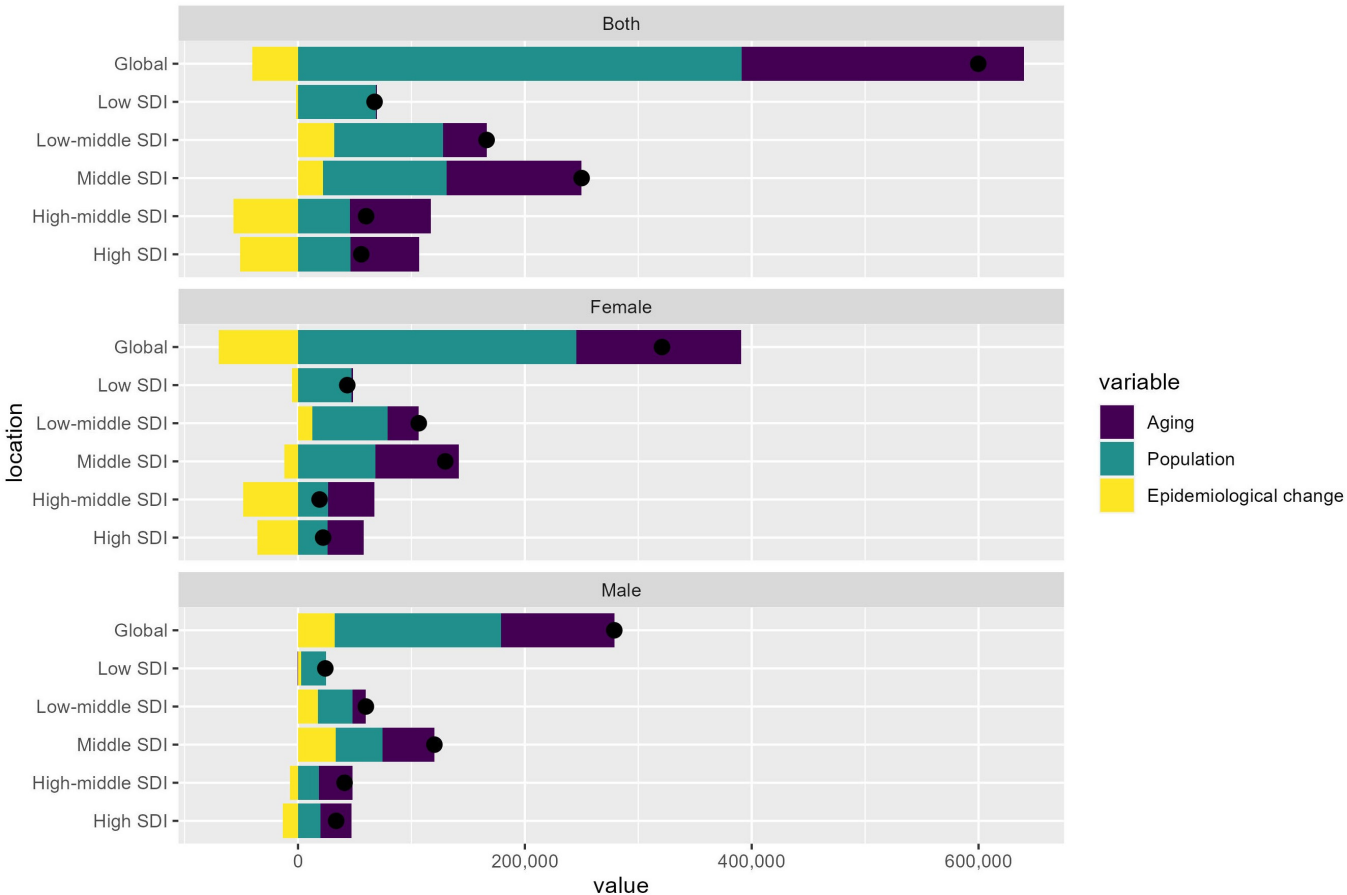
Changes in DALYs of TC according to aging, population growth and epidemiological change from 1990 to 2021 at global level by SDI quintile and sexes. The black dot denotes the overall value of the change resulting from all 3 components. For each component, the magnitude of a positive value suggests a corresponding increase in TC DALYs attributed to the component; the magnitude of a negative value suggests a corresponding decrease in TC DALYs attributed to the component. DALYs = disability-adjusted life-years, SDI = Socio-demographic Index, TC = thyroid cancer.

Over the past 30 years, the global DALY associated with TC have increased, with the highest growth observed in regions with a medium Socio-Demographic Index (SDI; Fig. 4). The contributions of aging, population growth, and epidemiological changes to the increase in global DALY were 41.46%, 65.23%, and −6.70%, respectively. The most significant contributions from aging, population growth, and epidemiological changes occurred in the High-middle SDI region (118.61%), Low SDI region (102.07%), and Low-middle SDI region (19.24%), respectively (Table 6). In terms of age, the DALY for the female population accounted for 53.51% of the total DALY for both sexes, which is higher than the 46.49% for males. Additionally, it is noteworthy that the contribution of population growth to the increase in DALY was the largest. These results indicate that population growth is a significant influencing factor in the development of the disease. Furthermore, when stratified by gender, females consistently exhibited a higher burden than males across all SDI regions.

**Table 6:** The impact of aging, population growth, and epidemiological change on DALYs across global and different SDI regions.

	Location	Overall difference	Aging	Population growth	Epidemiological change
DALYs	Global	599,744.26	248,668.98 (41.46%)	391,235.8 (65.23%)	−40,160.52 (−6.70%)
	High SDI	55,754.37	60,483.78 (108.48%)	46,421.96 (83.26%)	−51,151.37 (−91.74%)
	High-middle SDI	60,019.14	71,189.64 (118.61%)	45,720.67 (76.18%)	−56,891.17 (−94.79%)
	Middle SDI	250,040.64	118,987.51 (47.59%)	108,844.14 (43.53%)	22,208.99 (8.88%)
	Low-middle SDI	166,266.41	38,286.45 (23.03%)	95,993.38 (57.73%)	31,986.58 (19.24%)
	Low SDI	67,481.79	533.03 (0.79%)	68,878.21 (102.07%)	−1929.46 (−2.86%)

DALYs = disability-adjusted life-years, SDI = Socio-demographic Index.

### 3.5. Cross-country inequality analysis

**Figure 5.**
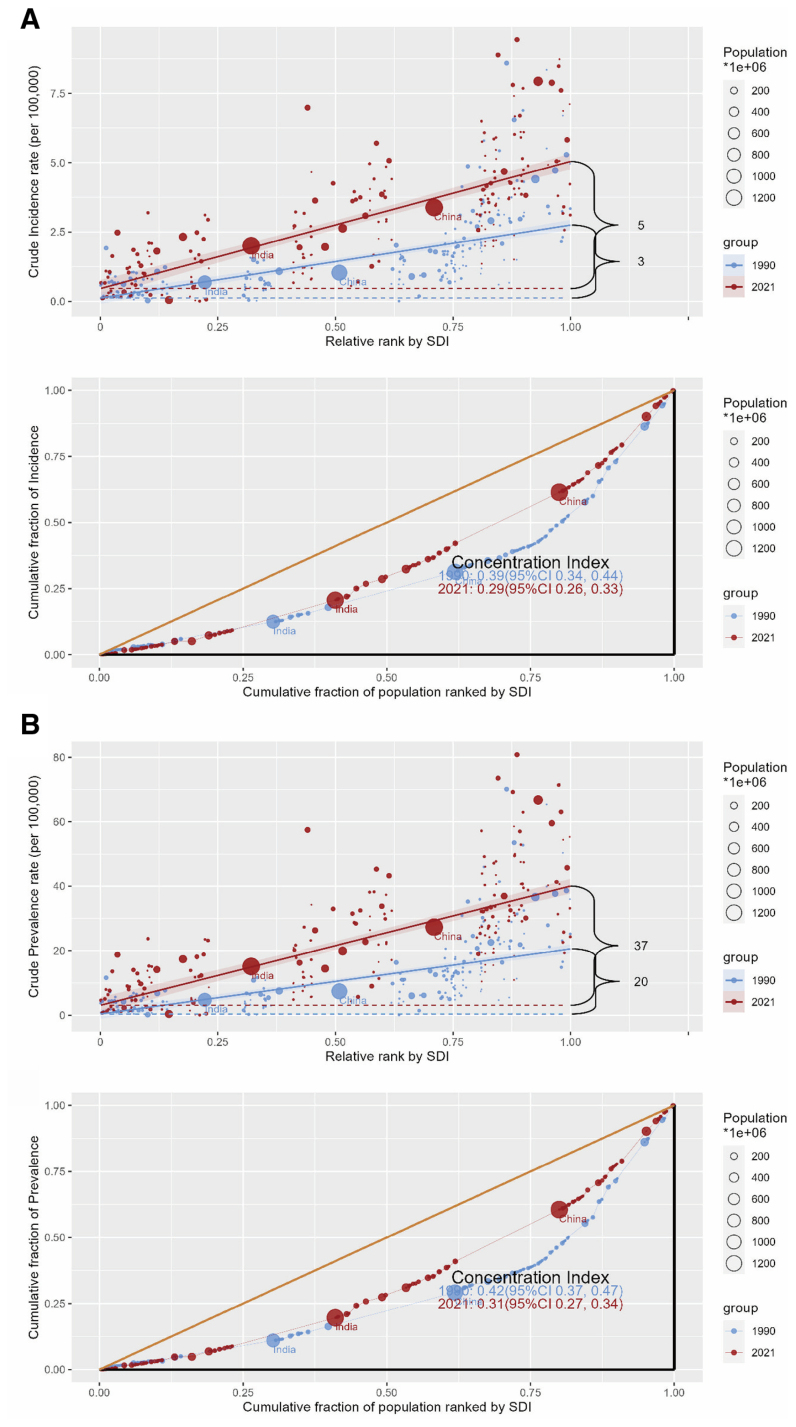
SDI-related health inequality regression and concentration curves for the incidence (A) and prevalence (B) of TC worldwide, 1990 and 2021. SDI = Socio-demographic Index, TC = thyroid cancer.

Significant absolute and relative inequalities related to the burden of TC associated with the SDI were observed, with these inequalities increasing significantly over time (Fig. 5, Table 7). Interestingly, the incidence and prevalence were concentrated in countries with higher levels of socio-demographic development. As indicated by the inequality slope index, the incidence gap between the highest and lowest SDI countries increased from 2.63 (95% CI: 2.28 to 2.98) in 1990 to 4.58 (95% CI: 4.04 to 5.13) in 2021. For prevalence, the gap between the highest and lowest SDI countries increased from 20.17 (95% CI: 17.31 to 23.02) in 1990 to 36.96 (95% CI: 32.26 to 41.65) in 2021. Both the incidence and prevalence exhibited a positive slope index at both time points, with absolute values increasing, indicating a positive correlation between incidence and prevalence with SDI levels and that this inter-regional inequality has intensified.

**Table 7:** Values and their 95% CI of the inequality slope index and concentration index of incidence and prevalence for 1990 and 2021.

Measure	Health inequality metrics	Year	Value	95% CI
Incidence	Slope index of inequality	1990	2.63	2.28 to 2.98
		2021	4.58	4.04 to 5.13
	Concentration index	1990	0.39	0.34 to 0.44
		2021	0.29	0.26 to 0.33
Prevalence	Slope index of inequality	1990	20.17	17.31 to 23.02
		2021	36.96	32.26 to 41.65
	Concentration index	1990	0.42	0.37 to 0.47
		2021	0.31	0.27 to 0.34

CI = confidence interval.

Furthermore, the concentration index for TC incidence (a measure of relative gradient inequality) was 0.39 (95% CI: 0.34 to 0.44) in 1990 and 0.29 (95% CI: 0.26 to 0.33) in 2021. The concentration index for TC prevalence was 0.42 (95% CI: 0.37 to 0.47) in 1990 and 0.31 (95% CI: 0.27 to 0.34) in 2021, indicating a decrease in the degree of concentration for both incidence and prevalence of TC. Overall, these 2 indicators suggest an imbalanced distribution of the burden between countries with different SDI levels, reflecting a complex pattern of changing inequalities.

### 3.6. Predictive analysis of TC burden by 2045

**Figure 6.**
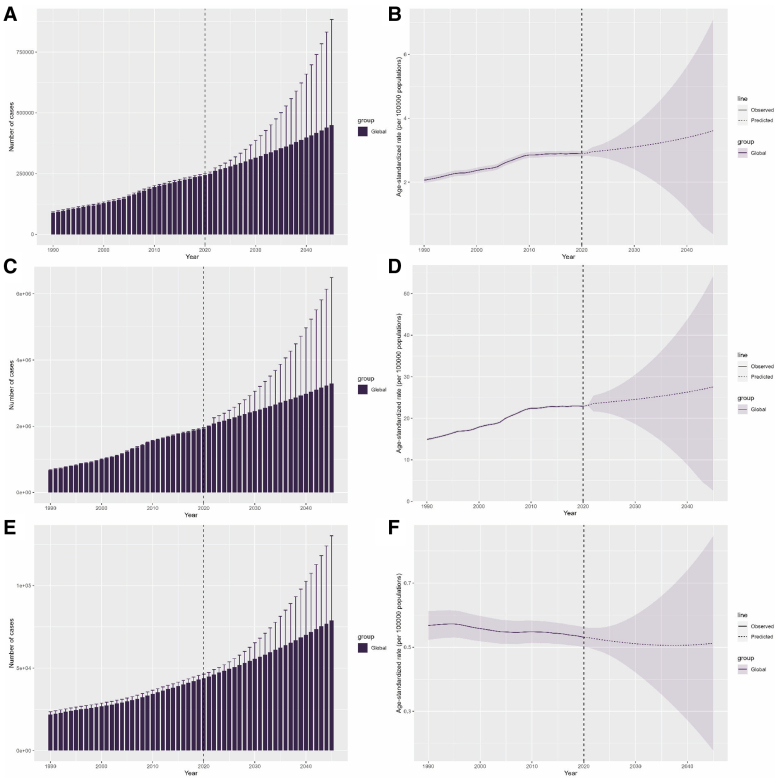
(A) The predicted case number of incidence to 2045; (B) the predicted ASR of incidence to 2045; (C) the predicted case number of prevalence to 2045; (D) the predicted ASR of prevalence to 2045; (E) the predicted case number of deaths to 2045; and (F) the predicted ASR of deaths to 2045; of TC globally. ASR = age-standardized rate, TC = thyroid cancer.

The predicted numbers of cases and ASR for incidence, prevalence, and deaths of TC by 2045 are illustrated in Figure 6. From 2021 to 2045, it is anticipated that the number of cases and ASRs for both incidence and prevalence will increase annually on a global scale. By 2045, the incidence is expected to reach approximately 449,910 cases, which is about 1.80 times that of 2021; the prevalence is projected to reach around 3295,386 cases, approximately 1.66 times that of 2021; and the mortality is expected to reach about 78,818 cases, roughly 1.76 times that of 2021. We anticipate that the number of deaths due to TC will increase annually, while the ASR of deaths is expected to show a declining trend. Table 8 provides detailed values for the number of cases and ASRs for incidence, prevalence, and deaths.

**Table 8:** Detailed values for the number of cases and ASRs for incidence, prevalence, and deaths.

Year	Num.pred_val	Num.pred_low	Num.pred_up	ASR.pred_val	ASR.pred_low	ASR.pred_up
Incidence						
2021	249,422.6446	242,451.6888	256,393.6004	2.911413497	2.828513366	2.994313629
2022	260,777.4848	248,069.0766	273,485.893	2.951373065	2.806634173	3.096111957
2023	267,117.3413	251,724.8421	282,509.8405	2.967843219	2.79599748	3.139688957
2024	273,600.4774	253,979.3083	293,221.6465	2.984940159	2.770179155	3.199701163
2025	280,197.1953	254,934.3929	305,459.9976	3.002608202	2.731314979	3.273901426
2026	286,885.1788	254,707.8279	319,062.5298	3.020854182	2.681556748	3.360151617
2027	293,671.728	253,390.4819	333,952.974	3.040046351	2.622669669	3.457423032
2028	300,609.3132	251,095.8392	350,122.7872	3.060438464	2.556042266	3.564834662
2029	307,711.1	247,854.8634	367,567.3366	3.081792255	2.482089839	3.68149467
2030	314,950.1247	243,634.1025	386,266.147	3.103993826	2.400985764	3.807001889
2031	322,314.2844	238,387.044	406,241.5248	3.126994887	2.312679982	3.941309792
2032	329,829.677	232,079.6092	427,579.7449	3.151185233	2.217276871	4.085093595
2033	337,557.5919	224,716.5002	450,398.6835	3.176953476	2.115006023	4.238900929
2034	345,511.2061	216,243.9165	474,778.4957	3.204100069	2.005480716	4.402719421
2035	353,655.372	206,559.2775	500,751.4666	3.232411268	1.88816749	4.576655046
2036	361,965.7375	195,549.022	528,382.4531	3.261705532	1.762392791	4.761018274
2037	370,475.6516	183,116.265	557,835.0383	3.292353183	1.627664997	4.957041368
2038	379,287.1003	169,203.2824	589,370.9182	3.325018963	1.483725037	5.166312888
2039	388,435.6214	153,683.6332	623,187.6097	3.359660186	1.329718274	5.389602099
2040	397,872.9881	136,370.5951	659,375.3811	3.396042851	1.164528272	5.62755743
2041	407,557.3212	117,069.4293	698,059.3176	3.433917296	0.987039166	5.881011889
2042	417,521.0732	95,594.4814	739,513.9212	3.473676045	0.796274401	6.152067697
2043	427,872.0357	74,947.52693	784,155.719	3.516332644	0.617256795	6.443963315
2044	438,674.0081	61,004.70016	832,387.7849	3.562034094	0.495819137	6.758845079
2045	449,910.071	45,706.07756	884,520.2088	3.61050094	0.366432781	7.09842304
Prevalence						
2021	1,987,127.635	1,966,992.752	2,007,262.518	23.1421516	22.90334381	23.38095939
2022	2,084,939.355	1,913,338.773	2,256,539.937	23.56442971	21.6237493	25.50511011
2023	2,130,738.817	1,940,599.3	2,320,878.335	23.6777122	21.56353305	25.79189134
2024	2,176,997.66	1,958,275.691	2,395,719.629	23.79508976	21.40315051	26.18702901
2025	2,223,561.777	1,965,741.271	2,481,382.283	23.91511077	21.14092915	26.6892924
2026	2,270,188.888	1,963,063.835	2,577,313.94	24.03557749	20.78265303	27.28850196
2027	2,317,033.46	1,950,921.365	2,683,145.555	24.15887537	20.3402756	27.97747514
2028	2,364,365.097	1,930,141.929	2,798,588.266	24.28829916	19.82634087	28.75025744
2029	2,412,302.837	1,901,190.098	2,923,415.577	24.42428731	19.24790148	29.60067314
2030	2,460,691.249	1,864,087.463	3,057,295.035	24.56494378	18.60750393	30.52238364
2031	2,509,281.206	1,818,613.66	3,199,948.752	24.70728852	17.90493525	31.50964179
2032	2,558,349.904	1,764,835.545	3,351,864.262	24.85389419	17.14296915	32.56481923
2033	2,608,312.699	1,702,902.231	3,513,723.168	25.009207	16.32545597	33.69295802
2034	2,659,348.654	1,632,622.122	3,686,075.186	25.1738668	15.45182326	34.89591034
Incidence						
2035	2,711,236.709	1,553,407.656	3,869,065.761	25.34535462	14.51822878	36.17248045
2036	2,763,595.355	1,464,488.147	4,062,702.564	25.51984692	13.51932259	37.52037125
2037	2,816,711.357	1,365,397.526	4,268,025.188	25.69988671	12.45279369	38.94697973
2038	2,871,291.431	1,255,824.717	4,486,758.146	25.89172212	11.31791693	40.46552731
2039	2,927,677.697	1,135,043.031	4,720,312.363	26.09674793	10.10966755	42.08382831
2040	2,985,513.084	1,001,855.51	4,969,170.659	26.31233501	8.819931235	43.80473878
2041	3,044,220.573	855,028.6903	5,233,561.846	26.53436455	7.441423473	45.62959832
2042	3,104,003.734	693,474.925	5,515,126.782	26.7654602	5.967876218	47.57193676
2043	3,165,534.757	536,213.4027	5,816,461.786	27.0138203	4.565001822	49.65624271
2044	3,229,353.833	427,936.1297	6,140,327.542	27.28166259	3.601524605	51.89954873
2045	3,295,386.091	310,305.2233	6,488,732.469	27.56641131	2.580469667	54.31248497
Deaths						
2021	44,760.95774	42,192.45689	47,329.45859	0.46841696	0.462508854	0.474325067
2022	46,231.76868	43,316.85013	49,146.68724	0.466710565	0.45512003	0.4783011
2023	47,261.29812	44,050.40707	50,472.18917	0.465517933	0.44812822	0.482907646
2024	48,368.13863	44,700.29935	52,035.97792	0.464383477	0.439950131	0.488816822
2025	49,517.6834	45,226.46188	53,808.90492	0.463299052	0.430858188	0.495739915
2026	50,680.17589	45,603.12499	55,757.22679	0.462245523	0.420973195	0.503517852
2027	51,832.3467	45,815.48178	57,849.21162	0.461214927	0.410373808	0.512056046
2028	53,022.82318	45,917.38724	60,128.25913	0.460255691	0.399191563	0.521319818
2029	54,285.5355	45,942.22705	62,628.84395	0.459375912	0.38748936	0.531262464
2030	55,591.38452	45,863.85274	65,318.91631	0.45856876	0.375294899	0.541842621
2031	56,915.29895	45,658.40761	68,172.19028	0.457816856	0.362612554	0.553021159
2032	58,235.24763	45,305.62398	71,164.87128	0.457106983	0.349447945	0.564766021
2033	59,588.58749	44,835.6369	74,341.53808	0.45645676	0.335850517	0.577063004
2034	61,004.9501	44,266.38872	77,743.51147	0.455852211	0.321834035	0.589870387
2035	62,461.74505	43,573.29782	81,350.19228	0.455278947	0.307402913	0.603154981
2036	63,939.01558	42,733.75213	85,144.27902	0.454724303	0.292555847	0.61689276
2037	65,420.11976	41,728.8714	89,111.36812	0.454183252	0.277296625	0.631069878
2038	66,946.21776	40,579.97457	93,312.46096	0.453666978	0.261655363	0.645678593
2039	68,544.52548	39,293.13768	97,795.91328	0.453161307	0.245640187	0.660682427
2040	70,188.11048	37,839.5029	102,536.904	0.452659323	0.229257878	0.676060767
2041	71,853.54593	36,193.2851	107,514.4643	0.452162534	0.212514297	0.69181077
2042	73,520.27473	34,332.00859	112,709.6707	0.451671684	0.195414109	0.707929258
2043	75,224.81505	32,264.13199	118,187.8901	0.451183383	0.177972919	0.724393848
2044	76,997.53699	29,986.83198	124,012.5604	0.450677414	0.160196359	0.741158469
2045	78,818.09525	27,472.47353	130,170.7119	0.450147981	0.142092907	0.758203055

ASR = age-standardized rate.

## 4. Discussion

This study provides the latest data on global, regional, and national levels regarding the incidence, prevalence, deaths, and DALY of TC from 1990 to 2021, and further presents a comprehensive evaluation based on trend, decomposition, inequality, and predictive analyses. Despite significant variations in the incidence, prevalence, deaths, and DALY of TC across countries, the global burden of TC has generally increased from 1990 to 2021, with the fastest growth occurring in the first decade of the 21st century. Decomposition analysis identified population growth as the primary factor driving changes in the burden of TC, with aging being the secondary factor. The cross-national inequality analysis indicated that high SDI countries bear a disproportionate burden of TC, and the inequalities associated with SDI have intensified over time. Notably, our predictive analysis suggests that from 2020 to 2045, both the number of cases and ASR for incidence and prevalence of TC are expected to rise annually, while although the ASR of deaths is projected to decline, the number of deaths is anticipated to continue increasing. This indicates that significant challenges in the control and management of TC will persist in the coming decades.

In 2021, TC accounted for 1,987,148 prevalent cases, 249,538 new cases, 44,799 deaths, and 1,246,485 DALY globally. The highest number of incident and prevalent cases was reported in the East Asia region, while the highest number of deaths and DALY was observed in the South Asia region. High-income North America exhibited the highest ASR for incidence and prevalence, the Andean Latin America region had the highest ASR of deaths, and Eastern Sub-Saharan Africa showed the highest ASR for DALY. It is noteworthy that we should not only focus on regions currently bearing the heaviest burden but also on those experiencing the most significant growth over the past few decades. Therefore, the Andean Latin America region, which experienced the largest increases in ASR for incidence and prevalence from 1990 to 2021, as well as South Asia, which had the highest increases in mortality and DALY, warrant special attention. This indicates that these regions should invest more in TC prevention to address emerging trends. At the national level, the differences in the burden of TC and their temporal trends among countries suggest that flexible health policies should be formulated based on the specific circumstances of different countries and regions. In 2021, the top 3 countries with the highest ASR for incidence and prevalence of TC in Western Europe were France, Italy, and Iceland, while Japan had the highest ASR of deaths. China had the highest numbers of incident, prevalent, and deaths cases. Notably, even with high levels of clinical and public health expenditure, developed countries such as France, Italy, and Iceland have been unable to correct the high incidence and prevalence of TC. Countries in Western Europe experienced a significant increase in the elderly population during the latter half of the 20th century, leading to long-term population aging, which may be a contributing factor to the highest ASR for TC in this region to date.^[24]^ Fortunately, developed countries in Western Europe benefit from advanced medical technologies and ample research funding, which provide a greater impetus for conducting more fundamental research and implementing clinical practices for the control of TC. This offers valuable experience for countries worldwide, particularly those in less favorable conditions. In terms of incidence, prevalence, and deaths, China ranks first in the number of TC cases, primarily due to its large population. The vast number of cases, unfavorable temporal trends over the past few decades, and uneven distribution of medical resources may pose significant challenges to the management of TC in populous countries.

Similar to previous studies,^[7,8,25]^ our research observed an overall increasing trend in the number and ASR of TC incidence and prevalence from 1990 to 2021, indicating that the burden of TC has increased over time on a global scale. On one hand, this increasing trend may be attributed to heightened health awareness and advancements in diagnostic techniques, leading to more thyroid malignancies being detected and treated at earlier stages.^[26]^ However, it is important to note that TC not only imposes direct economic burdens due to medication and surgical treatments but also generates indirect financial burdens through lost workdays and disability leave. Thus, TC is recognized as a cancer type that poses a heavy financial burden on patients.^[27]^ Simultaneously, as the young age of disease,^[3]^ the burden of overtreatment^[28]^ and the associated psychological stress^[29]^ suggest the necessity of developing preventive strategies, improving management, and implementing evidence-based treatments for TC.

When the overall trend was divided into multiple subsegments, we observed that prior to 2009, there was a significant increase in the ASRs of TC incidence and prevalence. This may be attributed to increased awareness of the disease and advancements in medical care, which have led to a substantial rise in the detection rates of TC. By the end of the study period (2009–2021), the ASR of incidence and prevalence of TC continued to rise, but the growth rate significantly slowed compared to the period before 2009. We also noted that during this phase, the trends in deaths and DALY were relatively stable and exhibited an overall declining trend. This indicates that the development of effective prevention, management, and treatment strategies has had a significant impact on suppressing the incidence of TC.^[30]^ Considering gender as a reference, from 1990 to 2021, the incidence, prevalence, deaths, and DALY for TC were all higher in females than in males. In 2021, the burden of prevalence for female TC was more than twice that of males (31.33 vs 14.79). This difference is primarily determined by levels of estrogen, differences in dietary patterns, and variations in the expression of hormone receptors.^[31,32]^ However, our results also found that the EAPC and average annual percentage change for incidence, prevalence, deaths, and DALY were higher in males compared to females, indicating that the burden of TC is increasing more rapidly in males. This emphasizes the need for further attention to the early detection and management of TC in males and highlights the importance of improving healthcare practices and management strategies to alleviate the burden on healthcare systems.

Age, period, and birth cohort effects represent 3 dimensions through which individuals and the societies in which they live change over time.^[13]^ Therefore, exploring the temporal trends of TC, particularly in relation to age, period, and birth cohort effects, can enhance our understanding of the epidemiology of the disease. We observed that the RR of incidence and prevalence exhibited a trend of initially increasing and then decreasing with advancing age, peaking in the 55 to 59 age group, followed by a gradual decline. Thus, individuals in the 55 to 59 age group should be targeted with more specific prevention, management, and treatment measures. However, it is noteworthy that the RR of incidence for individuals over the age of 35 is >1, and the RR of prevalence for those over 30 is also >1. Therefore, from the age of 30 to 35, there may be a need to strengthen health education programs related to TC. Additionally, the RR of mortality shows a trend of increase with advancing age, indicating that older individuals face a higher RR of death. This may be related to an increase in other risk factors associated with aging, suggesting that these risk factors may require further exploration. Regarding period effects, we detected that the RRs of incidence, prevalence, and deaths increase with the progression of time, which may be closely associated with advancements in medical technology and the annual increase in TC detection rates. It is important to note that the estimates from the GBD 2021 are derived from the latest available epidemiological data. Therefore, improving health registration systems over time may also contribute to the increased burden of TC. As for birth cohort effects, we observed that the RRs of incidence, prevalence, and deaths generally exhibit a decreasing trend along the birth cohort, indicating that individuals born earlier show a higher risk of TC compared to those born later. On one hand, earlier-born individuals may have experienced more risk factors throughout their lives compared to those born later. On the other hand, individuals born later appear to benefit from better healthcare services, improved lifestyles, and enhanced medical care compared to those born in earlier cohorts.

Our decomposition analysis indicates that the increase in global DALY due to TC over the past 30 years is primarily attributed to population growth and aging. The United Nations’ “World Population Prospects 2024” report projects that the global population will reach nearly 8.2 billion by mid-2024, with an expected increase of 2 billion over the next 60 years, peaking at approximately 10.3 billion in the mid-2080s.^[33]^ It also highlights that the pace of population aging is accelerating, with the population aged 65 and older expected to exceed that of individuals under 18 by the late 2070s.^[33]^ Furthermore, data from the World Health Organization indicates that the proportion of the population aged 60 and over is projected to increase from 12% in 2015 to 22% by 2050.^[34]^ This analysis suggests that countries around the world should enhance their healthcare systems to adequately prepare for and adapt to this rapid population growth trend, particularly focusing on aging populations and the allocation of healthcare resources. Notably, the demographic changes associated with both population growth and aging may be significant factors contributing to the anticipated increase in TC DALY up to 2045.

Quantifying the health inequalities in the burden of TC across different SDI gradient regions can clarify the sources of this burden and facilitate the development of tailored strategies to address the specific needs of different SDI areas. It is generally believed that individuals in high SDI countries have greater access to healthcare systems, which perform better, potentially leading to lower disease burdens. However, a disproportionate burden of TC incidence and prevalence has been observed in countries with high levels of socio-demographic development. A study based on the GBD 2019 data predicts a significant positive correlation between SDI and the ASR of TC incidence from 1990 to 2030.^[8]^ Another study indicated a positive correlation between the ASR of TC incidence and the universal health coverage index, as well as the density of healthcare workers.^[35]^ The imbalance in the burden of TC in high SDI countries can primarily be attributed to 2 factors. First, high SDI countries often have a higher proportion of obese populations, which is associated with an increased risk of TC,^[36]^ thereby promoting the incidence of TC. On the other hand, diagnostic biases arising from differences in access to healthcare opportunities across regions with varying SDI levels contribute to this trend; high SDI areas have higher detection rates, leading to a significant increase in incidence and prevalence. Cross-country inequality analysis also shows that the incidence and prevalence of TC displayed an increasing positive slope index and a decreasing concentration index from 1990 to 2021, indicating an imbalanced distribution of the burden among countries with different SDI levels. This reflects an increasingly apparent wealth gap while showing some improvement in concentration levels. Additionally, the SDI index is thought to have a negative correlation with the ASR of TC mortality.^[8]^ In lower SDI regions, the lack of medical resources exacerbates the challenges in addressing mortality cases arising from the TC epidemic. To narrow these gaps, targeted policies should be implemented to allocate medical resources effectively. On one hand, high SDI countries should focus on developing more evidence-based early diagnostic standards while reducing adverse factors that promote the occurrence and progression of TC and promptly devising treatment management strategies to address the growing elderly population. On the other hand, lower SDI countries face significant challenges due to inadequate medical resources and population growth. The World Health Organization and the United Nations should advocate for global action to provide more medical assistance and guidance to these countries.

Based on the development trends of TC burden over the past 32 years, we also predicted the trends of TC burden for the next 25 years. Using a generalized linear model, it is anticipated that by 2045, the ASR of incidence and prevalence of TC in China and globally will continue to rise, while the ASR of deaths is expected to decline. This provides a direction for future prevention and treatment of TC. Notably, the absolute numbers for incidence, prevalence, and deaths will all increase, indicating that the control and management of TC face a heavy disease burden and new challenges. We analyzed the significant factors contributing to the increasing burden of TC. First, this predictive indicator is closely related to the substantial growth of the world’s population and the severity of population aging, with changes in lifestyle behaviors being previously mentioned. Population growth and aging are important reasons for the significant increase in the number of DALY due to TC and inevitably exacerbate the global disease burden of TC up to 2045. It is noteworthy that there remains controversy regarding the issue of overtreatment in TC,^[37,38]^ which is considered a significant factor contributing to the continued rise in incidence and prevalence. A study based on the GBD 2019 database indicated that the phenomenon of overtreatment is more pronounced in middle- and high-income countries, leading to a contrasting pattern between the incidence and deaths rates of TC.^[35]^ In the future, standardized diagnostic and treatment protocols are expected to help reduce the disease burden associated with this factor. Furthermore, we should focus on reducing the incidence and prevalence of TC through early detection, diagnosis, and treatment. Research has shown that other risk factors, such as obesity and a history of radiation exposure, may be associated with the incidence and mortality of TC.^[39,40]^ Therefore, lifestyle changes and the avoidance of risk factors, such as weight management and reducing occupational exposure, can effectively mitigate the burden of TC.

Our study utilized the GBD database to conduct a comprehensive analysis of TC. However, it does have certain limitations. First, our predictions are based on the GBD, which means that the quality of the underlying raw data from individual registries greatly influences the accuracy of the estimates within the database. Although GBD data is extensive, differences in diagnostic standards, data collection methods, and healthcare systems across various regions of the world may introduce information bias, leading to concerns regarding the accuracy and completeness of the GBD data.^[6,41]^ Second, while our analysis of the burden of TC across different SDI regions provides some guidance for clinical practice, it may not fully reflect the complex socio-cultural and economic factors unique to these regions. Therefore, it should only serve as a macro-level directional basis for healthcare policy, and we should also develop tailored public health strategies for specific areas. Additionally, the data used in the study are aggregate rather than individual-level data, and the lack of detailed dynamic variables means that it can only reflect the burden factors of TC in specific regions. Furthermore, since TC is one of the smallest subsets of diseases within the GBD database, we were unable to reveal the epidemiological characteristics of TC across different stages and histological types. However, it is anticipated that as the GBD continues to develop and mature, its estimation techniques will become increasingly accurate and reliable. These statistical estimates provide a more comprehensive and continuous view of disease epidemiology than relying solely on isolated studies. In the future, they will have greater potential to guide clinical practice and offer insights for diagnostic and treatment directions.

Our study emphasizes the necessity of tailored strategies for the prevention, early detection, and treatment of TC, particularly in high-risk regions and populations. While providing a comprehensive global overview of TC, this research also highlights the need for detailed studies on the unique challenges posed by TC in different historical contexts. This work offers valuable insights and identifies areas for future research and interventions. We stress the importance of personalizing attention to the development trends of TC across different regions to formulate appropriate policies, as well as the need for proactive screening in high-risk age groups and populations with risk factors. Future research should integrate environmental, genetic, and socioeconomic factors to enable a more comprehensive analysis of the disease burden of TC across different regions and countries.

## 5. Conclusions

In recent years, the rising incidence of TC has posed a significant burden on global healthcare systems, and this situation is expected to worsen over the next 25 years. Blindly advocating for early screening may not effectively address this issue. As a major public health concern, there are substantial differences in the incidence, prevalence, deaths, and DALY of TC across countries. However, the global burden of TC has shown an overall increasing trend from 1990 to 2021, with the fastest growth occurring in the first decade of the 21st century, particularly concentrated among women and in the East Asia region. The changes in the burden of TC are primarily driven by population growth and aging, which may further lead to a sharp increase in the number of TC cases in the coming decades. Countries with high SDI levels bear a disproportionate burden of TC, and the cross-national inequalities in incidence and prevalence related to the SDI index have intensified over time. These findings collectively underscore the significant challenges faced in the control and management of TC, including the increasing number of cases and the inequalities in global distribution, which could inform better public health policies and allocation of healthcare resources. Global health policymakers should further consider targeted interventions to address the distinct healthcare needs of each country, aiming to create a personalized healthcare system on a global scale.

## Acknowledgments

We hereby confirm that neither the manuscript nor any part of it has been published or is being considered for publication elsewhere. We acknowledge that all authors sufficiently participated in the study and take responsibility for its content.

## Author contributions

**Conceptualization:** Mingxin Shen, Yutong Liu, Liang Shao.

**Data curation:** Mingxin Shen, Yutong Liu.

**Formal analysis:** Fan Yang.

**Funding acquisition:** Wei Sun, Hao Zhang.

**Investigation:** Mingxin Shen, Yutong Liu, Liang Shao, Fan Yang.

**Methodology:** Mingxin Shen, Liang Shao, Fan Yang, Wei Sun, Hao Zhang.

**Resources:** Mingxin Shen, Fan Yang.

**Software:** Mingxin Shen.

**Supervision:** Wei Sun, Hao Zhang.

**Validation:** Hao Zhang.

**Writing – original draft:** Mingxin Shen, Yutong Liu.

**Writing – review & editing:** Mingxin Shen, Wei Sun, Hao Zhang.









## References

[1] Sung H Ferlay J Siegel RL. Global cancer statistics 2020: GLOBOCAN estimates of incidence and mortality worldwide for 36 cancers in 185 countries. CA Cancer J Clin. 2021;71:209–49. 33538338 10.3322/caac.21660

[2] Pstrąg N Ziemnicka K Bluyssen H Wesoły J. Thyroid cancers of follicular origin in a genomic light: in-depth overview of common and unique molecular marker candidates. Mol Cancer. 2018;17:116. 30089490 10.1186/s12943-018-0866-1 PMC6081953

[3] Bao W Zi H Yuan Q Li L Deng T. Global burden of thyroid cancer and its attributable risk factors in 204 countries and territories from 1990 to 2019. Thorac Cancer. 2021;12:2494–503. 34355519 10.1111/1759-7714.14099 PMC8447914

[4] Pizzato M Li M Vignat J. The epidemiological landscape of thyroid cancer worldwide: GLOBOCAN estimates for incidence and mortality rates in 2020. Lancet Diabetes Endocrinol. 2022;10:264–72. 35271818 10.1016/S2213-8587(22)00035-3

[5] Chen DW Lang BHH McLeod DSA Newbold K Haymart MR. Thyroid cancer. Lancet. 2023;401:1531–44. 37023783 10.1016/S0140-6736(23)00020-X

[6] Murray CJL. The global burden of disease study at 30 years. Nat Med. 2022;28:2019–26. 36216939 10.1038/s41591-022-01990-1

[7] Hu S Wu X Jiang H. Trends and projections of the global burden of thyroid cancer from 1990 to 2030. J Glob Health. 2024;14:04084. 38751316 10.7189/jogh.14.04084 PMC11109522

[8] Zhao Q Chen M Fu L Yang Y Zhan Y. Assessing and projecting the global burden of thyroid cancer, 1990–2030: analysis of the global burden of disease study. J Glob Health. 2024;14:04090. 38577809 10.7189/jogh.14.04090 PMC10995745

[9] Institute of Health Metrics and Evaluation. Global Burden of Disease Study 2019 (GBD 2019) data resources. Available from: https://ghdx.healthdata.org/gbd-2021 Accessed August 28, 2025.

[10] Dai H Alsalhe TA Chalghaf N Riccò M Bragazzi NL Wu J. The global burden of disease attributable to high body mass index in 195 countries and territories, 1990–2017: an analysis of the global burden of disease study. PLoS Med. 2020;17:e1003198. 32722671 10.1371/journal.pmed.1003198 PMC7386577

[11] Liu Z Jiang Y Yuan H. The trends in incidence of primary liver cancer caused by specific etiologies: results from the global burden of disease study 2016 and implications for liver cancer prevention. J Hepatol. 2019;70:674–83. 30543829 10.1016/j.jhep.2018.12.001

[12] Partitioning linear trends in age ad source cancer causes control so 2000 Jan 11 1 31 5.Pdf.Pdf. 10.1023/a:1008953201688 10680727

[13] Bell A. Age period cohort analysis: a review of what we should and shouldn’t do. Ann Hum Biol. 2020;47:208–17. 32429768 10.1080/03014460.2019.1707872

[14] Dal Maso L Lise M Zambon P. Incidence of thyroid cancer in Italy, 1991–2005: time trends and age-period-cohort effects. Ann Oncol. 2011;22:957–63. 20952599 10.1093/annonc/mdq467

[15] Dong Z Wang Q-Q Yu S-C. Age-period-cohort analysis of pulmonary tuberculosis reported incidence, China, 2006–2020. Infect Dis Poverty. 2022;11:85. 35902982 10.1186/s40249-022-01009-4 PMC9331155

[16] Lv H Wang L Zhang X. Further analysis of tuberculosis in eight high-burden countries based on the global burden of disease study 2021 data. Infect Dis Poverty. 2024;13:70. 39343935 10.1186/s40249-024-01247-8 PMC11440896

[17] Gupta PD. Standardization and decomposition of rates from cross-classified data. Genus. 1994;50:171–96. 12319256

[18] Fitzmaurice C; Global Burden of Disease Cancer Collaboration. Global, regional, and national cancer incidence, mortality, years of life lost, years lived with disability, and disability-adjusted life-years for 29 cancer groups, 2006 to 2016: a systematic analysis for the global burden of disease study. JCO. 2018;36 (15_suppl):1568–1568. 10.1001/jamaoncol.2018.2706 PMC6248091 29860482

[19] Moreno-Betancur M Latouche A Menvielle G Kunst AE Rey G. Relative index of inequality and slope index of inequality: a structured regression framework for estimation. Epidemiology. 2015;26:518–27. 26000548 10.1097/EDE.0000000000000311

[20] Koolman X Van Doorslaer E. On the interpretation of a concentration index of inequality. Health Econ. 2004;13:649–56. 15259044 10.1002/hec.884

[21] Mújica J Moreno CM. [From words to action: measuring health inequalities to “leave no one behind” Da retórica à ação: mensurar as desigualdades em saúde para não deixar ninguém atrás]. Rev Panam Salud Publica. 2019;43:e12–8. 31093236 10.26633/RPSP.2019.12 PMC6393735

[22] Jürgens V Ess S Cerny T Vounatsou P. A Bayesian generalized age-period-cohort power model for cancer projections. Stat Med. 2014;33:4627–36. 24996118 10.1002/sim.6248

[23] Hu W Fang L Zhang H Ni R Pan G. Global disease burden of COPD from 1990 to 2019 and prediction of future disease burden trend in China. Public Health. 2022;208:89–97. 35728417 10.1016/j.puhe.2022.04.015

[24] Bos D Von Weizsacker RK. Economic consequences of an aging population. Europ Econ Rev. 1989;33:345–54. 10.1016/0014-2921(89)90112-8 12316297

[25] Deng Y Li H Wang M. Global burden of thyroid cancer from 1990 to 2017. JAMA Netw Open. 2020;3:e208759. 32589231 10.1001/jamanetworkopen.2020.8759 PMC7320301

[26] Kitahara CM. The changing incidence of thyroid cancer. 10.1038/nrendo.2016.110 PMC10311569 27418023

[27] Uppal N Cunningham (Nee Lubitz) C James B. The cost and financial burden of thyroid cancer on patients in the US. JAMA Otolaryngol Head Neck Surg. 2022;148:568. 35511135 10.1001/jamaoto.2022.0660

[28] Kitahara CM. The growing global burden of thyroid cancer overdiagnosis. Lancet Diabetes Endocrinol. 2024;12:780–2. 39389068 10.1016/S2213-8587(24)00269-9 PMC13006842

[29] Drabe N Steinert H Moergeli H Weidt S Strobel K Jenewein J. Perception of treatment burden, psychological distress, and fatigue in thyroid cancer patients and their partners - effects of gender, role, and time since diagnosis. Psychooncology. 2016;25:203–9. 26179844 10.1002/pon.3903

[30] Kobaly K Kim CS Mandel SJ. Contemporary management of thyroid nodules. Annu Rev Med. 2022;73:517–28. 34416120 10.1146/annurev-med-042220-015032

[31] Kirsch-Volders M Bonassi S Herceg Z Hirvonen A Moller L Phillips DH. Gender-related differences in response to mutagens and carcinogens. Mutagenesis. 2010;25:213–21. 20194421 10.1093/mutage/geq008

[32] Rahbari R Zhang L Kebebew E. Thyroid cancer gender disparity. Future Oncol. 2010;6:1771–9. 21142662 10.2217/fon.10.127 PMC3077966

[33] World Population Prospects 2024. Available from: https://population.un.org/wpp Accessed August 15, 2025.

[34] World Health Organization. Ageing and health. Available from: https://www.who.int/news-room/fact-sheets/detail/ageing-and-health. Accessed August 11, 2025.

[35] Wang C Wu Z Lei L. Geographic disparities in trends of thyroid cancer incidence and mortality from 1990 to 2019 and a projection to 2030 across income-classified countries and territories. J Glob Health. 2023;13:04108. 37766638 10.7189/jogh.13.04108 PMC10540248

[36] Burrage LC McLeod DSA Jordan SJ. Obesity and thyroid cancer risk. Curr Opin Endocrinol Diabetes Obes. 2023;30:244–51. 37410455 10.1097/MED.0000000000000825

[37] Georgiades C. Slow growth, excellent prognosis: the treatment and overtreatment of papillary thyroid cancer. Radiology. 2024;311:e240207. 38563672 10.1148/radiol.240207

[38] Luster M Weber T Verburg FA. Differentiated thyroid cancer-personalized therapies to prevent overtreatment. Nat Rev Endocrinol. 2014;10:563–74. 24981455 10.1038/nrendo.2014.100

[39] Hidayat K Du X Shi B‐M. Body fatness at a young age and risks of eight types of cancer: systematic review and meta‐analysis of observational studies. Obes Rev. 2018;19:1385–94. 30047231 10.1111/obr.12705

[40] Greenspan FS. Radiation exposure and thyroid cancer. JAMA. 1977;237:2089–91. 576889

[41] GBD 2019 Diseases and Injuries Collaborators. Global burden of 369 diseases and injuries in 204 countries and territories, 1990–2019: a systematic analysis for the Global Burden of Disease Study 2019. Lancet. 2020;396:1204–22. 33069326 10.1016/S0140-6736(20)30925-9 PMC7567026

