# Supplementary material for: Trends and cross-country inequalities in the global burden of thyroid cancer, 1990–2021: A population-based study
Source: Medicine (Baltimore). 2026 Aug 14;105(33):e50237. doi: 10.1097/MD.0000000000050237 (PMC13480896; doi:10.1097/MD.0000000000050237)
Supplement: Supplementary file 1 [file medi-105-e50237-s001.docx]

| Location  Table S1. The case number and ASR of incidence of TC in 2021 by countries/territories. | Number | Number_upper | Number_lower | Rate | Rate_upper | Rate_lower |
| --- | --- | --- | --- | --- | --- | --- |
| China | 48104.556 | 60068.113 | 38694.775 | 3.381 | 4.222 | 2.720 |
| India | 28334.100 | 34107.112 | 22851.162 | 2.003 | 2.411 | 1.615 |
| United States of America | 26396.293 | 27472.174 | 25051.513 | 7.935 | 8.259 | 7.531 |
| Japan | 10066.624 | 10909.737 | 9098.574 | 7.883 | 8.543 | 7.125 |
| Indonesia | 7343.259 | 9783.027 | 4749.232 | 2.633 | 3.508 | 1.703 |
| Viet Nam | 6995.605 | 9263.305 | 4791.160 | 6.977 | 9.239 | 4.778 |
| Russian Federation | 6782.051 | 7461.252 | 6175.336 | 4.682 | 5.151 | 4.263 |
| France | 6261.302 | 7462.670 | 5174.955 | 9.431 | 11.241 | 7.795 |
| Pakistan | 5476.036 | 7662.239 | 3849.345 | 2.325 | 3.253 | 1.634 |
| Italy | 5311.707 | 5869.804 | 4772.367 | 8.881 | 9.814 | 7.979 |
| Germany | 4968.563 | 5935.670 | 4104.070 | 5.820 | 6.953 | 4.807 |
| Brazil | 4323.925 | 4580.299 | 4024.592 | 1.962 | 2.079 | 1.826 |
| Turkey | 4240.036 | 5745.738 | 3188.872 | 5.071 | 6.872 | 3.814 |
| Philippines | 4113.473 | 5119.597 | 3321.362 | 3.632 | 4.521 | 2.933 |
| Mexico | 3987.785 | 4535.972 | 3477.770 | 3.085 | 3.509 | 2.690 |
| Republic of Korea | 3922.884 | 5824.022 | 2980.137 | 7.607 | 11.293 | 5.779 |
| Thailand | 3800.984 | 5756.886 | 2727.412 | 5.700 | 8.633 | 4.090 |
| Iran (Islamic Republic of) | 3290.984 | 4115.729 | 1397.750 | 3.856 | 4.822 | 1.638 |
| Bangladesh | 2983.088 | 5392.640 | 1874.984 | 1.812 | 3.275 | 1.139 |
| Saudi Arabia | 2942.624 | 3917.141 | 2097.695 | 7.805 | 10.389 | 5.564 |
| Ethiopia | 2701.319 | 4539.811 | 1775.101 | 2.480 | 4.167 | 1.629 |
| United Kingdom | 2592.659 | 2695.101 | 2464.106 | 3.821 | 3.972 | 3.632 |
| Colombia | 2086.958 | 2599.239 | 1686.513 | 4.254 | 5.298 | 3.438 |
| Egypt | 2055.012 | 2710.107 | 1530.140 | 1.946 | 2.566 | 1.449 |
| Taiwan (Province of China) | 2004.243 | 2263.706 | 1726.522 | 8.479 | 9.577 | 7.304 |
| Spain | 1938.553 | 2301.631 | 1619.580 | 4.256 | 5.053 | 3.556 |
| Canada | 1891.052 | 2289.056 | 1549.371 | 5.047 | 6.109 | 4.135 |
| Ukraine | 1791.651 | 2539.723 | 1234.668 | 4.159 | 5.896 | 2.866 |
| Australia | 1725.353 | 2106.433 | 1383.803 | 6.690 | 8.167 | 5.365 |
| Poland | 1631.654 | 1829.685 | 1450.122 | 4.267 | 4.785 | 3.792 |
| Algeria | 1596.869 | 2117.461 | 1105.436 | 3.613 | 4.791 | 2.501 |
| Iraq | 1544.800 | 2119.418 | 1111.375 | 3.747 | 5.141 | 2.696 |
| Myanmar | 1399.608 | 1860.415 | 941.110 | 2.481 | 3.297 | 1.668 |
| Peru | 1329.623 | 1839.848 | 928.407 | 3.666 | 5.073 | 2.560 |
| Malaysia | 1279.636 | 1547.969 | 1061.393 | 4.022 | 4.866 | 3.336 |
| Argentina | 1170.397 | 1414.172 | 964.083 | 2.573 | 3.109 | 2.119 |
| Sri Lanka | 835.385 | 1203.327 | 507.593 | 3.751 | 5.403 | 2.279 |
| Morocco | 834.438 | 1204.054 | 564.375 | 2.245 | 3.239 | 1.518 |
| Uganda | 803.790 | 1127.566 | 535.832 | 1.856 | 2.603 | 1.237 |
| Democratic People's Republic of Korea | 776.381 | 1105.023 | 531.835 | 2.942 | 4.187 | 2.015 |
| United Republic of Tanzania | 749.410 | 1149.874 | 497.394 | 1.282 | 1.967 | 0.851 |
| Location  Table S1 (continued) | Number | Number_upper | Number_lower | Rate | Rate_upper | Rate_lower |
| Chile | 726.260 | 868.845 | 620.248 | 3.864 | 4.622 | 3.300 |
| Venezuela (Bolivarian Republic of) | 724.222 | 941.505 | 542.249 | 2.720 | 3.536 | 2.036 |
| South Africa | 716.457 | 879.966 | 614.688 | 1.260 | 1.548 | 1.081 |
| Ecuador | 714.080 | 900.369 | 549.697 | 3.953 | 4.984 | 3.043 |
| Austria | 689.851 | 825.611 | 569.612 | 7.680 | 9.192 | 6.341 |
| Romania | 674.657 | 871.320 | 525.136 | 3.562 | 4.601 | 2.773 |
| Portugal | 644.596 | 800.157 | 513.077 | 6.077 | 7.543 | 4.837 |
| Czechia | 639.513 | 828.071 | 486.650 | 6.015 | 7.788 | 4.577 |
| Sudan | 625.811 | 998.270 | 360.477 | 1.441 | 2.299 | 0.830 |
| Belgium | 590.203 | 716.553 | 479.275 | 5.146 | 6.248 | 4.179 |
| Kazakhstan | 589.504 | 705.342 | 479.791 | 3.110 | 3.721 | 2.531 |
| Netherlands | 586.467 | 688.346 | 489.996 | 3.408 | 4.000 | 2.847 |
| Cuba | 559.031 | 661.549 | 465.746 | 4.961 | 5.870 | 4.133 |
| Belarus | 545.794 | 698.207 | 426.794 | 5.853 | 7.488 | 4.577 |
| Cambodia | 544.325 | 771.828 | 340.635 | 3.194 | 4.528 | 1.999 |
| Nepal | 528.544 | 822.510 | 351.366 | 1.698 | 2.642 | 1.129 |
| Tunisia | 522.489 | 707.507 | 351.422 | 4.412 | 5.974 | 2.967 |
| Afghanistan | 509.316 | 855.137 | 227.270 | 1.631 | 2.739 | 0.728 |
| Greece | 451.659 | 508.713 | 399.320 | 4.439 | 5.000 | 3.925 |
| Jordan | 451.283 | 631.029 | 327.103 | 3.662 | 5.120 | 2.654 |
| Hungary | 448.564 | 535.337 | 365.069 | 4.674 | 5.578 | 3.804 |
| Libya | 446.066 | 629.651 | 306.946 | 6.492 | 9.164 | 4.468 |
| Syrian Arab Republic | 444.988 | 701.670 | 274.752 | 3.172 | 5.001 | 1.958 |
| Bolivia (Plurinational State of) | 380.341 | 542.307 | 250.820 | 3.224 | 4.597 | 2.126 |
| United Arab Emirates | 371.685 | 509.443 | 282.572 | 3.859 | 5.290 | 2.934 |
| Israel | 354.605 | 430.964 | 292.549 | 3.696 | 4.492 | 3.049 |
| Zambia | 352.561 | 720.778 | 168.464 | 1.807 | 3.693 | 0.863 |
| Yemen | 333.528 | 482.626 | 226.456 | 0.991 | 1.435 | 0.673 |
| Sweden | 328.881 | 396.604 | 269.343 | 3.170 | 3.823 | 2.596 |
| Mozambique | 328.764 | 607.857 | 197.936 | 1.058 | 1.956 | 0.637 |
| Zimbabwe | 327.734 | 456.700 | 205.924 | 2.101 | 2.928 | 1.320 |
| Serbia | 308.312 | 424.535 | 219.661 | 3.457 | 4.760 | 2.463 |
| Democratic Republic of the Congo | 301.626 | 498.965 | 182.393 | 0.335 | 0.554 | 0.203 |
| Madagascar | 296.459 | 410.908 | 198.598 | 1.038 | 1.439 | 0.695 |
| Switzerland | 277.539 | 328.100 | 231.177 | 3.110 | 3.677 | 2.591 |
| Kuwait | 274.732 | 326.863 | 219.367 | 5.908 | 7.029 | 4.717 |
| Singapore | 271.198 | 334.576 | 225.265 | 4.735 | 5.842 | 3.933 |
| Finland | 270.318 | 329.098 | 221.303 | 4.883 | 5.945 | 3.998 |
| Guatemala | 264.690 | 311.166 | 223.224 | 1.679 | 1.973 | 1.416 |
| Lebanon | 260.944 | 348.006 | 197.989 | 4.710 | 6.281 | 3.574 |
| Location  Table S1 (continued) | Number | Number_upper | Number_lower | Rate | Rate_upper | Rate_lower |
| Kenya | 253.406 | 397.377 | 179.453 | 0.506 | 0.794 | 0.358 |
| Bulgaria | 250.866 | 317.728 | 195.369 | 3.696 | 4.681 | 2.879 |
| Croatia | 248.618 | 302.269 | 201.707 | 5.907 | 7.182 | 4.793 |
| Uzbekistan | 245.105 | 333.810 | 179.422 | 0.716 | 0.975 | 0.524 |
| Slovakia | 234.621 | 318.050 | 179.109 | 4.321 | 5.858 | 3.299 |
| Malawi | 230.548 | 382.024 | 140.929 | 1.185 | 1.964 | 0.725 |
| Norway | 229.014 | 255.172 | 200.776 | 4.227 | 4.710 | 3.706 |
| New Zealand | 223.728 | 262.770 | 185.029 | 4.328 | 5.083 | 3.579 |
| Georgia | 207.656 | 257.832 | 168.570 | 5.757 | 7.148 | 4.673 |
| El Salvador | 200.657 | 252.944 | 155.710 | 3.111 | 3.921 | 2.414 |
| Costa Rica | 188.991 | 220.393 | 159.094 | 3.981 | 4.642 | 3.351 |
| Ireland | 187.996 | 226.310 | 156.578 | 3.805 | 4.580 | 3.169 |
| Rwanda | 186.727 | 290.099 | 125.363 | 1.407 | 2.186 | 0.945 |
| Dominican Republic | 172.902 | 231.084 | 128.416 | 1.570 | 2.098 | 1.166 |
| Paraguay | 167.130 | 218.573 | 123.050 | 2.331 | 3.049 | 1.716 |
| Lithuania | 166.425 | 204.926 | 131.865 | 6.100 | 7.511 | 4.833 |
| Coted'Ivoire | 163.079 | 238.637 | 109.874 | 0.585 | 0.857 | 0.394 |
| Mali | 160.597 | 244.046 | 106.704 | 0.666 | 1.012 | 0.443 |
| Lao People's Democratic Republic | 154.224 | 212.831 | 98.693 | 2.090 | 2.885 | 1.338 |
| Azerbaijan | 151.197 | 212.374 | 104.638 | 1.440 | 2.023 | 0.997 |
| Denmark | 150.373 | 180.411 | 126.993 | 2.570 | 3.083 | 2.170 |
| Uruguay | 150.127 | 183.132 | 124.735 | 4.408 | 5.377 | 3.663 |
| Kyrgyzstan | 147.962 | 199.827 | 105.312 | 2.156 | 2.912 | 1.534 |
| Armenia | 145.328 | 190.183 | 109.472 | 4.852 | 6.349 | 3.655 |
| Somalia | 144.729 | 213.583 | 90.774 | 0.670 | 0.989 | 0.420 |
| Palestine | 138.982 | 175.499 | 90.030 | 2.706 | 3.417 | 1.753 |
| Qatar | 134.701 | 185.757 | 94.934 | 4.525 | 6.240 | 3.189 |
| Puerto Rico | 133.714 | 163.477 | 107.483 | 4.059 | 4.962 | 3.263 |
| Haiti | 130.751 | 188.797 | 87.930 | 1.016 | 1.468 | 0.684 |
| Panama | 126.875 | 158.872 | 101.234 | 2.956 | 3.702 | 2.359 |
| Republic of Moldova | 126.361 | 150.906 | 106.418 | 3.516 | 4.199 | 2.961 |
| Latvia | 124.711 | 161.075 | 94.031 | 6.668 | 8.612 | 5.027 |
| Nigeria | 118.800 | 206.948 | 73.317 | 0.051 | 0.090 | 0.032 |
| Angola | 118.354 | 192.112 | 71.011 | 0.362 | 0.587 | 0.217 |
| Burundi | 115.264 | 184.899 | 74.184 | 0.872 | 1.399 | 0.561 |
| South Sudan | 105.572 | 165.500 | 68.668 | 1.091 | 1.711 | 0.710 |
| Bosnia and Herzegovina | 103.731 | 139.689 | 76.050 | 3.141 | 4.229 | 2.303 |
| Oman | 103.080 | 137.885 | 68.651 | 2.191 | 2.931 | 1.459 |
| Nicaragua | 102.318 | 129.740 | 76.325 | 1.534 | 1.945 | 1.144 |
| Turkmenistan | 86.351 | 116.196 | 65.615 | 1.674 | 2.253 | 1.272 |
| Slovenia | 84.261 | 113.030 | 61.108 | 4.071 | 5.461 | 2.953 |
| Eritrea | 81.289 | 130.564 | 52.494 | 1.232 | 1.979 | 0.796 |
| Location  Table S1 (continued) | Number | Number_upper | Number_lower | Rate | Rate_upper | Rate_lower |
| Bahrain | 80.267 | 108.722 | 59.206 | 5.248 | 7.108 | 3.871 |
| Jamaica | 80.240 | 108.112 | 58.532 | 2.866 | 3.861 | 2.090 |
| Estonia | 80.019 | 102.502 | 60.663 | 6.104 | 7.820 | 4.628 |
| Albania | 76.808 | 110.877 | 52.962 | 2.879 | 4.156 | 1.985 |
| Cameroon | 74.344 | 110.315 | 46.569 | 0.234 | 0.347 | 0.147 |
| Honduras | 70.141 | 96.237 | 48.574 | 0.694 | 0.952 | 0.480 |
| North Macedonia | 69.884 | 89.244 | 52.833 | 3.211 | 4.100 | 2.428 |
| Papua New Guinea | 66.684 | 118.591 | 35.552 | 0.637 | 1.134 | 0.340 |
| Mongolia | 56.744 | 77.912 | 42.453 | 1.701 | 2.335 | 1.272 |
| Cyprus | 52.311 | 72.524 | 39.195 | 3.853 | 5.341 | 2.887 |
| Guinea | 50.873 | 75.331 | 35.136 | 0.379 | 0.561 | 0.262 |
| Trinidad and Tobago | 44.789 | 58.521 | 33.642 | 3.215 | 4.201 | 2.415 |
| Burkina Faso | 38.700 | 54.250 | 23.381 | 0.170 | 0.238 | 0.103 |
| Congo | 36.225 | 57.507 | 21.932 | 0.672 | 1.067 | 0.407 |
| Montenegro | 33.734 | 42.671 | 26.307 | 5.458 | 6.904 | 4.256 |
| Fiji | 33.547 | 47.721 | 20.141 | 3.629 | 5.162 | 2.179 |
| Luxembourg | 32.609 | 37.815 | 28.443 | 5.061 | 5.870 | 4.415 |
| Senegal | 31.843 | 50.718 | 20.530 | 0.201 | 0.320 | 0.129 |
| Iceland | 30.581 | 37.549 | 25.230 | 8.728 | 10.717 | 7.201 |
| Mauritius | 28.279 | 31.356 | 24.806 | 2.223 | 2.465 | 1.950 |
| Niger | 25.640 | 38.758 | 15.935 | 0.102 | 0.155 | 0.064 |
| Ghana | 24.802 | 36.275 | 15.839 | 0.072 | 0.106 | 0.046 |
| Namibia | 24.392 | 35.582 | 15.915 | 1.003 | 1.464 | 0.655 |
| Malta | 22.695 | 27.999 | 18.361 | 5.132 | 6.332 | 4.152 |
| Chad | 21.941 | 30.537 | 14.712 | 0.124 | 0.172 | 0.083 |
| Benin | 20.486 | 31.842 | 12.834 | 0.152 | 0.236 | 0.095 |
| Botswana | 19.709 | 32.440 | 12.322 | 0.824 | 1.355 | 0.515 |
| Timor-Leste | 19.568 | 28.281 | 12.518 | 1.400 | 2.023 | 0.896 |
| Togo | 18.674 | 29.643 | 11.186 | 0.223 | 0.354 | 0.134 |
| Brunei Darussalam | 16.923 | 22.622 | 12.727 | 3.751 | 5.015 | 2.821 |
| Djibouti | 16.919 | 30.343 | 9.810 | 1.344 | 2.411 | 0.779 |
| Central African Republic | 16.789 | 25.936 | 10.870 | 0.306 | 0.473 | 0.198 |
| Lesotho | 16.436 | 25.477 | 10.992 | 0.877 | 1.359 | 0.586 |
| Sierra Leone | 15.651 | 23.538 | 9.270 | 0.177 | 0.265 | 0.105 |
| Gabon | 14.300 | 21.863 | 9.256 | 0.787 | 1.204 | 0.510 |
| Bhutan | 13.784 | 23.319 | 8.582 | 1.821 | 3.081 | 1.134 |
| Bahamas | 13.681 | 17.470 | 10.738 | 3.526 | 4.503 | 2.768 |
| Guyana | 12.850 | 17.216 | 9.557 | 1.680 | 2.251 | 1.250 |
| Liberia | 12.519 | 18.028 | 7.864 | 0.229 | 0.330 | 0.144 |
| Barbados | 12.089 | 15.393 | 9.532 | 4.043 | 5.148 | 3.188 |
| Eswatini | 12.054 | 19.005 | 7.093 | 1.043 | 1.645 | 0.614 |
| Comoros | 12.043 | 19.715 | 7.507 | 1.618 | 2.648 | 1.009 |
| Mauritania | 10.239 | 15.276 | 6.853 | 0.233 | 0.347 | 0.156 |
| Location  Table S1 (continued) | Number | Number_upper | Number_lower | Rate | Rate_upper | Rate_lower |
| Suriname | 9.658 | 12.294 | 7.070 | 1.667 | 2.122 | 1.220 |
| Equatorial Guinea | 8.448 | 14.117 | 4.464 | 0.559 | 0.933 | 0.295 |
| Gambia | 7.629 | 10.799 | 4.968 | 0.319 | 0.451 | 0.207 |
| Saint Lucia | 7.186 | 8.843 | 5.782 | 4.048 | 4.982 | 3.257 |
| Maldives | 6.173 | 8.385 | 4.607 | 1.194 | 1.621 | 0.891 |
| Solomon Islands | 5.698 | 8.742 | 3.456 | 0.834 | 1.279 | 0.506 |
| Samoa | 5.611 | 7.757 | 3.970 | 2.626 | 3.631 | 1.858 |
| Saint Vincent and the Grenadines | 5.315 | 6.185 | 4.555 | 4.659 | 5.422 | 3.993 |
| Belize | 5.168 | 5.914 | 4.480 | 1.204 | 1.378 | 1.044 |
| Bermuda | 4.708 | 5.940 | 3.775 | 7.409 | 9.348 | 5.942 |
| Guinea-Bissau | 4.438 | 6.230 | 3.031 | 0.215 | 0.302 | 0.147 |
| Andorra | 4.210 | 5.911 | 2.776 | 4.919 | 6.906 | 3.244 |
| Guam | 3.505 | 4.133 | 2.654 | 2.201 | 2.596 | 1.667 |
| Antigua and Barbuda | 3.414 | 3.731 | 3.122 | 3.819 | 4.174 | 3.492 |
| Grenada | 3.376 | 4.030 | 2.817 | 3.290 | 3.927 | 2.745 |
| Cabo Verde | 3.190 | 6.111 | 0.652 | 0.570 | 1.093 | 0.117 |
| Monaco | 2.690 | 3.504 | 1.973 | 7.104 | 9.253 | 5.212 |
| Vanuatu | 2.587 | 3.503 | 1.745 | 0.827 | 1.119 | 0.558 |
| San Marino | 2.246 | 3.287 | 1.321 | 6.862 | 10.038 | 4.033 |
| American Samoa | 1.830 | 2.376 | 1.268 | 3.677 | 4.776 | 2.548 |
| Micronesia (Federated States of) | 1.444 | 2.030 | 0.956 | 1.408 | 1.979 | 0.932 |
| Seychelles | 1.430 | 1.755 | 1.178 | 1.356 | 1.665 | 1.117 |
| Dominica | 1.340 | 1.706 | 1.041 | 1.998 | 2.543 | 1.552 |
| Greenland | 1.334 | 1.850 | 0.908 | 2.378 | 3.297 | 1.618 |
| United States Virgin Islands | 1.296 | 1.764 | 0.943 | 1.509 | 2.053 | 1.097 |
| Tonga | 1.291 | 1.868 | 0.799 | 1.215 | 1.757 | 0.752 |
| Saint Kitts and Nevis | 1.262 | 1.535 | 1.020 | 2.151 | 2.618 | 1.739 |
| Tajikistan | 1.066 | 1.557 | 0.722 | 0.010 | 0.015 | 0.007 |
| Northern Mariana Islands | 0.959 | 1.158 | 0.725 | 1.978 | 2.387 | 1.496 |
| Marshall Islands | 0.669 | 0.986 | 0.433 | 1.189 | 1.753 | 0.769 |
| Cook Islands | 0.492 | 0.705 | 0.370 | 2.771 | 3.971 | 2.087 |
| Palau | 0.471 | 0.633 | 0.341 | 2.602 | 3.499 | 1.886 |
| Sao Tome and Principe | 0.377 | 0.676 | 0.153 | 0.174 | 0.312 | 0.071 |
| Tuvalu | 0.174 | 0.236 | 0.112 | 1.404 | 1.908 | 0.904 |
| Nauru | 0.172 | 0.252 | 0.098 | 1.558 | 2.282 | 0.893 |
| Niue | 0.046 | 0.060 | 0.030 | 2.725 | 3.570 | 1.792 |
| Kiribati | 0.045 | 0.089 | 0.028 | 0.037 | 0.073 | 0.023 |
| Tokelau  Abbreviations: ASR, age-standardized rate; TC, thyroid cancer. | 0.031 | 0.041 | 0.021 | 2.259 | 3.020 | 1.511 |
