# Supplementary material for: Trends and cross-country inequalities in the global burden of thyroid cancer, 1990–2021: A population-based study
Source: Medicine (Baltimore). 2026 Aug 14;105(33):e50237. doi: 10.1097/MD.0000000000050237 (PMC13480896; doi:10.1097/MD.0000000000050237)
Supplement: Supplementary file 2 [file medi-105-e50237-s002.docx]

| Location  Table S2. The case number and ASR of prevalence of TC in 2021 by countries/territories. | Number | Number_upper | Number_lower | Rate | Rate_upper | Rate_lower |
| --- | --- | --- | --- | --- | --- | --- |
| China | 388411.040 | 488388.375 | 311967.453 | 27.300 | 34.327 | 21.927 |
| United States of America | 222227.835 | 231109.705 | 212085.423 | 66.807 | 69.477 | 63.758 |
| India | 214072.155 | 259618.314 | 170093.344 | 15.134 | 18.354 | 12.025 |
| Japan | 76047.244 | 81980.567 | 69549.509 | 59.553 | 64.199 | 54.464 |
| Viet Nam | 57626.276 | 77559.867 | 37939.063 | 57.472 | 77.353 | 37.838 |
| Indonesia | 55528.944 | 75127.056 | 35549.640 | 19.909 | 26.936 | 12.746 |
| France | 53648.395 | 64054.198 | 44286.987 | 80.808 | 96.482 | 66.707 |
| Russian Federation | 53501.527 | 58912.706 | 48453.233 | 36.935 | 40.670 | 33.450 |
| Italy | 43993.313 | 48617.281 | 39726.942 | 73.553 | 81.284 | 66.420 |
| Pakistan | 41009.444 | 58771.574 | 27812.533 | 17.410 | 24.950 | 11.807 |
| Germany | 39078.055 | 46847.025 | 31979.153 | 45.774 | 54.874 | 37.459 |
| Turkey | 36202.808 | 49450.185 | 26994.455 | 43.299 | 59.142 | 32.285 |
| Republic of Korea | 32512.390 | 49154.198 | 24249.424 | 63.045 | 95.315 | 47.022 |
| Brazil | 31849.013 | 33749.363 | 29877.374 | 14.453 | 15.316 | 13.559 |
| Thailand | 30235.270 | 46346.735 | 21490.437 | 45.342 | 69.503 | 32.228 |
| Philippines | 29795.466 | 37321.944 | 23719.823 | 26.310 | 32.955 | 20.945 |
| Mexico | 29413.886 | 33675.183 | 25547.042 | 22.753 | 26.049 | 19.762 |
| Iran (Islamic Republic of) | 28838.292 | 36157.320 | 12164.897 | 33.786 | 42.361 | 14.252 |
| Saudi Arabia | 26111.834 | 34798.888 | 18459.177 | 69.254 | 92.294 | 48.958 |
| Bangladesh | 23295.923 | 43137.225 | 14513.736 | 14.150 | 26.202 | 8.816 |
| Ethiopia | 20449.335 | 35627.607 | 13267.990 | 18.772 | 32.704 | 12.179 |
| United Kingdom | 20441.274 | 21220.104 | 19496.937 | 30.128 | 31.276 | 28.736 |
| Egypt | 17204.717 | 22821.408 | 12717.149 | 16.288 | 21.606 | 12.040 |
| Taiwan (Province of China) | 16877.901 | 19222.427 | 14499.309 | 71.406 | 81.325 | 61.342 |
| Colombia | 16167.847 | 20259.669 | 12936.370 | 32.955 | 41.295 | 26.368 |
| Canada | 15490.141 | 18782.684 | 12643.824 | 41.341 | 50.128 | 33.745 |
| Spain | 15246.473 | 18026.111 | 12802.457 | 33.472 | 39.575 | 28.107 |
| Australia | 14705.448 | 17994.631 | 11776.140 | 57.016 | 69.768 | 45.658 |
| Ukraine | 14167.128 | 20160.974 | 9646.728 | 32.887 | 46.801 | 22.394 |
| Algeria | 13921.115 | 18509.204 | 9564.732 | 31.497 | 41.878 | 21.641 |
| Iraq | 13392.328 | 18568.501 | 9601.250 | 32.486 | 45.043 | 23.290 |
| Poland | 12445.269 | 13985.634 | 10935.767 | 32.546 | 36.575 | 28.599 |
| Peru | 10300.983 | 14364.412 | 7140.804 | 28.400 | 39.603 | 19.687 |
| Malaysia | 10236.253 | 12589.878 | 8425.400 | 32.177 | 39.576 | 26.485 |
| Myanmar | 10233.483 | 13916.431 | 6724.090 | 18.138 | 24.666 | 11.918 |
| Argentina | 8653.214 | 10495.719 | 7102.674 | 19.023 | 23.074 | 15.615 |
| Morocco | 7009.966 | 10278.962 | 4684.505 | 18.856 | 27.649 | 12.601 |
| Sri Lanka | 6653.881 | 9608.844 | 3998.773 | 29.878 | 43.147 | 17.956 |
| Democratic People's Republic of Korea | 6113.468 | 8882.259 | 4082.854 | 23.164 | 33.655 | 15.470 |
| Uganda | 6079.417 | 8693.171 | 3959.484 | 14.035 | 20.070 | 9.141 |
| Chile | 5743.591 | 6879.574 | 4873.292 | 30.555 | 36.598 | 25.925 |
| United Republic of Tanzania | 5664.739 | 8907.857 | 3671.784 | 9.692 | 15.241 | 6.282 |
| Location  Table S2 (continued) | Number | Number_upper | Number_lower | Rate | Rate_upper | Rate_lower |
| Austria | 5653.147 | 6782.583 | 4669.961 | 62.936 | 75.510 | 51.991 |
| Venezuela (Bolivarian Republic of) | 5374.199 | 6996.010 | 3953.949 | 20.182 | 26.272 | 14.848 |
| Sudan | 5357.791 | 8629.761 | 3048.999 | 12.340 | 19.876 | 7.022 |
| Czechia | 5261.837 | 6879.189 | 3972.212 | 49.489 | 64.700 | 37.360 |
| Romania | 5224.928 | 6742.773 | 4023.996 | 27.589 | 35.603 | 21.248 |
| Portugal | 5219.623 | 6487.461 | 4112.351 | 49.205 | 61.157 | 38.767 |
| Ecuador | 5186.628 | 6649.142 | 3913.077 | 28.710 | 36.806 | 21.661 |
| South Africa | 5117.220 | 6498.840 | 4450.495 | 9.001 | 11.431 | 7.828 |
| Kazakhstan | 4808.069 | 5748.881 | 3902.229 | 25.365 | 30.328 | 20.586 |
| Belgium | 4668.156 | 5679.432 | 3772.762 | 40.701 | 49.519 | 32.895 |
| Tunisia | 4529.289 | 6120.567 | 3030.973 | 38.246 | 51.683 | 25.594 |
| Netherlands | 4426.179 | 5214.853 | 3699.957 | 25.718 | 30.300 | 21.498 |
| Belarus | 4397.375 | 5632.063 | 3418.855 | 47.159 | 60.400 | 36.665 |
| Cuba | 4297.624 | 5103.333 | 3546.377 | 38.135 | 45.284 | 31.469 |
| Afghanistan | 4245.902 | 7131.754 | 1868.737 | 13.599 | 22.842 | 5.985 |
| Cambodia | 4041.956 | 5840.136 | 2477.344 | 23.715 | 34.265 | 14.535 |
| Nepal | 4027.125 | 6430.641 | 2617.353 | 12.937 | 20.658 | 8.408 |
| Jordan | 3968.559 | 5553.560 | 2868.831 | 32.200 | 45.061 | 23.277 |
| Libya | 3929.329 | 5582.516 | 2675.594 | 57.191 | 81.253 | 38.943 |
| Syrian Arab Republic | 3832.443 | 6090.893 | 2348.368 | 27.315 | 43.412 | 16.738 |
| Hungary | 3571.853 | 4278.788 | 2917.484 | 37.217 | 44.583 | 30.399 |
| Greece | 3432.751 | 3895.115 | 3017.477 | 33.737 | 38.282 | 29.656 |
| United Arab Emirates | 3275.346 | 4485.542 | 2486.772 | 34.008 | 46.573 | 25.820 |
| Israel | 2809.790 | 3441.124 | 2305.477 | 29.287 | 35.868 | 24.031 |
| Yemen | 2785.395 | 4013.611 | 1868.789 | 8.279 | 11.930 | 5.555 |
| Zambia | 2712.174 | 5757.066 | 1246.094 | 13.898 | 29.500 | 6.385 |
| Bolivia (Plurinational State of) | 2559.724 | 3690.300 | 1725.502 | 21.698 | 31.281 | 14.626 |
| Kuwait | 2461.055 | 2930.258 | 1960.918 | 52.925 | 63.015 | 42.170 |
| Zimbabwe | 2381.721 | 3382.163 | 1428.545 | 15.272 | 21.687 | 9.160 |
| Mozambique | 2371.946 | 4578.600 | 1388.062 | 7.634 | 14.735 | 4.467 |
| Sweden | 2344.267 | 2838.826 | 1923.969 | 22.599 | 27.366 | 18.547 |
| Madagascar | 2288.885 | 3245.609 | 1516.198 | 8.015 | 11.365 | 5.309 |
| Serbia | 2282.832 | 3156.424 | 1623.733 | 25.594 | 35.388 | 18.204 |
| Singapore | 2261.683 | 2807.935 | 1870.224 | 39.490 | 49.027 | 32.655 |
| Lebanon | 2235.475 | 2995.784 | 1692.939 | 40.348 | 54.071 | 30.556 |
| Switzerland | 2167.823 | 2556.948 | 1809.754 | 24.295 | 28.656 | 20.282 |
| Finland | 2147.555 | 2625.923 | 1753.211 | 38.793 | 47.434 | 31.670 |
| Democratic Republic of the Congo | 2056.120 | 3447.139 | 1240.512 | 2.284 | 3.829 | 1.378 |
| Croatia | 2009.750 | 2471.351 | 1610.977 | 47.753 | 58.721 | 38.278 |
| Kenya | 1950.111 | 3125.587 | 1361.471 | 3.895 | 6.243 | 2.720 |
| Location  Table S2 (continued) | Number | Number_upper | Number_lower | Rate | Rate_upper | Rate_lower |
| Uzbekistan | 1946.341 | 2674.204 | 1420.522 | 5.686 | 7.812 | 4.150 |
| Guatemala | 1887.434 | 2228.423 | 1588.342 | 11.971 | 14.133 | 10.074 |
| Slovakia | 1845.161 | 2524.530 | 1399.492 | 33.984 | 46.497 | 25.776 |
| Bulgaria | 1828.427 | 2344.389 | 1426.793 | 26.940 | 34.543 | 21.023 |
| Norway | 1796.533 | 2012.486 | 1572.836 | 33.158 | 37.144 | 29.029 |
| Malawi | 1762.045 | 3022.181 | 1028.132 | 9.060 | 15.540 | 5.287 |
| Georgia | 1586.422 | 1979.757 | 1260.945 | 43.978 | 54.882 | 34.956 |
| Ireland | 1548.388 | 1861.827 | 1287.304 | 31.335 | 37.678 | 26.052 |
| El Salvador | 1515.475 | 1939.088 | 1165.333 | 23.495 | 30.062 | 18.067 |
| New Zealand | 1505.761 | 1776.668 | 1217.624 | 29.128 | 34.368 | 23.554 |
| Costa Rica | 1490.644 | 1740.936 | 1243.093 | 31.396 | 36.668 | 26.182 |
| Rwanda | 1396.595 | 2222.976 | 912.292 | 10.524 | 16.752 | 6.875 |
| Coted'Ivoire | 1303.987 | 1961.943 | 859.098 | 4.680 | 7.042 | 3.083 |
| Lithuania | 1295.042 | 1615.641 | 1010.766 | 47.467 | 59.218 | 37.047 |
| Dominican Republic | 1294.955 | 1761.022 | 943.564 | 11.759 | 15.991 | 8.568 |
| Paraguay | 1233.688 | 1631.917 | 897.467 | 17.209 | 22.764 | 12.519 |
| Kyrgyzstan | 1223.848 | 1674.891 | 856.358 | 17.832 | 24.404 | 12.478 |
| Mali | 1216.203 | 1906.532 | 780.728 | 5.046 | 7.910 | 3.239 |
| Qatar | 1210.761 | 1675.360 | 852.396 | 40.670 | 56.276 | 28.632 |
| Azerbaijan | 1195.958 | 1719.538 | 806.570 | 11.390 | 16.376 | 7.681 |
| Palestine | 1191.442 | 1507.949 | 776.013 | 23.200 | 29.363 | 15.111 |
| Denmark | 1130.622 | 1363.016 | 942.948 | 19.321 | 23.292 | 16.114 |
| Armenia | 1117.738 | 1476.234 | 836.775 | 37.317 | 49.285 | 27.936 |
| Lao People's Democratic Republic | 1113.150 | 1567.238 | 708.024 | 15.088 | 21.243 | 9.597 |
| Uruguay | 1102.653 | 1354.299 | 905.468 | 32.377 | 39.767 | 26.587 |
| Puerto Rico | 1044.910 | 1284.303 | 837.139 | 31.718 | 38.985 | 25.411 |
| Somalia | 1002.258 | 1519.242 | 610.817 | 4.639 | 7.031 | 2.827 |
| Republic of Moldova | 986.548 | 1172.855 | 835.224 | 27.454 | 32.639 | 23.243 |
| Panama | 972.238 | 1220.646 | 771.577 | 22.652 | 28.440 | 17.977 |
| Latvia | 935.600 | 1222.568 | 705.953 | 50.023 | 65.366 | 37.745 |
| Oman | 911.146 | 1227.314 | 606.815 | 19.371 | 26.092 | 12.901 |
| Nigeria | 905.740 | 1581.707 | 546.243 | 0.392 | 0.684 | 0.236 |
| Haiti | 847.670 | 1253.475 | 561.681 | 6.589 | 9.744 | 4.366 |
| Burundi | 845.924 | 1385.819 | 528.269 | 6.399 | 10.483 | 3.996 |
| Angola | 844.887 | 1396.809 | 494.952 | 2.583 | 4.271 | 1.513 |
| Bosnia and Herzegovina | 794.580 | 1076.726 | 578.260 | 24.057 | 32.600 | 17.508 |
| Nicaragua | 785.534 | 1002.206 | 581.835 | 11.779 | 15.028 | 8.724 |
| South Sudan | 771.297 | 1257.343 | 486.310 | 7.974 | 12.999 | 5.028 |
| Bahrain | 705.082 | 958.580 | 520.889 | 46.096 | 62.669 | 34.054 |
| Turkmenistan | 688.270 | 930.767 | 517.995 | 13.343 | 18.044 | 10.042 |
| Slovenia | 677.820 | 911.251 | 490.913 | 32.750 | 44.029 | 23.720 |
| Estonia | 623.191 | 794.569 | 471.478 | 47.542 | 60.616 | 35.968 |
| Location  Table S2 (continued) | Number | Number_upper | Number_lower | Rate | Rate_upper | Rate_lower |
| Jamaica | 612.322 | 837.862 | 439.068 | 21.869 | 29.925 | 15.682 |
| Albania | 590.283 | 870.382 | 398.696 | 22.126 | 32.625 | 14.944 |
| Eritrea | 585.819 | 958.695 | 366.187 | 8.879 | 14.530 | 5.550 |
| Cameroon | 577.273 | 868.547 | 353.036 | 1.816 | 2.733 | 1.111 |
| North Macedonia | 538.948 | 695.337 | 404.553 | 24.763 | 31.948 | 18.588 |
| Papua New Guinea | 484.683 | 886.210 | 252.737 | 4.633 | 8.471 | 2.416 |
| Honduras | 463.781 | 652.889 | 309.403 | 4.587 | 6.458 | 3.060 |
| Mongolia | 446.292 | 622.010 | 328.512 | 13.377 | 18.644 | 9.847 |
| Cyprus | 419.778 | 591.067 | 312.019 | 30.917 | 43.532 | 22.980 |
| Guinea | 358.614 | 550.317 | 241.185 | 2.670 | 4.098 | 1.796 |
| Trinidad and Tobago | 336.991 | 443.481 | 253.241 | 24.189 | 31.833 | 18.178 |
| Burkina Faso | 291.794 | 418.524 | 171.427 | 1.282 | 1.839 | 0.753 |
| Montenegro | 268.970 | 341.731 | 208.882 | 43.518 | 55.291 | 33.796 |
| Luxembourg | 261.693 | 305.596 | 227.355 | 40.619 | 47.433 | 35.289 |
| Congo | 261.370 | 427.224 | 154.241 | 4.848 | 7.924 | 2.861 |
| Iceland | 251.215 | 311.469 | 205.399 | 71.697 | 88.893 | 58.621 |
| Fiji | 243.968 | 348.004 | 145.120 | 26.390 | 37.643 | 15.698 |
| Senegal | 243.861 | 398.571 | 152.322 | 1.538 | 2.513 | 0.960 |
| Mauritius | 217.980 | 242.138 | 190.771 | 17.137 | 19.037 | 14.998 |
| Niger | 187.010 | 289.953 | 111.552 | 0.747 | 1.158 | 0.446 |
| Namibia | 182.930 | 273.202 | 115.347 | 7.524 | 11.237 | 4.745 |
| Malta | 179.764 | 220.945 | 144.124 | 40.650 | 49.963 | 32.591 |
| Ghana | 176.469 | 270.716 | 108.642 | 0.515 | 0.791 | 0.317 |
| Chad | 161.663 | 229.862 | 105.754 | 0.911 | 1.295 | 0.596 |
| Benin | 156.186 | 247.093 | 94.433 | 1.157 | 1.830 | 0.700 |
| Togo | 145.519 | 235.160 | 84.253 | 1.738 | 2.809 | 1.006 |
| Brunei Darussalam | 137.820 | 186.084 | 102.506 | 30.549 | 41.248 | 22.722 |
| Botswana | 137.502 | 235.912 | 83.272 | 5.745 | 9.857 | 3.479 |
| Timor-Leste | 132.626 | 195.172 | 83.762 | 9.489 | 13.963 | 5.993 |
| Djibouti | 126.954 | 235.855 | 71.143 | 10.086 | 18.738 | 5.652 |
| Sierra Leone | 119.967 | 184.659 | 68.391 | 1.353 | 2.082 | 0.771 |
| Bahamas | 107.442 | 138.297 | 83.760 | 27.694 | 35.647 | 21.590 |
| Bhutan | 104.478 | 187.062 | 61.736 | 13.803 | 24.714 | 8.156 |
| Gabon | 102.891 | 160.718 | 64.596 | 5.666 | 8.850 | 3.557 |
| Central African Republic | 101.413 | 160.519 | 63.079 | 1.849 | 2.927 | 1.150 |
| Liberia | 100.023 | 145.557 | 61.522 | 1.832 | 2.666 | 1.127 |
| Lesotho | 99.246 | 158.724 | 64.100 | 5.295 | 8.468 | 3.420 |
| Guyana | 90.278 | 123.447 | 66.513 | 11.805 | 16.142 | 8.698 |
| Barbados | 88.461 | 113.945 | 68.673 | 29.583 | 38.105 | 22.966 |
| Comoros | 87.625 | 147.500 | 53.262 | 11.772 | 19.815 | 7.155 |
| Eswatini | 81.952 | 131.487 | 46.900 | 7.094 | 11.382 | 4.060 |
| Mauritania | 79.639 | 121.572 | 52.025 | 1.812 | 2.765 | 1.183 |
| Suriname | 69.995 | 89.517 | 50.952 | 12.084 | 15.454 | 8.796 |
| Location  Table S2 (continued) | Number | Number_upper | Number_lower | Rate | Rate_upper | Rate_lower |
| Equatorial Guinea | 65.307 | 110.615 | 33.819 | 4.318 | 7.314 | 2.236 |
| Gambia | 58.850 | 85.435 | 37.207 | 2.458 | 3.568 | 1.554 |
| Saint Lucia | 54.378 | 67.215 | 43.433 | 30.634 | 37.865 | 24.468 |
| Maldives | 50.769 | 70.879 | 37.462 | 9.816 | 13.704 | 7.243 |
| Samoa | 44.698 | 62.677 | 31.296 | 20.922 | 29.338 | 14.649 |
| Solomon Islands | 41.854 | 64.172 | 24.985 | 6.123 | 9.388 | 3.655 |
| Belize | 40.646 | 46.486 | 35.034 | 9.473 | 10.834 | 8.165 |
| Saint Vincent and the Grenadines | 39.073 | 45.736 | 33.214 | 34.252 | 40.093 | 29.116 |
| Bermuda | 37.212 | 47.153 | 29.579 | 58.563 | 74.209 | 46.551 |
| Andorra | 34.462 | 48.854 | 22.638 | 40.262 | 57.076 | 26.448 |
| Guinea-Bissau | 33.613 | 48.281 | 22.555 | 1.629 | 2.339 | 1.093 |
| Guam | 27.755 | 32.834 | 20.856 | 17.434 | 20.625 | 13.101 |
| Cabo Verde | 26.724 | 51.889 | 5.194 | 4.778 | 9.278 | 0.929 |
| Antigua and Barbuda | 26.235 | 29.077 | 23.789 | 29.348 | 32.527 | 26.611 |
| Grenada | 24.910 | 30.268 | 20.382 | 24.272 | 29.494 | 19.860 |
| Monaco | 20.946 | 27.549 | 15.080 | 55.320 | 72.758 | 39.826 |
| Vanuatu | 18.143 | 24.907 | 12.031 | 5.797 | 7.958 | 3.844 |
| San Marino | 17.824 | 26.383 | 10.258 | 54.441 | 80.583 | 31.332 |
| American Samoa | 13.515 | 17.814 | 9.446 | 27.161 | 35.801 | 18.983 |
| Seychelles | 11.047 | 13.608 | 8.893 | 10.478 | 12.907 | 8.435 |
| Greenland | 10.338 | 14.525 | 6.958 | 18.426 | 25.890 | 12.403 |
| Micronesia (Federated States of) | 10.283 | 14.480 | 6.732 | 10.025 | 14.116 | 6.562 |
| United States Virgin Islands | 9.382 | 12.964 | 6.712 | 10.921 | 15.092 | 7.813 |
| Tonga | 9.358 | 13.659 | 5.794 | 8.803 | 12.850 | 5.451 |
| Saint Kitts and Nevis | 9.173 | 11.299 | 7.365 | 15.641 | 19.267 | 12.558 |
| Dominica | 9.000 | 11.576 | 6.838 | 13.418 | 17.259 | 10.194 |
| Tajikistan | 8.392 | 12.247 | 5.660 | 0.083 | 0.121 | 0.056 |
| Northern Mariana Islands | 7.525 | 9.173 | 5.648 | 15.519 | 18.916 | 11.648 |
| Marshall Islands | 4.867 | 7.306 | 3.099 | 8.650 | 12.984 | 5.507 |
| Cook Islands | 3.663 | 5.317 | 2.689 | 20.641 | 29.962 | 15.153 |
| Palau | 3.462 | 4.826 | 2.495 | 19.132 | 26.666 | 13.788 |
| Sao Tome and Principe | 3.117 | 5.764 | 1.152 | 1.439 | 2.661 | 0.532 |
| Nauru | 1.276 | 1.890 | 0.721 | 11.574 | 17.142 | 6.542 |
| Tuvalu | 1.208 | 1.668 | 0.770 | 9.766 | 13.483 | 6.225 |
| Niue | 0.326 | 0.436 | 0.213 | 19.503 | 26.107 | 12.726 |
| Kiribati | 0.288 | 0.584 | 0.171 | 0.238 | 0.482 | 0.141 |
| Tokelau  Abbreviations: ASR, age-standardized rate; TC, thyroid cancer. | 0.226 | 0.305 | 0.151 | 16.506 | 22.272 | 10.994 |
