# Supplementary material for: Trends and cross-country inequalities in the global burden of thyroid cancer, 1990–2021: A population-based study
Source: Medicine (Baltimore). 2026 Aug 14;105(33):e50237. doi: 10.1097/MD.0000000000050237 (PMC13480896; doi:10.1097/MD.0000000000050237)
Supplement: Supplementary file 3 [file medi-105-e50237-s003.docx]

| Location  Table S3. The case number and ASR of deaths of TC in 2021 by countries/territories. | Number | Number_upper | Number_lower | Rate | Rate_upper | Rate_lower |
| --- | --- | --- | --- | --- | --- | --- |
| China | 7692.208 | 9428.762 | 6122.516 | 0.541 | 0.663 | 0.430 |
| India | 7156.153 | 8237.231 | 5975.872 | 0.506 | 0.582 | 0.422 |
| United States of America | 2495.153 | 2640.731 | 2237.101 | 0.750 | 0.794 | 0.673 |
| Japan | 2292.315 | 2571.511 | 1815.650 | 1.795 | 2.014 | 1.422 |
| Indonesia | 1844.504 | 2420.950 | 1231.148 | 0.661 | 0.868 | 0.441 |
| Pakistan | 1342.921 | 1751.924 | 1014.746 | 0.570 | 0.744 | 0.431 |
| Brazil | 1206.333 | 1281.457 | 1100.604 | 0.547 | 0.582 | 0.499 |
| Russian Federation | 1142.389 | 1243.639 | 1049.881 | 0.789 | 0.859 | 0.725 |
| Viet Nam | 1085.096 | 1422.195 | 816.814 | 1.082 | 1.418 | 0.815 |
| Mexico | 1054.187 | 1177.480 | 936.004 | 0.815 | 0.911 | 0.724 |
| Philippines | 1018.837 | 1226.871 | 827.067 | 0.900 | 1.083 | 0.730 |
| Germany | 860.782 | 1013.877 | 716.321 | 1.008 | 1.188 | 0.839 |
| Ethiopia | 753.569 | 1130.256 | 510.582 | 0.692 | 1.038 | 0.469 |
| Thailand | 689.352 | 966.700 | 512.332 | 1.034 | 1.450 | 0.768 |
| Bangladesh | 683.832 | 1075.030 | 451.609 | 0.415 | 0.653 | 0.274 |
| Italy | 652.656 | 723.118 | 556.728 | 1.091 | 1.209 | 0.931 |
| France | 527.557 | 630.331 | 423.119 | 0.795 | 0.949 | 0.637 |
| Republic of Korea | 512.297 | 689.201 | 407.131 | 0.993 | 1.336 | 0.789 |
| United Kingdom | 478.261 | 504.141 | 430.108 | 0.705 | 0.743 | 0.634 |
| Turkey | 476.544 | 602.880 | 376.274 | 0.570 | 0.721 | 0.450 |
| Colombia | 467.518 | 572.927 | 383.825 | 0.953 | 1.168 | 0.782 |
| Myanmar | 402.338 | 531.131 | 281.856 | 0.713 | 0.941 | 0.500 |
| Spain | 366.079 | 440.908 | 298.833 | 0.804 | 0.968 | 0.656 |
| Poland | 347.613 | 381.060 | 310.852 | 0.909 | 0.997 | 0.813 |
| Ukraine | 325.860 | 438.059 | 235.973 | 0.756 | 1.017 | 0.548 |
| Argentina | 305.739 | 359.671 | 255.127 | 0.672 | 0.791 | 0.561 |
| Peru | 298.233 | 400.559 | 204.999 | 0.822 | 1.104 | 0.565 |
| Canada | 270.265 | 324.085 | 224.817 | 0.721 | 0.865 | 0.600 |
| Egypt | 252.609 | 314.714 | 196.345 | 0.239 | 0.298 | 0.186 |
| Iran (Islamic Republic of) | 230.028 | 278.580 | 104.197 | 0.269 | 0.326 | 0.122 |
| Malaysia | 229.393 | 287.392 | 195.115 | 0.721 | 0.903 | 0.613 |
| Taiwan (Province of China) | 222.434 | 247.174 | 191.024 | 0.941 | 1.046 | 0.808 |
| Uganda | 218.230 | 292.111 | 155.203 | 0.504 | 0.674 | 0.358 |
| United Republic of Tanzania | 208.978 | 289.590 | 148.510 | 0.358 | 0.495 | 0.254 |
| Ecuador | 204.649 | 253.779 | 160.628 | 1.133 | 1.405 | 0.889 |
| South Africa | 200.885 | 227.063 | 164.759 | 0.353 | 0.399 | 0.290 |
| Venezuela (Bolivarian Republic of) | 195.653 | 253.069 | 150.267 | 0.735 | 0.950 | 0.564 |
| Australia | 170.279 | 206.462 | 135.825 | 0.660 | 0.800 | 0.527 |
| Cambodia | 153.716 | 209.899 | 98.569 | 0.902 | 1.232 | 0.578 |
| Location  Table(continued) | Number | Number_upper | Number_lower | Rate | Rate_upper | Rate_lower |
| Sri Lanka | 152.085 | 214.317 | 97.950 | 0.683 | 0.962 | 0.440 |
| Romania | 151.508 | 186.452 | 121.179 | 0.800 | 0.985 | 0.640 |
| Democratic People's Republic of Korea | 149.125 | 202.857 | 107.198 | 0.565 | 0.769 | 0.406 |
| Saudi Arabia | 144.693 | 184.258 | 110.844 | 0.384 | 0.489 | 0.294 |
| Chile | 139.600 | 165.977 | 119.088 | 0.743 | 0.883 | 0.634 |
| Bolivia (Plurinational State of) | 138.271 | 191.904 | 90.164 | 1.172 | 1.627 | 0.764 |
| Netherlands | 137.852 | 157.619 | 113.659 | 0.801 | 0.916 | 0.660 |
| Nepal | 137.257 | 193.636 | 97.784 | 0.441 | 0.622 | 0.314 |
| Algeria | 135.963 | 171.181 | 102.296 | 0.308 | 0.387 | 0.231 |
| Iraq | 131.213 | 170.839 | 95.757 | 0.318 | 0.414 | 0.232 |
| Cuba | 127.621 | 148.476 | 107.214 | 1.132 | 1.317 | 0.951 |
| Democratic Republic of the Congo | 116.249 | 189.650 | 70.177 | 0.129 | 0.211 | 0.078 |
| Morocco | 110.238 | 140.394 | 82.742 | 0.297 | 0.378 | 0.223 |
| Belgium | 108.023 | 130.974 | 87.298 | 0.942 | 1.142 | 0.761 |
| Mozambique | 106.048 | 177.935 | 68.794 | 0.341 | 0.573 | 0.221 |
| Kazakhstan | 105.255 | 125.083 | 86.605 | 0.555 | 0.660 | 0.457 |
| Greece | 99.855 | 110.462 | 86.770 | 0.981 | 1.086 | 0.853 |
| Zimbabwe | 99.070 | 128.871 | 70.050 | 0.635 | 0.826 | 0.449 |
| Austria | 98.378 | 117.195 | 81.660 | 1.095 | 1.305 | 0.909 |
| Portugal | 97.165 | 118.152 | 78.979 | 0.916 | 1.114 | 0.745 |
| Zambia | 92.591 | 166.708 | 46.793 | 0.474 | 0.854 | 0.240 |
| Sweden | 92.011 | 109.781 | 73.873 | 0.887 | 1.058 | 0.712 |
| Belarus | 83.617 | 106.549 | 65.823 | 0.897 | 1.143 | 0.706 |
| Czechia | 82.983 | 102.076 | 66.316 | 0.780 | 0.960 | 0.624 |
| Hungary | 81.889 | 100.464 | 66.794 | 0.853 | 1.047 | 0.696 |
| Afghanistan | 80.588 | 132.069 | 38.959 | 0.258 | 0.423 | 0.125 |
| Guatemala | 79.733 | 91.657 | 68.686 | 0.506 | 0.581 | 0.436 |
| Madagascar | 79.366 | 107.334 | 54.925 | 0.278 | 0.376 | 0.192 |
| Serbia | 77.554 | 102.392 | 57.038 | 0.869 | 1.148 | 0.639 |
| Sudan | 71.740 | 103.829 | 48.248 | 0.165 | 0.239 | 0.111 |
| Israel | 68.404 | 81.874 | 56.842 | 0.713 | 0.853 | 0.592 |
| Bulgaria | 66.699 | 84.587 | 52.966 | 0.983 | 1.246 | 0.780 |
| Kenya | 65.910 | 97.278 | 48.825 | 0.132 | 0.194 | 0.098 |
| Malawi | 61.288 | 89.173 | 40.583 | 0.315 | 0.459 | 0.209 |
| Switzerland | 59.994 | 71.537 | 49.346 | 0.672 | 0.802 | 0.553 |
| Somalia | 54.827 | 76.751 | 35.650 | 0.254 | 0.355 | 0.165 |
| Uzbekistan | 53.532 | 71.439 | 39.967 | 0.156 | 0.209 | 0.117 |
| Rwanda | 53.146 | 75.391 | 37.612 | 0.400 | 0.568 | 0.283 |
| Georgia | 52.959 | 65.137 | 42.577 | 1.468 | 1.806 | 1.180 |
| Haiti | 52.842 | 72.125 | 37.136 | 0.411 | 0.561 | 0.289 |
| Location  Table(continued) | Number | Number_upper | Number_lower | Rate | Rate_upper | Rate_lower |
| Yemen | 50.333 | 73.338 | 34.225 | 0.150 | 0.218 | 0.102 |
| El Salvador | 49.340 | 61.037 | 38.694 | 0.765 | 0.946 | 0.600 |
| Dominican Republic | 48.345 | 62.570 | 36.489 | 0.439 | 0.568 | 0.331 |
| Finland | 48.326 | 58.400 | 38.762 | 0.873 | 1.055 | 0.700 |
| Slovakia | 46.671 | 60.421 | 37.304 | 0.860 | 1.113 | 0.687 |
| Paraguay | 46.459 | 59.869 | 33.718 | 0.648 | 0.835 | 0.470 |
| Tunisia | 45.876 | 64.197 | 32.252 | 0.387 | 0.542 | 0.272 |
| Lao People's Democratic Republic | 45.418 | 62.250 | 29.762 | 0.616 | 0.844 | 0.403 |
| Mali | 43.977 | 62.555 | 31.551 | 0.182 | 0.260 | 0.131 |
| Norway | 43.841 | 48.607 | 37.424 | 0.809 | 0.897 | 0.691 |
| Syrian Arab Republic | 42.908 | 64.366 | 28.930 | 0.306 | 0.459 | 0.206 |
| Angola | 40.556 | 64.242 | 24.833 | 0.124 | 0.196 | 0.076 |
| Uruguay | 39.883 | 47.655 | 33.352 | 1.171 | 1.399 | 0.979 |
| Costa Rica | 38.407 | 44.257 | 32.928 | 0.809 | 0.932 | 0.694 |
| Burundi | 37.596 | 55.761 | 24.608 | 0.284 | 0.422 | 0.186 |
| Croatia | 37.502 | 43.899 | 32.100 | 0.891 | 1.043 | 0.763 |
| Singapore | 36.503 | 44.273 | 29.898 | 0.637 | 0.773 | 0.522 |
| Coted'Ivoire | 35.399 | 48.614 | 25.296 | 0.127 | 0.174 | 0.091 |
| Denmark | 35.182 | 41.733 | 29.318 | 0.601 | 0.713 | 0.501 |
| Armenia | 34.908 | 44.996 | 26.708 | 1.165 | 1.502 | 0.892 |
| Azerbaijan | 33.965 | 43.659 | 25.098 | 0.323 | 0.416 | 0.239 |
| Jordan | 32.379 | 43.625 | 24.332 | 0.263 | 0.354 | 0.197 |
| South Sudan | 32.338 | 46.731 | 22.316 | 0.334 | 0.483 | 0.231 |
| Libya | 31.776 | 41.994 | 23.894 | 0.462 | 0.611 | 0.348 |
| Lithuania | 30.800 | 37.454 | 24.762 | 1.129 | 1.373 | 0.908 |
| Panama | 29.399 | 36.104 | 23.234 | 0.685 | 0.841 | 0.541 |
| New Zealand | 28.811 | 34.208 | 23.362 | 0.557 | 0.662 | 0.452 |
| Puerto Rico | 28.075 | 33.451 | 22.986 | 0.852 | 1.015 | 0.698 |
| United Arab Emirates | 28.002 | 36.057 | 21.836 | 0.291 | 0.374 | 0.227 |
| Latvia | 27.686 | 34.878 | 21.329 | 1.480 | 1.865 | 1.140 |
| Nigeria | 27.679 | 50.309 | 18.436 | 0.012 | 0.022 | 0.008 |
| Ireland | 26.886 | 32.157 | 21.831 | 0.544 | 0.651 | 0.442 |
| Eritrea | 26.780 | 39.006 | 18.682 | 0.406 | 0.591 | 0.283 |
| Kyrgyzstan | 26.375 | 33.699 | 20.210 | 0.384 | 0.491 | 0.294 |
| Lebanon | 25.320 | 32.144 | 20.240 | 0.457 | 0.580 | 0.365 |
| Honduras | 25.272 | 34.488 | 17.877 | 0.250 | 0.341 | 0.177 |
| Nicaragua | 25.045 | 31.210 | 18.649 | 0.376 | 0.468 | 0.280 |
| Republic of Moldova | 24.673 | 29.315 | 20.790 | 0.687 | 0.816 | 0.579 |
| Bosnia and Herzegovina | 23.919 | 30.622 | 17.805 | 0.724 | 0.927 | 0.539 |
| Jamaica | 19.959 | 25.776 | 15.167 | 0.713 | 0.921 | 0.542 |
| Papua New Guinea | 19.400 | 32.137 | 10.781 | 0.185 | 0.307 | 0.103 |
| Turkmenistan | 19.013 | 24.639 | 14.691 | 0.369 | 0.478 | 0.285 |
| Location  Table(continued) | Number | Number_upper | Number_lower | Rate | Rate_upper | Rate_lower |
| Albania | 18.614 | 25.837 | 13.408 | 0.698 | 0.968 | 0.503 |
| Cameroon | 18.420 | 26.446 | 12.580 | 0.058 | 0.083 | 0.040 |
| Guinea | 17.299 | 23.501 | 12.463 | 0.129 | 0.175 | 0.093 |
| North Macedonia | 15.559 | 19.279 | 12.355 | 0.715 | 0.886 | 0.568 |
| Estonia | 15.484 | 19.875 | 11.768 | 1.181 | 1.516 | 0.898 |
| Slovenia | 14.697 | 19.109 | 10.738 | 0.710 | 0.923 | 0.519 |
| Palestine | 14.092 | 17.424 | 8.680 | 0.274 | 0.339 | 0.169 |
| Mongolia | 13.039 | 17.119 | 10.034 | 0.391 | 0.513 | 0.301 |
| Kuwait | 12.402 | 14.859 | 10.195 | 0.267 | 0.320 | 0.219 |
| Burkina Faso | 11.582 | 15.587 | 7.761 | 0.051 | 0.068 | 0.034 |
| Trinidad and Tobago | 11.524 | 14.723 | 8.871 | 0.827 | 1.057 | 0.637 |
| Congo | 11.421 | 16.933 | 7.449 | 0.212 | 0.314 | 0.138 |
| Fiji | 9.361 | 12.787 | 6.031 | 1.013 | 1.383 | 0.652 |
| Cyprus | 8.884 | 11.429 | 6.814 | 0.654 | 0.842 | 0.502 |
| Senegal | 8.775 | 13.059 | 6.050 | 0.055 | 0.082 | 0.038 |
| Niger | 8.312 | 11.848 | 5.515 | 0.033 | 0.047 | 0.022 |
| Ghana | 8.287 | 11.418 | 5.099 | 0.024 | 0.033 | 0.015 |
| Central African Republic | 8.059 | 12.026 | 5.364 | 0.147 | 0.219 | 0.098 |
| Chad | 7.083 | 9.236 | 5.145 | 0.040 | 0.052 | 0.029 |
| Lesotho | 7.014 | 10.242 | 4.858 | 0.374 | 0.546 | 0.259 |
| Timor-Leste | 6.704 | 9.598 | 4.332 | 0.480 | 0.687 | 0.310 |
| Namibia | 6.481 | 8.788 | 4.570 | 0.267 | 0.361 | 0.188 |
| Botswana | 6.285 | 9.629 | 4.133 | 0.263 | 0.402 | 0.173 |
| Mauritius | 6.267 | 6.815 | 5.660 | 0.493 | 0.536 | 0.445 |
| Montenegro | 6.117 | 7.548 | 4.932 | 0.990 | 1.221 | 0.798 |
| Oman | 5.834 | 7.398 | 4.024 | 0.124 | 0.157 | 0.086 |
| Benin | 5.683 | 8.108 | 3.918 | 0.042 | 0.060 | 0.029 |
| Luxembourg | 5.598 | 6.451 | 4.881 | 0.869 | 1.001 | 0.758 |
| Qatar | 5.536 | 7.450 | 3.937 | 0.186 | 0.250 | 0.132 |
| Bahrain | 5.304 | 6.966 | 3.760 | 0.347 | 0.455 | 0.246 |
| Djibouti | 4.817 | 7.692 | 3.069 | 0.383 | 0.611 | 0.244 |
| Togo | 4.666 | 6.988 | 3.042 | 0.056 | 0.083 | 0.036 |
| Gabon | 4.476 | 6.421 | 3.113 | 0.246 | 0.354 | 0.171 |
| Iceland | 4.361 | 5.236 | 3.563 | 1.245 | 1.494 | 1.017 |
| Guyana | 4.261 | 5.597 | 3.232 | 0.557 | 0.732 | 0.423 |
| Malta | 4.240 | 5.092 | 3.471 | 0.959 | 1.152 | 0.785 |
| Sierra Leone | 4.193 | 5.856 | 2.685 | 0.047 | 0.066 | 0.030 |
| Eswatini | 4.025 | 5.975 | 2.559 | 0.348 | 0.517 | 0.222 |
| Comoros | 3.821 | 5.899 | 2.562 | 0.513 | 0.793 | 0.344 |
| Bhutan | 3.618 | 5.347 | 2.505 | 0.478 | 0.706 | 0.331 |
| Barbados | 3.413 | 4.156 | 2.723 | 1.141 | 1.390 | 0.911 |
| Bahamas | 3.002 | 3.720 | 2.438 | 0.774 | 0.959 | 0.628 |
| Suriname | 2.958 | 3.869 | 2.179 | 0.511 | 0.668 | 0.376 |
| Location  Table(continued) | Number | Number_upper | Number_lower | Rate | Rate_upper | Rate_lower |
| Brunei Darussalam | 2.823 | 3.567 | 2.201 | 0.626 | 0.791 | 0.488 |
| Liberia | 2.783 | 3.865 | 1.889 | 0.051 | 0.071 | 0.035 |
| Mauritania | 2.439 | 3.517 | 1.705 | 0.055 | 0.080 | 0.039 |
| Equatorial Guinea | 2.048 | 3.233 | 1.177 | 0.135 | 0.214 | 0.078 |
| Gambia | 1.899 | 2.530 | 1.364 | 0.079 | 0.106 | 0.057 |
| Saint Lucia | 1.870 | 2.233 | 1.545 | 1.053 | 1.258 | 0.870 |
| Solomon Islands | 1.687 | 2.499 | 1.071 | 0.247 | 0.366 | 0.157 |
| Saint Vincent and the Grenadines | 1.509 | 1.719 | 1.315 | 1.323 | 1.506 | 1.152 |
| Guinea-Bissau | 1.268 | 1.713 | 0.919 | 0.061 | 0.083 | 0.045 |
| Belize | 1.172 | 1.323 | 1.032 | 0.273 | 0.308 | 0.241 |
| Samoa | 1.072 | 1.717 | 0.778 | 0.502 | 0.804 | 0.364 |
| Maldives | 0.989 | 1.245 | 0.750 | 0.191 | 0.241 | 0.145 |
| Grenada | 0.931 | 1.083 | 0.798 | 0.907 | 1.055 | 0.778 |
| Bermuda | 0.909 | 1.135 | 0.745 | 1.430 | 1.786 | 1.173 |
| Vanuatu | 0.822 | 1.085 | 0.554 | 0.263 | 0.347 | 0.177 |
| Antigua and Barbuda | 0.798 | 0.848 | 0.743 | 0.893 | 0.949 | 0.831 |
| Guam | 0.652 | 0.770 | 0.486 | 0.410 | 0.484 | 0.306 |
| Andorra | 0.564 | 0.781 | 0.398 | 0.659 | 0.912 | 0.466 |
| Cabo Verde | 0.532 | 1.003 | 0.142 | 0.095 | 0.179 | 0.025 |
| Monaco | 0.509 | 0.644 | 0.391 | 1.345 | 1.700 | 1.033 |
| Dominica | 0.490 | 0.595 | 0.393 | 0.731 | 0.887 | 0.586 |
| American Samoa | 0.465 | 0.605 | 0.304 | 0.934 | 1.216 | 0.611 |
| Micronesia (Federated States of) | 0.422 | 0.577 | 0.277 | 0.411 | 0.562 | 0.270 |
| San Marino | 0.410 | 0.569 | 0.271 | 1.251 | 1.737 | 0.828 |
| Saint Kitts and Nevis | 0.373 | 0.446 | 0.306 | 0.635 | 0.760 | 0.522 |
| United States Virgin Islands | 0.363 | 0.475 | 0.273 | 0.422 | 0.553 | 0.318 |
| Tonga | 0.362 | 0.520 | 0.232 | 0.340 | 0.489 | 0.218 |
| Seychelles | 0.315 | 0.376 | 0.250 | 0.299 | 0.357 | 0.237 |
| Greenland | 0.283 | 0.380 | 0.188 | 0.505 | 0.677 | 0.335 |
| Tajikistan | 0.261 | 0.361 | 0.180 | 0.003 | 0.004 | 0.002 |
| Northern Mariana Islands | 0.193 | 0.225 | 0.148 | 0.398 | 0.465 | 0.305 |
| Marshall Islands | 0.188 | 0.260 | 0.124 | 0.334 | 0.463 | 0.221 |
| Palau | 0.123 | 0.158 | 0.088 | 0.681 | 0.874 | 0.485 |
| Cook Islands | 0.112 | 0.155 | 0.086 | 0.633 | 0.875 | 0.483 |
| Sao Tome and Principe | 0.079 | 0.124 | 0.043 | 0.036 | 0.057 | 0.020 |
| Tuvalu | 0.056 | 0.073 | 0.037 | 0.449 | 0.590 | 0.298 |
| Nauru | 0.043 | 0.062 | 0.026 | 0.392 | 0.564 | 0.240 |
| Kiribati | 0.018 | 0.034 | 0.012 | 0.015 | 0.028 | 0.010 |
| Niue | 0.013 | 0.016 | 0.009 | 0.768 | 0.976 | 0.534 |
| Tokelau  Abbreviations: ASR, age-standardized rate; TC, thyroid cancer. | 0.008 | 0.011 | 0.005 | 0.602 | 0.793 | 0.396 |
