# Supplementary material for: Trends and cross-country inequalities in the global burden of thyroid cancer, 1990–2021: A population-based study
Source: Medicine (Baltimore). 2026 Aug 14;105(33):e50237. doi: 10.1097/MD.0000000000050237 (PMC13480896; doi:10.1097/MD.0000000000050237)
Supplement: Supplementary file 4 [file medi-105-e50237-s004.docx]

| Location  Table S4. The case number and ASR of DALYs of TC in 2021 by countries/territories. | Number | Number_upper | Number_lower | Rate | Rate_upper | Rate_lower |
| --- | --- | --- | --- | --- | --- | --- |
| India | 225287.796 | 264478.459 | 186616.854 | 15.927 | 18.698 | 13.193 |
| China | 203324.824 | 251788.932 | 163131.415 | 14.291 | 17.697 | 11.466 |
| United States of America | 64476.765 | 69967.158 | 59384.219 | 19.383 | 21.034 | 17.852 |
| Indonesia | 55406.557 | 72736.224 | 36365.965 | 19.865 | 26.078 | 13.038 |
| Pakistan | 50301.593 | 67210.109 | 36508.686 | 21.355 | 28.533 | 15.499 |
| Japan | 38321.112 | 42214.310 | 32739.864 | 30.009 | 33.058 | 25.639 |
| Viet Nam | 32388.042 | 42160.087 | 23960.572 | 32.302 | 42.047 | 23.897 |
| Brazil | 31441.619 | 33410.472 | 29326.928 | 14.269 | 15.162 | 13.309 |
| Philippines | 30428.269 | 36936.956 | 24389.313 | 26.868 | 32.616 | 21.536 |
| Russian Federation | 28639.193 | 31382.454 | 26154.122 | 19.771 | 21.665 | 18.056 |
| Mexico | 28243.317 | 31948.613 | 25014.255 | 21.847 | 24.713 | 19.349 |
| Ethiopia | 27067.457 | 42991.643 | 18221.826 | 24.847 | 39.464 | 16.727 |
| Bangladesh | 22091.508 | 37366.377 | 13982.003 | 13.418 | 22.696 | 8.493 |
| Germany | 18569.375 | 22064.746 | 15499.982 | 21.751 | 25.845 | 18.156 |
| Thailand | 17719.950 | 26321.349 | 13111.003 | 26.573 | 39.472 | 19.662 |
| Italy | 14512.631 | 16259.980 | 12904.004 | 24.264 | 27.185 | 21.574 |
| Turkey | 13413.772 | 17274.957 | 10490.465 | 16.043 | 20.661 | 12.547 |
| France | 12915.455 | 15389.689 | 10513.480 | 19.454 | 23.181 | 15.836 |
| Colombia | 12031.981 | 14969.100 | 9855.646 | 24.525 | 30.512 | 20.089 |
| Republic of Korea | 11531.953 | 16858.426 | 9174.975 | 22.362 | 32.690 | 17.791 |
| Myanmar | 11496.053 | 15296.143 | 7886.343 | 20.376 | 27.111 | 13.978 |
| United Kingdom | 10527.235 | 11129.771 | 9827.849 | 15.516 | 16.404 | 14.485 |
| Ukraine | 8961.479 | 12155.124 | 6370.282 | 20.803 | 28.217 | 14.788 |
| Egypt | 8329.850 | 10542.455 | 6474.782 | 7.886 | 9.981 | 6.130 |
| Uganda | 8249.660 | 11488.589 | 5658.435 | 19.046 | 26.523 | 13.063 |
| Poland | 7938.861 | 8700.041 | 7126.467 | 20.761 | 22.752 | 18.637 |
| Iran (Islamic Republic of) | 7843.288 | 9779.872 | 3432.662 | 9.189 | 11.458 | 4.022 |
| Peru | 7798.765 | 10575.064 | 5516.753 | 21.502 | 29.156 | 15.210 |
| Spain | 7707.225 | 9239.887 | 6510.419 | 16.921 | 20.285 | 14.293 |
| Argentina | 7615.637 | 8934.514 | 6430.901 | 16.742 | 19.642 | 14.138 |
| United Republic of Tanzania | 7527.200 | 10971.033 | 5070.637 | 12.878 | 18.771 | 8.675 |
| Saudi Arabia | 6725.671 | 8948.031 | 4968.742 | 17.838 | 23.732 | 13.178 |
| Malaysia | 6609.171 | 8034.389 | 5600.619 | 20.776 | 25.256 | 17.605 |
| Canada | 6155.948 | 7394.976 | 5054.350 | 16.429 | 19.736 | 13.489 |
| South Africa | 5940.532 | 6946.924 | 5046.399 | 10.449 | 12.219 | 8.876 |
| Taiwan (Province of China) | 5934.233 | 6740.658 | 5142.129 | 25.106 | 28.518 | 21.755 |
| Venezuela (Bolivarian Republic of) | 5209.960 | 6793.016 | 3929.324 | 19.565 | 25.510 | 14.756 |
| Ecuador | 5195.441 | 6571.716 | 4037.524 | 28.759 | 36.377 | 22.349 |
| Iraq | 4747.872 | 6435.072 | 3482.917 | 11.517 | 15.610 | 8.449 |
| Algeria | 4578.939 | 5894.299 | 3314.973 | 10.360 | 13.336 | 7.500 |
| Nepal | 4464.618 | 6631.388 | 3038.020 | 14.342 | 21.302 | 9.759 |
| Location  Table(continued) | Number | Number_upper | Number_lower | Rate | Rate_upper | Rate_lower |
| Cambodia | 4459.051 | 6151.846 | 2843.750 | 26.162 | 36.094 | 16.685 |
| Democratic People's Republic of Korea | 4350.100 | 6010.156 | 3110.390 | 16.482 | 22.772 | 11.785 |
| Australia | 4317.960 | 5259.396 | 3466.647 | 16.742 | 20.392 | 13.441 |
| Mozambique | 4050.880 | 7148.592 | 2550.762 | 13.037 | 23.006 | 8.209 |
| Sri Lanka | 4043.588 | 5799.529 | 2524.128 | 18.157 | 26.042 | 11.334 |
| Democratic Republic of the Congo | 3735.341 | 6050.560 | 2319.928 | 4.150 | 6.722 | 2.577 |
| Zambia | 3695.860 | 7110.810 | 1725.235 | 18.938 | 36.437 | 8.840 |
| Romania | 3695.617 | 4596.426 | 2904.986 | 19.514 | 24.270 | 15.339 |
| Bolivia (Plurinational State of) | 3695.545 | 5128.519 | 2426.899 | 31.326 | 43.473 | 20.572 |
| Zimbabwe | 3552.108 | 4763.062 | 2371.868 | 22.777 | 30.542 | 15.209 |
| Morocco | 3472.135 | 4659.579 | 2535.650 | 9.340 | 12.534 | 6.821 |
| Chile | 3431.481 | 4038.875 | 2962.155 | 18.255 | 21.486 | 15.758 |
| Kazakhstan | 3221.808 | 3826.136 | 2644.042 | 16.997 | 20.185 | 13.949 |
| Madagascar | 3180.793 | 4358.054 | 2163.229 | 11.138 | 15.261 | 7.575 |
| Cuba | 3155.796 | 3717.002 | 2662.889 | 28.003 | 32.983 | 23.629 |
| Afghanistan | 3097.466 | 5106.179 | 1459.362 | 9.921 | 16.354 | 4.674 |
| Netherlands | 2942.751 | 3413.363 | 2455.075 | 17.098 | 19.833 | 14.265 |
| Sudan | 2658.764 | 4050.265 | 1707.266 | 6.123 | 9.328 | 3.932 |
| Belgium | 2412.246 | 2910.381 | 1982.548 | 21.032 | 25.375 | 17.286 |
| Kenya | 2343.105 | 3530.556 | 1703.010 | 4.680 | 7.052 | 3.402 |
| Malawi | 2325.527 | 3628.839 | 1483.745 | 11.958 | 18.659 | 7.629 |
| Guatemala | 2261.953 | 2613.757 | 1941.751 | 14.346 | 16.577 | 12.315 |
| Austria | 2260.176 | 2671.356 | 1874.707 | 25.163 | 29.740 | 20.871 |
| Belarus | 2159.588 | 2770.670 | 1692.947 | 23.160 | 29.713 | 18.156 |
| Somalia | 2148.146 | 3069.036 | 1383.615 | 9.942 | 14.204 | 6.404 |
| Portugal | 2128.213 | 2630.810 | 1720.657 | 20.063 | 24.801 | 16.221 |
| Greece | 2059.831 | 2305.570 | 1813.630 | 20.244 | 22.659 | 17.825 |
| Hungary | 2039.471 | 2490.716 | 1662.352 | 21.250 | 25.952 | 17.321 |
| Czechia | 2020.538 | 2538.369 | 1578.986 | 19.004 | 23.874 | 14.851 |
| Rwanda | 1899.781 | 2834.186 | 1295.132 | 14.316 | 21.358 | 9.760 |
| Serbia | 1825.168 | 2426.006 | 1328.188 | 20.463 | 27.199 | 14.891 |
| Uzbekistan | 1750.401 | 2371.130 | 1290.864 | 5.113 | 6.926 | 3.771 |
| Yemen | 1729.589 | 2456.462 | 1181.058 | 5.141 | 7.301 | 3.511 |
| Sweden | 1723.571 | 2081.199 | 1404.822 | 16.615 | 20.063 | 13.542 |
| Haiti | 1711.090 | 2385.495 | 1179.263 | 13.301 | 18.544 | 9.167 |
| Mali | 1657.267 | 2415.661 | 1137.565 | 6.875 | 10.022 | 4.719 |
| Bulgaria | 1633.385 | 2071.019 | 1291.693 | 24.067 | 30.515 | 19.032 |
| Israel | 1538.398 | 1855.664 | 1273.965 | 16.035 | 19.342 | 13.279 |
| Burundi | 1440.548 | 2229.204 | 922.459 | 10.896 | 16.862 | 6.978 |
| Tunisia | 1429.297 | 1963.896 | 996.986 | 12.069 | 16.583 | 8.419 |
| Location  Table(continued) | Number | Number_upper | Number_lower | Rate | Rate_upper | Rate_lower |
| Georgia | 1403.517 | 1700.351 | 1151.969 | 38.908 | 47.137 | 31.935 |
| Dominican Republic | 1400.963 | 1823.323 | 1057.729 | 12.721 | 16.556 | 9.604 |
| Syrian Arab Republic | 1392.435 | 2093.195 | 927.791 | 9.924 | 14.919 | 6.613 |
| Coted'Ivoire | 1390.785 | 1980.974 | 962.334 | 4.992 | 7.110 | 3.454 |
| Lao People's Democratic Republic | 1378.098 | 1922.221 | 889.099 | 18.680 | 26.055 | 12.051 |
| Angola | 1362.564 | 2151.090 | 835.324 | 4.166 | 6.577 | 2.554 |
| El Salvador | 1290.396 | 1605.401 | 1004.491 | 20.005 | 24.889 | 15.573 |
| Paraguay | 1238.835 | 1606.538 | 917.438 | 17.281 | 22.410 | 12.798 |
| Libya | 1213.610 | 1658.082 | 865.559 | 17.664 | 24.133 | 12.598 |
| South Sudan | 1208.171 | 1791.454 | 818.556 | 12.491 | 18.521 | 8.463 |
| Switzerland | 1207.332 | 1423.768 | 1009.565 | 13.531 | 15.956 | 11.314 |
| Jordan | 1171.258 | 1611.882 | 850.106 | 9.503 | 13.079 | 6.898 |
| Slovakia | 1167.646 | 1529.242 | 912.383 | 21.506 | 28.166 | 16.804 |
| United Arab Emirates | 1117.402 | 1470.078 | 843.947 | 11.602 | 15.264 | 8.763 |
| Azerbaijan | 1056.239 | 1420.978 | 768.052 | 10.059 | 13.533 | 7.315 |
| Finland | 1042.375 | 1255.012 | 858.782 | 18.829 | 22.670 | 15.513 |
| Nigeria | 1006.157 | 1797.090 | 645.956 | 0.435 | 0.777 | 0.279 |
| Costa Rica | 995.881 | 1159.555 | 852.115 | 20.975 | 24.422 | 17.947 |
| Eritrea | 992.587 | 1518.560 | 660.650 | 15.044 | 23.016 | 10.013 |
| Uruguay | 912.051 | 1096.382 | 764.068 | 26.781 | 32.193 | 22.436 |
| Norway | 896.888 | 998.531 | 791.024 | 16.554 | 18.430 | 14.600 |
| Armenia | 891.924 | 1144.246 | 676.314 | 29.778 | 38.202 | 22.579 |
| Croatia | 877.521 | 1041.255 | 738.445 | 20.850 | 24.741 | 17.546 |
| Kyrgyzstan | 866.419 | 1130.655 | 655.077 | 12.624 | 16.474 | 9.545 |
| Singapore | 848.498 | 1036.310 | 707.697 | 14.815 | 18.094 | 12.357 |
| Panama | 754.031 | 938.552 | 591.142 | 17.568 | 21.867 | 13.773 |
| Denmark | 737.899 | 875.959 | 617.098 | 12.610 | 14.969 | 10.545 |
| New Zealand | 723.122 | 843.794 | 590.465 | 13.988 | 16.322 | 11.422 |
| Lithuania | 719.918 | 876.552 | 586.892 | 26.387 | 32.128 | 21.511 |
| Cameroon | 714.560 | 1035.119 | 473.102 | 2.248 | 3.257 | 1.489 |
| Nicaragua | 706.203 | 886.095 | 532.996 | 10.589 | 13.287 | 7.992 |
| Lebanon | 695.819 | 915.462 | 551.153 | 12.559 | 16.523 | 9.948 |
| Honduras | 693.214 | 945.143 | 491.045 | 6.857 | 9.348 | 4.857 |
| Puerto Rico | 668.116 | 810.780 | 545.159 | 20.281 | 24.611 | 16.548 |
| Republic of Moldova | 660.814 | 783.306 | 557.099 | 18.389 | 21.798 | 15.503 |
| Papua New Guinea | 652.332 | 1084.831 | 352.681 | 6.236 | 10.370 | 3.371 |
| Ireland | 643.639 | 772.846 | 533.279 | 13.026 | 15.640 | 10.792 |
| Turkmenistan | 637.724 | 831.902 | 487.890 | 12.363 | 16.127 | 9.458 |
| Guinea | 625.069 | 892.894 | 438.306 | 4.654 | 6.649 | 3.264 |
| Latvia | 613.557 | 780.551 | 470.899 | 32.805 | 41.733 | 25.177 |
| Bosnia and Herzegovina | 570.684 | 738.682 | 428.192 | 17.278 | 22.365 | 12.964 |
| Jamaica | 523.284 | 688.608 | 391.420 | 18.689 | 24.594 | 13.980 |
| Location  Table(continued) | Number | Number_upper | Number_lower | Rate | Rate_upper | Rate_lower |
| Kuwait | 497.746 | 621.094 | 399.518 | 10.704 | 13.357 | 8.592 |
| Palestine | 452.579 | 561.249 | 285.465 | 8.813 | 10.929 | 5.559 |
| Albania | 443.956 | 631.144 | 318.834 | 16.641 | 23.657 | 11.951 |
| Mongolia | 436.166 | 576.105 | 333.753 | 13.073 | 17.268 | 10.004 |
| Burkina Faso | 430.394 | 597.348 | 272.178 | 1.891 | 2.624 | 1.196 |
| North Macedonia | 397.200 | 504.359 | 310.168 | 18.250 | 23.174 | 14.251 |
| Congo | 377.378 | 569.838 | 237.042 | 6.999 | 10.569 | 4.397 |
| Estonia | 331.093 | 420.687 | 252.606 | 25.258 | 32.093 | 19.271 |
| Slovenia | 326.351 | 428.062 | 238.958 | 15.768 | 20.683 | 11.546 |
| Niger | 323.903 | 472.177 | 203.428 | 1.294 | 1.886 | 0.812 |
| Trinidad and Tobago | 320.382 | 416.012 | 242.487 | 22.997 | 29.862 | 17.406 |
| Senegal | 315.064 | 475.153 | 209.552 | 1.987 | 2.996 | 1.321 |
| Ghana | 301.822 | 458.957 | 184.486 | 0.881 | 1.340 | 0.539 |
| Chad | 287.828 | 396.816 | 202.622 | 1.622 | 2.236 | 1.142 |
| Fiji | 275.306 | 383.990 | 173.704 | 29.780 | 41.536 | 18.790 |
| Central African Republic | 274.253 | 411.933 | 180.705 | 5.001 | 7.511 | 3.295 |
| Qatar | 255.927 | 361.845 | 173.139 | 8.597 | 12.154 | 5.816 |
| Oman | 240.192 | 314.986 | 160.249 | 5.106 | 6.696 | 3.407 |
| Benin | 218.524 | 319.705 | 144.016 | 1.619 | 2.368 | 1.067 |
| Namibia | 216.413 | 304.083 | 147.438 | 8.902 | 12.508 | 6.065 |
| Lesotho | 215.448 | 319.976 | 146.209 | 11.494 | 17.071 | 7.800 |
| Cyprus | 206.169 | 275.922 | 156.619 | 15.184 | 20.322 | 11.535 |
| Botswana | 196.186 | 309.564 | 124.092 | 8.197 | 12.935 | 5.185 |
| Bahrain | 191.649 | 256.648 | 138.830 | 12.529 | 16.779 | 9.076 |
| Timor-Leste | 186.058 | 266.403 | 119.695 | 13.311 | 19.060 | 8.564 |
| Togo | 177.331 | 270.922 | 113.084 | 2.118 | 3.236 | 1.351 |
| Djibouti | 174.270 | 292.054 | 104.600 | 13.845 | 23.203 | 8.310 |
| Mauritius | 165.843 | 181.072 | 150.155 | 13.038 | 14.236 | 11.805 |
| Sierra Leone | 161.025 | 237.478 | 98.362 | 1.816 | 2.678 | 1.109 |
| Montenegro | 156.202 | 195.177 | 123.341 | 25.273 | 31.579 | 19.956 |
| Gabon | 136.890 | 199.345 | 93.853 | 7.538 | 10.977 | 5.168 |
| Guyana | 132.800 | 174.873 | 99.052 | 17.365 | 22.867 | 12.952 |
| Eswatini | 132.548 | 196.328 | 82.099 | 11.474 | 16.995 | 7.107 |
| Comoros | 129.043 | 204.651 | 84.633 | 17.336 | 27.493 | 11.370 |
| Luxembourg | 128.623 | 146.362 | 112.653 | 19.964 | 22.718 | 17.485 |
| Liberia | 112.688 | 160.363 | 73.067 | 2.064 | 2.937 | 1.338 |
| Bhutan | 111.327 | 173.094 | 72.870 | 14.708 | 22.869 | 9.627 |
| Iceland | 102.585 | 124.086 | 85.761 | 29.278 | 35.414 | 24.476 |
| Malta | 93.712 | 113.371 | 77.386 | 21.191 | 25.637 | 17.499 |
| Bahamas | 91.600 | 114.979 | 73.370 | 23.610 | 29.636 | 18.911 |
| Suriname | 84.422 | 108.374 | 62.606 | 14.575 | 18.709 | 10.808 |
| Mauritania | 83.658 | 120.190 | 57.631 | 1.903 | 2.734 | 1.311 |
| Brunei Darussalam | 81.622 | 103.944 | 63.730 | 18.093 | 23.040 | 14.127 |
| Location  Table(continued) | Number | Number_upper | Number_lower | Rate | Rate_upper | Rate_lower |
| Barbados | 80.671 | 100.472 | 63.420 | 26.978 | 33.600 | 21.209 |
| Equatorial Guinea | 69.908 | 113.174 | 38.733 | 4.623 | 7.484 | 2.561 |
| Gambia | 68.792 | 94.444 | 47.549 | 2.873 | 3.945 | 1.986 |
| Solomon Islands | 56.124 | 82.733 | 35.268 | 8.211 | 12.103 | 5.159 |
| Guinea-Bissau | 52.184 | 72.756 | 37.550 | 2.528 | 3.525 | 1.819 |
| Saint Lucia | 48.748 | 58.833 | 40.242 | 27.462 | 33.143 | 22.670 |
| Saint Vincent and the Grenadines | 41.606 | 47.953 | 36.348 | 36.472 | 42.036 | 31.863 |
| Belize | 37.090 | 41.812 | 32.624 | 8.644 | 9.745 | 7.603 |
| Samoa | 34.340 | 48.860 | 25.111 | 16.074 | 22.870 | 11.754 |
| Maldives | 28.663 | 37.038 | 21.914 | 5.542 | 7.161 | 4.237 |
| Vanuatu | 26.103 | 34.545 | 17.396 | 8.340 | 11.037 | 5.558 |
| Grenada | 25.328 | 29.855 | 21.571 | 24.680 | 29.091 | 21.019 |
| Antigua and Barbuda | 20.991 | 22.381 | 19.500 | 23.481 | 25.036 | 21.813 |
| Bermuda | 20.858 | 26.205 | 17.114 | 32.826 | 41.241 | 26.935 |
| Cabo Verde | 20.370 | 40.609 | 4.063 | 3.642 | 7.261 | 0.727 |
| Guam | 19.375 | 22.883 | 14.623 | 12.170 | 14.374 | 9.186 |
| Andorra | 14.098 | 20.250 | 9.538 | 16.471 | 23.659 | 11.143 |
| Micronesia (Federated States of) | 13.110 | 18.349 | 8.643 | 12.780 | 17.888 | 8.425 |
| Dominica | 12.844 | 16.012 | 10.132 | 19.148 | 23.870 | 15.105 |
| American Samoa | 12.438 | 15.882 | 8.319 | 24.998 | 31.918 | 16.718 |
| Monaco | 10.904 | 13.785 | 8.270 | 28.797 | 36.408 | 21.842 |
| Saint Kitts and Nevis | 10.379 | 12.642 | 8.468 | 17.699 | 21.556 | 14.439 |
| Tonga | 9.604 | 13.519 | 6.024 | 9.035 | 12.718 | 5.667 |
| United States Virgin Islands | 9.147 | 11.964 | 6.802 | 10.648 | 13.927 | 7.918 |
| San Marino | 8.835 | 12.595 | 5.478 | 26.984 | 38.471 | 16.732 |
| Tajikistan | 8.751 | 12.037 | 6.019 | 0.086 | 0.118 | 0.059 |
| Seychelles | 8.565 | 10.337 | 6.930 | 8.123 | 9.805 | 6.573 |
| Greenland | 7.812 | 10.516 | 5.310 | 13.925 | 18.744 | 9.464 |
| Marshall Islands | 6.271 | 8.883 | 4.171 | 11.143 | 15.786 | 7.412 |
| Northern Mariana Islands | 5.500 | 6.484 | 4.305 | 11.343 | 13.372 | 8.878 |
| Sao Tome and Principe | 3.732 | 6.618 | 1.484 | 1.723 | 3.055 | 0.685 |
| Palau | 3.416 | 4.514 | 2.405 | 18.874 | 24.946 | 13.292 |
| Cook Islands | 2.857 | 4.048 | 2.190 | 16.098 | 22.807 | 12.341 |
| Tuvalu | 1.554 | 2.065 | 1.040 | 12.564 | 16.701 | 8.406 |
| Nauru | 1.411 | 2.084 | 0.846 | 12.801 | 18.902 | 7.672 |
| Kiribati | 0.586 | 1.078 | 0.378 | 0.483 | 0.890 | 0.312 |
| Niue | 0.328 | 0.423 | 0.223 | 19.619 | 25.298 | 13.323 |
| Tokelau  Abbreviations: ASR, age-standardized rate; DALYs, disability-adjusted life-years; TC, thyroid cancer. | 0.223 | 0.293 | 0.147 | 16.266 | 21.375 | 10.694 |
